# Separating Detection Performance from Thermodynamic Interpretability in Quantum-Capacitance Affinity Assays

**DOI:** 10.1021/acs.langmuir.6c03092

**Published:** 2026-09-25

**Authors:** Olivia Carr, Paulo Roberto Bueno

**Affiliations:** † Institute of Chemistry, Department of Physics and Mathematics, 28108 São Paulo State University (UNESP), Araraquara, SP 14800-060, Brazil; ‡ Centro Interdisciplinar de Química e Bioloxía (CICA) and Departamento de Química, Facultade de Ciencias, 153997 Universidade da Coruña, 15071 A Coruña, Galicia, Spain

## Abstract

Quantum capacitance, *C*_*q*_, is proportional to the electronic density of states of a redox monolayer and is used as a label-free transducer in affinity sensing. Here, we develop a theoretical framework and apply it to a reanalysis of previously published impedance data from a DNA-receptive interface; no new experimental data are reported. Under the isoscopic condition, the charging energy per accessible redox state is fixed at 2*k*_B_ *T*, so that the analyte dependence of the ensemble *C*_*q*_ resides in the number of accessible states. Binding renders a fraction χ of these states electrochemically inaccessible, which gives a relative response of 1/*C*_*q*_ equal to χθ/(1 – χθ) and an apparent affinity *K*_*a*_^app^ = (1 – χ)*K*_*a*_. Because the titration does not reach saturation, only the product χ*θ* is determined: χ is not identifiable, being bounded below at 0.169 (unamplified) and 0.625 (amplified with ferrocenecarboxylic acid, FcA), so that *K*_*a*_ cannot be recovered from *K*_*a*_^app^. The charge-relaxation resistance, 12.97 ± 0.72 kΩ, lies within 0.5% of *h*/2*e* ^2^ and is invariant across the unamplified titration, an internal consistency criterion that the amplified configuration fails. The Cole–Cole exponent of the electronic relaxation, α = 0.888 ± 0.042, is invariant with concentration but does not establish that the receptor sites are energetically equivalent. The calibration is log-linear over five decades, a shape no Langmuir isotherm reproduces, and equilibrium was not independently established; the extracted constants are therefore reported as apparent, protocol-dependent interfacial parameters. The analysis separates analytical detection performance, including the previously reported attomolar detection limit of the FcA-amplified interface, from thermodynamic interpretability, which requires an identifiable χ, an invariant *R*_*q*_ and a demonstrated equilibrium.

## Graphical abstract

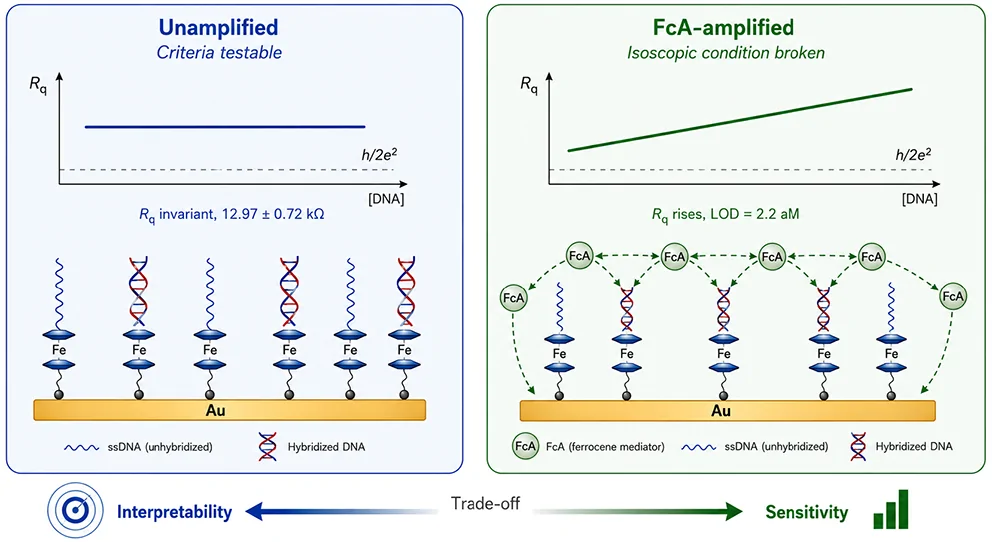

## Introduction

The quantification of molecular binding affinity constants (*K*_*a*_) at molecularly engineered surfaces constitutes a central challenge across biosensing, drug discovery, and the physical chemistry of interfaces.^1^ The classical framework for this task is the Langmuir adsorption isotherm, which describes the fractional occupancy θ of receptor sites (*R*_S_) on a surface upon exposure to a free ligand (L) in solution

1 $$\theta =\frac{K_{a}\left[L\right]}{1+K_{a}\left[L\right]}$$

The practical utility of eq 1 depends entirely on the nature of the transducer signal *S* that is proportional to θ. Optical methods such as surface plasmon resonance (SPR)—a kinetic, surface-based technique that measures association (*k*_on_) and dissociation (*k*_off_) rate constants from which *K*_*d*_ = *k*_off_/*k*_on_ is inferred indirectly—have long served this role;^2,3^ however, SPR sensitivity is limited for small-molecule and low-molecular-weight analytes, its instrumentation is bulky and costly, and its surface-dependent format introduces artifact risks that are difficult to exclude without independent solution-phase controls.^1,4,5^ Isothermal titration calorimetry (ITC), by contrast, is a solution-phase thermodynamic technique that directly measures the enthalpy, entropy, and Gibbs free energy of binding without surface immobilization, but it requires milligram-scale sample quantities and cannot provide analytical sensitivity metrics.^1^ The emergence of quantum-rate (QR) theory^6–9^ has established that electrochemical phenomena in electrolytic environments are governed by quantum electrodynamics (QED) principles, and that the quantum capacitance *C*_*q*_ of a redox interface—directly proportional to its electronic density of states (DOS)—constitutes an intrinsically sensitive, label-free transducer signal for affinity measurements.^1,10^ *C*_*q*_ can be measured at room temperature in physiological media using electrochemical impedance spectroscopy (EIS). No ultrahigh vacuum or cryogenic conditions are needed.^8^

The Langmuir isotherm is mathematically equivalent to the Hill equation with Hill coefficient *n* = 1, but the two are physically distinct: Langmuir was derived for independent, equivalent adsorption sites at a surface,^11^ whereas Hill describes cooperative binding to interdependent sites in homogeneous solution.^12^ The consequences of that distinction for surface-immobilized receptor platforms are set out in SI Section S1.7. In the quantum-capacitance framework the shape of the calibration curve bears on the adsorption regime, whereas the dispersion exponent α of the electronic relaxation does not by itself identify it (see “The Two Domains: Dispersion and Calibration Shape” section). It should be emphasized throughout that any constant obtained in this way is an *apparent* interfacial quantity, conditional on the model assumed at the surface and not identical to a bulk solution affinity measured by ITC. The standard Gibbs free energy of binding cannot be read from the logarithmic representation, in which the adsorption energy cancels from the equilibrium condition by construction (see “From the Langmuir Isotherm to Quantum Capacitance” section); it would follow from *K*_*a*_ only if χ were identified and equilibrium demonstrated.

The applications of *C*_*q*_-based electroanalysis span two interconnected domains that are central to biomedical science. In *diagnostics*, *C*_*q*_ has been validated as a transducer signal for nucleic acid detection, protein biomarker quantification, and whole-cell sensing, achieving detection limits in the attomolar range that rival or surpass those of optical and nanomaterial-enhanced platforms.^13–15^ Contributions from our group have established the quantum-rate framework as the interpretive foundation for these measurements,^6–8^ while the group of Professor Arben Merkoçi has independently extended electrochemical capacitance methodology to nanomaterial-integrated interfaces—laser-engraved reduced graphene oxide electrodes decorated with gold nanoparticles—demonstrating capacitive transduction of molecular recognition at electrode architectures beyond the redox-SAM paradigm.^16^ In *drug discovery*, affinity constants for candidate molecules binding to immobilized receptor targets are classically obtained by SPR^2,3^ and ITC.^17,18^ Under the quantum-rate (QR) conditions set out in the Theoretical Framework—that the interface operates as a low-dimensional scale structure (LDSS) satisfying the isoscopic condition *C*_*e*_ ≈ *C*_*q*_,^9^ that electron transport through the monolayer is diffusionless, and that EIS is recorded at the formal potential *V*_*F*_ of the redox couple—a single EIS titration yields *apparent, protocol-dependent interfacial constants*, at modest instrumental cost and in a miniaturizable format.^1,13^ These can be read as equilibrium affinity constants only when the interpretability conditions stated in the Theoretical Framework are met, and in the data analyzed here they are not.

Despite the growing body of experimental evidence for quantum-capacitance-based biosensing,^13–15^ a unified theoretical framework explicitly connecting the *C*_*q*_ signal to the Langmuir isotherm via statistical mechanics, and specifying the conditions under which a constant extracted from an EIS titration can be read thermodynamically, has not been presented. Establishing this connection is important not only for the interpretation of existing data but also for the rational design of next-generation affinity chips based on molecularly engineered surfaces—a field central to the scientific contributions of Professor Justin Gooding, in whose honor this Special Issue is dedicated.^19,20^

The present work addresses this gap. We first develop the theoretical framework, showing how the quantum energy *e* ^2^/*C*_*q*_ is related to the occupancy of the receptor sites, and identify the invariance of the quantum relaxation resistance *R*_*q*_ as an internal consistency criterion for the transduced signal. The two sensing configurations studied—unamplified and FcA-amplified—are depicted schematically in Figure 1. We then apply the framework to previously published EIS data from a DNA-receptive, redox-capacitive interface for HNSCC biomarker detection,^14^ reanalyzing the previously reported detection performance and extracting apparent interfacial constants from a single titration, together with the conditions under which such constants could be read as equilibrium affinities. The Results and Discussion section present these constants, the dispersion measurement and the *R*_*q*_ invariance test.

**Figure 1:**
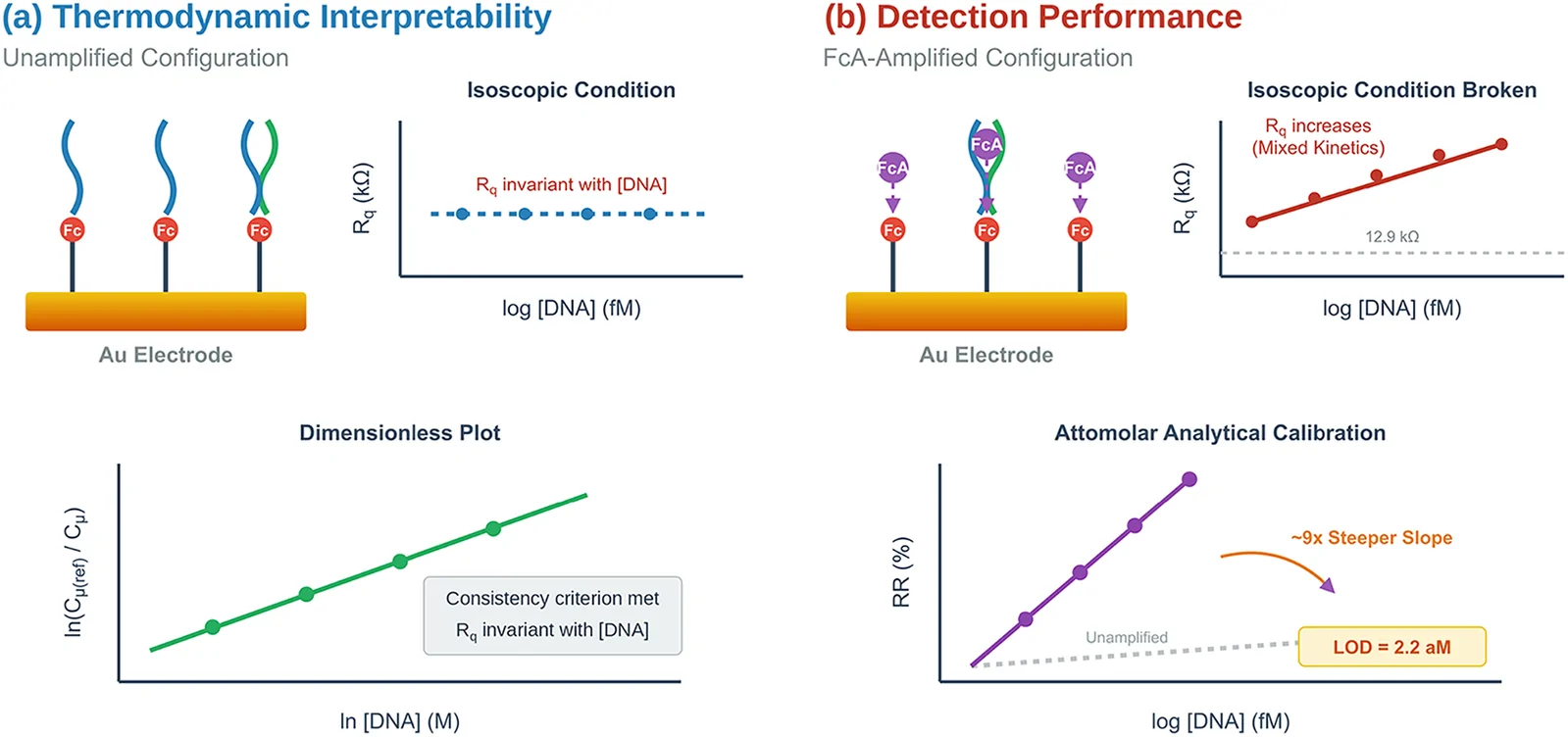
Schematic representation of the (a) unamplified and (b) amplified quantum-capacitive DNA sensing configurations. In (a), the LDSS moiety embedded in the redox-tagged peptide monolayer acts as the sole quantum transducer: DNA hybridization modulates the occupied redox states and thereby the quantum energy *E* = *e* ^2^/*C*_*q*_. In (b), 100 μM ferrocenecarboxylic acid (FcA, purple circles) is added to the electrolyte; alignment of the FcA/FcA^+^ formal potential with *E* = *e* ^2^/*C*_*q*_ opens additional electron-transmittance channels (dashed arrows), amplifying the capacitive response while introducing a kinetic contribution to the transducer signal. In both configurations, electron transport is *diffusionless*: it proceeds via quantum tunnelling through the monolayer in the absence of Warburg-type diffusion-limited kinetics.^8–10^ The quantum relaxation resistance of the interface is a series combination of the relaxation resistance *R_qt_* and the series resistance *R*_*s*_ (electrolyte and contact), the sum being what is classically termed the charge-transfer resistance *R_ct_*, and is obtained as *R*_*q*_ = 2π­(*R*_*s*_ + *R_qt_*).^9,21^ The factor 2π converts the series combination measured in the frequency domain into the relaxation resistance defined by the condition ω_*r*_τ = 1 at the characteristic frequency of the electronic relaxation; its origin is set out in SI Section S3.3. The symbols *R_qt_* and *R*_*s*_ are used consistently throughout for these two contributions. In the unamplified configuration, *R*_*q*_ is found invariant with analyte concentration, consistent with a tunnelling geometry unaffected by site occupancy. In the amplified configuration, the concentration-dependent change in *R*_*q*_ shows that the signal is no longer read through the monolayer channel alone; it is consistent with a perturbation of the FcA transmittance channels (SI Section S3.3), although electrostatic exclusion of the mediator by the duplex layer is not excluded. The bottom row of each panel shows schematic previews of the analytical outputs derived from the EIS titration data: for panel (a), the dimensionless representation of the relative quantum energy of the unamplified interface (ln­[*C*_μ_(ref)/*C*_μ_] vs ln­[L], Figure 5), which is log–linear across the range studied without reaching saturation; for panel (b), the logarithmic calibration of the amplified interface against that of the unamplified one (dotted line), showing the ∼9× steeper slope and the previously reported attomolar LOD.^14^ These schematic representations anticipate the quantitative results of Figures 4–6; in the *R*_*q*_ subpanel of (b) the horizontal dashed line marks the theoretical quantum resistance limit *R*_*q*_ ≈ 12.9 kΩ, not the experimental blank value (see Figure 6 for measured values).

### Scope of the Present Work

No new experimental data are reported: all impedance and voltammetric data analyzed here were acquired and published previously,^14^ where the analytical performance of the interface was established. The contribution is theoretical and reanalytical, and consists of the statistical-mechanics connection between the Langmuir isotherm and the quantum energy *e* ^2^/*C*_*q*_; the transduction parameter χ, which relates the measured signal to fractional occupancy and is bounded below in both configurations; the invariance of *R*_*q*_ as an internal consistency criterion for occupancy-dominated transduction; and the separation of *analytical detection performance* from *thermodynamic interpretability*, which are governed by χ with opposite sign: a larger χ improves detection but enlarges the correction between apparent and equilibrium constants, which can be undone only if χ is identified. Reprocessing procedures, fitting algorithms, error propagation and the per-concentration numerical basis are given in the Supporting Information, so that the quantities derived here can be reproduced from the original impedance data.

## Theoretical Framework

*Scope and underlying assumptions.* The quantum-rate (QR) framework developed below applies to electrode interfaces satisfying three conditions that together define quantum electroanalysis (QEA): (i) the interface behaves as a low-dimensional scale structure (LDSS) in which the electronic density of states is sufficiently discrete or confined that *C*_*q*_ is measurable and thermodynamically meaningful; (ii) electron transport across the monolayer is *diffusionless*, proceeding by coherent tunnelling rather than by Warburg-type diffusion, so that no mixed kinetic–diffusive contribution contaminates the impedance response; and (iii) EIS is performed at the formal potential *V*_*F*_ of the redox couple, where the isoscopic condition *C*_*e*_ = *C*_*q*_ holds.^9^

A thermodynamic reading of the resulting constants requires further conditions, which are properties of the particular measurement rather than of the framework: (iv) *R*_*q*_ must be invariant across the titration, an internal consistency criterion (see “Invariance of *R*_*q*_: An Internal Consistency Criterion” section); (v) the calibration must be an equilibrium occupancy curve; and (vi) the transduction parameter χ must be identifiable (see “Relating the Transducer Signal to Fractional Occupancy” section). In the present data (iv) is satisfied for the unamplified configuration and violated for the amplified one; (v) is not established, the calibration being log–linear over five decades and equilibrium untested; and (vi) is not met, χ being only bounded below. The constants are therefore reported as apparent, protocol-dependent calibration parameters. The derivations below presuppose (i)–(iii); conclusions drawn from them are qualified by (iv)–(vi) wherever they are used.

### From the Langmuir Isotherm to Quantum Capacitance

The statistical-mechanics derivation is given in full in the Supporting Information (SI, Section S1). It is summarized here in four steps, stating at each which capacitance applies: the capacitance per accessible state, *c*_*q*_ = *C*_*q*_/*N*, or the ensemble capacitance of the electrode, *C*_*q*_.

#### (i) Charging Energy per Accessible State

At the formal potential, where the Fermi–Dirac occupancy of the redox states is one-half, the directly measured electrochemical capacitance is *C*_μ_ = *Ne* ^2^/4*k*_B_ *T* and, under the isoscopic condition, *C*_*q*_ = *g*_*e*_ *C*_μ_ = *Ne* ^2^/2*k*_B_ *T*, with *g*_*e*_ = 2 and *N* the number of electrochemically accessible states. Written per state

2 $$\frac{e^{2}}{c_{q}}=\frac{Ne^{2}}{C_{q}}=2k_{B}T$$

which is independent of the ligand concentration. The distinction between single-microstate and ensemble capacitance, *C*_*q*_ = *Nc*_*q*_, is set out in ref 9.

#### (ii) Ensemble Capacitance

The quantity measured is the ensemble capacitance, for which eq 2 gives

3 $$\frac{e^{2}}{C_{q}}=\frac{2k_{B}T}{N}$$

Since the energy per state is fixed, the analyte dependence of *e* ^2^/*C*_*q*_ can enter only through *N*.

#### (iii) Accessible-State Count

Binding renders a fraction of the redox centers electrochemically inaccessible, so that *N* decreases with the occupancy θ of the receptor sites. This relation, *N* = *N*_0_(1 – *χθ*), is introduced in “Relating the Transducer Signal to Fractional Occupancy” section.

#### (iv) Adsorption Equilibrium

The occupancy follows from the equilibrium between the chemical potential of the receptor sites and that of the ligand in solution, μ_*s*_(θ) = μ_*L*_. For independent, equivalent sites

4 $$k_{B}T\ln\frac{\theta }{1-\theta }+\varepsilon =k_{B}T\ln\frac{\left[L\right]}{{\left[L\right]}_{0}}\Rightarrow \theta =\frac{K_{a}\left[L\right]}{1+K_{a}\left[L\right]},,K_{a}=\frac{1}{{\left[L\right]}_{0}}e^{-\varepsilon /k_{B}T}$$

where ε is the standard free energy required for binding per site and [L]_0_ = 1 M is the reference concentration. No capacitance enters eq 4.

Combining steps (ii) and (iii)

5 $$\frac{e^{2}}{C_{q}}\left([L]\right)=\frac{2k_{B}T}{N_{0}}\frac{1}{1-\chi \theta \left([L]\right)},,\ln\frac{C_{q,0}}{C_{q}}=-\ln\left(1-\chi \theta \right)$$

so that the ligand concentration enters the measured capacitance only through θ.

#### The Adsorption Energy Cancels by Construction

Substituting the solution of eq 4 back into the equilibrium condition returns an identity for any value of ε, because *K*_*a*_ is defined through ε. The cancellation is therefore a consequence of the parametrization and carries no physical content. Its only practical corollary is that no plot of an energy against ln­[L] can return *K*_*a*_ from its intercept; affinity information resides in the occupancy analysis of “Relating the Transducer Signal to Fractional Occupancy” section.

#### What the Model Predicts

The physical predictions of the framework are the following. (a) The form of the calibration, eq 11 below, which for Langmuir occupancy is a hyperbola in [L] with apparent affinity (1 – χ) *K*_*a*_; this prediction is *not* borne out over the range sampled here, where the response is log–linear (“The Two Domains: Dispersion and Calibration Shape” section). (b) Invariance of *R*_*q*_ when transduction is by occupancy of a fixed channel; satisfied in the unamplified configuration and violated in the amplified one (“Transducer Signal Consistency: Invariance of *R*_*q*_” section). (c) Invariance of the formal potential across the titration; expected for this architecture but not verified directly. (d) A charge-relaxation resistance at *h*/2*e* ^2^; the equivalent-circuit value is 12.97 ± 0.72 kΩ, and the relaxation frequency predicted from the measured capacitance (8.36 Hz) is close to the measured one (8.65 ± 0.11 Hz; SI Section S8.5).

#### Slope of the Logarithmic Representation

Differentiating eq 5

6 $$\frac{d\ln\left(e^{2}/C_{q}\right)}{d\ln\left[L\right]}=\frac{\chi }{1-\chi \theta }\frac{d\theta }{d\ln\left[L\right]}$$

a dimensionless quantity that depends on the interface through χ and on the isotherm through *d*θ/*d* ln­[L]. It is interface-specific rather than universal, and it is not used here to determine χ.

### Connection to the Density of States and Quantum-Rate Theory

Within the quantum-rate framework,^7,8^ the quantum capacitance is

7 $$C_{q}=e^{2}\frac{dn}{dE}=e^{2}\cdot \mathrm{DOS}$$

where *dn*/*dE* is the electronic density of states at the Fermi level of the interface. eq 7 holds at any interface whose electronic states are sufficiently confined that *C*_*q*_ is measurable—the class designated low-dimensional scale structures (LDSS), which includes quantum dots, molecular wires, graphene and reduced graphene oxide. The quantum-rate treatment has been applied to such structures previously,^22,23^ and electrochemical capacitance spectroscopy has been applied independently at reduced graphene oxide interfaces.^16^ The redox-active self-assembled monolayers used here are a chemically distinct realization of the same concept: the ferrocene groups create a density of states sharply concentrated at the formal potential *V*_*F*_, where the occupancy of the redox orbital passes through one-half and the isoscopic condition *C*_*e*_ ≈ *C*_*q*_ is met.^9^ Measuring the impedance-derived *C*_*q*_ as a function of analyte concentration at *V* = *V*_*F*_ is therefore a spectroscopic measurement of the change in density of states produced by molecular recognition, performed at room temperature in physiological media.^1^

The per-channel resistance quoted throughout, *h*/2*e* ^2^ = 12.906 kΩ, is the von Klitzing constant *h*/*e* ^2^ ≈ 25.8 kΩ of the earlier quantum-rate literature divided by the same electrochemical degeneracy *g*_*e*_ = 2. The correspondence between the directly measured *C*_μ_ and the *C*_*q*_ reported previously for interfaces of this class is set out in SI Section S3.4, and the origin of the estimator *R*_*q*_ = 2π­(*R*_*s*_ + *R*_*qt*_) in SI Section S3.3.

### Relating the Transducer Signal to Fractional Occupancy

The choice of *S* = 1/*C*_*q*_ as the transducer signal is not one of algebraic convenience: it follows from what the quantity represents. Within the quantum-rate framework the electrochemical energy of the interface is the change in chemical potential associated with charging the accessible states

8 $$E_{\mu }=\frac{e^{2}}{C_{\mu }}=g_{e}\frac{e^{2}}{C_{q}}$$

so that 1/*C*_*q*_ is proportional to an energy and not merely to a circuit element. The analytical observable is its relative change with respect to the blank

9 $$\mathrm{RR}\;\left(\%\right)=\frac{S_{i}-S_{0}}{S_{0}}\times 100=\frac{\left(1/C_{q,i}\right)-\left(1/C_{q,0}\right)}{1/C_{q,0}}\times 100$$

What that energy tracks is the *electronic occupancy* of the redox states: binding does not remove redox centers but renders a fraction of them electrochemically inaccessible, so the density of states addressable at *V*_*F*_ falls and the energy required to charge what remains rises.

The electronic occupancy is directly proportional to θ. Writing the number of electrochemically accessible states as

10 $$N\left([L]\right)=N_{0}\left(1-\chi \theta \right)$$

where *N*_0_ is the number of accessible states at the blank interface and χ ∈ (0,1] is the *transduction parameter*, the constant of proportionality between occupancy and the fraction of redox centers rendered electrochemically inaccessible. *N* counts the redox centers that remain electrochemically addressable at *V*_*F*_. Binding destroys none of them: the population of ferrocene centers is fixed by the monolayer, and what hybridization removes is their accessible occupancy. Using the transducer signal *S* = 1/*C*_*q*_ as defined in eq 9, the relative response becomes

11 $$\frac{\mathrm{RR}\;\left(\%\right)}{100}=\frac{N_{0}}{N_{i}}-1=\frac{\chi \theta }{1-\chi \theta }=\frac{\chi K_{a}\left[L\right]}{1+\left(1-\chi \right)K_{a}\left[L\right]}$$

#### Relation to the Usual Treatment

Equation 11 does not contradict the common practice of fitting an isotherm to the capacitive response; it specifies the conditions under which that practice is exact. The chain is linear at its origin: binding changes the electronic occupancy in direct proportion to θ (eq 10), and since *C*_*q*_ = 2*k*_B_ *TN*/*e* ^2^, the fractional change in capacitance is likewise strictly proportional to occupancy

12 $$\frac{C_{q,0}-C_{q}}{C_{q,0}}=\chi \theta$$

The nonlinearity enters only when this is expressed as a relative change of the reciprocal signal: RR(%)/100 = χθ/(1 – χθ), which reduces to χθ whenever χθ ≪ 1. The two configurations differ materially here. For the unamplified interface χθ rises from 0.045 to 0.169 across the titration, so that RR(%)/(100χθ) = (1 – *χθ*)^−1^ lies between 1.05 and 1.20; for the amplified interface χθ reaches 0.625 and the same factor reaches 2.67, so the linear reading fails outright there. All four values are computed from the capacitances of Table S4, which is the source of every quantity derived from the reanalysis in the frequency domain and of the products χθ; the isotherm constants of Table 2 are fitted instead to the published calibration of ref 14, as stated there. This is a further respect—independent of the *R*_*q*_ behavior described in “Transducer Signal Consistency: Invariance of *R*_*q*_” section—in which the amplified configuration is analytically superior but not thermodynamically interpretable. What eq 11 adds is the term beyond linear order in *χθ*, which matters as χ → 1: an isotherm fitted to RR(%) then returns *K*_*a*_^app^ = (1 – χ)*K*_*a*_ rather than *K*_*a*_, and χ is what makes that distinction explicit.

One consequence bears on constants already in the literature. Writing eq 11 in the linearized form [L]/(RR(%)/100) = 1/(χ *K*_*a*_) + (1 – χ)­[L]/χ shows that the *x*-intercept is – 1/[(1 – χ) *K*_*a*_]: the conventional linearization returns *exactly K*_*a*_^app^ = (1 – χ)*K*_*a*_, the same quantity as the nonlinear fit, and the factor (1 – χ) is of first order rather than second. Constants already in the literature are therefore biased by (1 – χ) in the same way and to the same degree as those reported here; what distinguishes the two routes is not this bias but the leverage of the linearization (“Apparent Interfacial Constants by Nonlinear Fitting” secrtion and SI Section S4.1). The saturation amplitude is *S*_m_ = 100χ/(1 – χ) throughout, reducing to 100χ only in the linear limit.

The measurement determines the product χθ and not χ alone, and no second relation available from these data separates the two: χ is not identifiable, and it is only bounded below (eq 15). SI Table S9 distinguishes the quantities read directly from the spectra from those that require the occupancy relation, an isotherm and equilibrium, or a known χ.

#### Reading Equation 11 When the Site Energies are Distributed

Equation 11 is written by substituting the Langmuir occupancy into eq 10; the step from occupancy to signal does not depend on which isotherm supplies θ. For a distribution of site energies the Temkin occupancy (eq 16) may be substituted instead, and the constant returned becomes *K*_0_, the affinity at the center of the distribution. Because both isotherms satisfy θ ≤ 1, the bound of eq 15 holds in either case. The symbol *K*_*a*_ denotes the equilibrium constant of the theory; every constant obtained from the data is an apparent interfacial constant—*K*_*a*_^app^ and *K*_*d*_^app^ for the Langmuir form, *K*_LL_ for the log–linear form—and not an equilibrium binding constant (“The Two Domains: Dispersion and Calibration Shape” section). We treat χ as a phenomenological parameter of the signal–occupancy relation and do not assign it a mechanistic interpretation: whether it reflects the geometry of the receptor–transducer pair, the kinetics of the recognition event, or both, is not determined by the present data.

#### Consequences

Equation 11 is a Langmuir hyperbola in [L] for Langmuir occupancy, and it identifies RR(%)—not *C*_*q*_, not 1/*C*_*q*_—as the quantity to which the isotherm applies. The apparent affinity returned by fitting is

13 $$K_{a}^{\mathrm{app}}=\left(1-\chi \right)K_{a}$$

so that *K*_*a*_ can be recovered from *K*_*a*_^app^ only if χ is known, χ ≪1 being the special case in which no correction is needed. Collecting the concentration-dependent terms puts eq 11 in the algebraically identical form

14 $$\mathrm{RR}\;\left(\%\right)=S_{m}\frac{K_{a}^{\mathrm{app}}\left[L\right]}{1+K_{a}^{\mathrm{app}}\left[L\right]},,S_{m}=\frac{100\chi }{1-\chi }$$

a plain hyperbola whose two parameters are the apparent affinity and the saturation amplitude. This is the form in which the fit is performed: it returns *K*_*a*_^app^ without any value of χ being assumed, and χ would follow from *S*_m_ alone were the plateau sampled. χ is therefore not a free parameter of the fit, and no other measurement in the present data supplies it.

What the measurement does determine is the product χθ = RR(%)/(100 + RR(%)). As a number it is the measured fractional change of capacitance, 1 – *C*_μ_/*C*_μ,0_; its reading as the product of a transduction parameter and an occupancy requires eq 10. Its largest value across the titration, computed from the capacitances of Table S4, is 0.169 (unamplified) and 0.625 (amplified). Since θ ≤ 1, these are lower bounds on the transduction parameter

15 χ≥0.169(unamplified),⁣χ≥0.625(amplified)

so that *K*_*a*_ = *K*_*a*_^app^/(1 – χ) exceeds the apparent constant by factors of at least 1.20 and 2.67. On the published calibration of ref 14, to which the constants of Table 2 are fitted, the corresponding bounds are χ ≥ 0.131 and ≥ 0.524, giving factors of at least 1.15 and 2.10. Because χ is not identified, only these lower bounds on the correction are available.

#### How the Apparent Constants are Obtained

The apparent constant follows from direct nonlinear least-squares fitting of eq 11, which uses the same observable as the theory and applies no variance-distorting transformation. Neither linearization in common use serves here: the logarithmic route cannot deliver *K*_*a*_ because *ε* cancels by construction (see above), and the Hanes–Woolf construction places logarithmically spaced concentrations on a linear abscissa, concentrating 82.5% of the abscissa variance on the single highest point (SI, Section S4.1). Because the titration does not reach saturation, the response constrains only the product χθ, so that χ and the affinity constant are not separately identifiable from the fitted saturation amplitude. The regression is therefore performed in the equivalent two-parameter form of eq 14, which returns the apparent affinity *K*_*a*_^app^ = (1 – χ)*K*_*a*_ without requiring a value of χ to be assumed; the equilibrium affinity would follow by the exact rescaling *K*_*a*_ = *K*_*a*_^app^/(1 – χ) if χ were known, and its sensitivity to χ is tabulated in SI, Section S4.2.

#### Detection Performance and Interpretability

*Analytical detection performance* and *thermodynamic interpretability* are distinct properties of an interface, governed by χ with opposite sign: a larger χ raises the calibration slope and lowers the detection limit, but simultaneously increases the discrepancy *K*_*a*_^app^ = (1 – χ)*K*_*a*_, which can be corrected only if χ is identified, and, in the amplified configuration, is accompanied by a concentration-dependent *R*_*q*_ that fails the internal consistency criterion. Sensitivity is therefore bought at the cost of interpretability—a design trade-off rather than a defect, provided it is stated.

#### Langmuir as the Ideal Limit of Temkin

The treatment above assumes a single site energy. For a uniform distribution of site energies of width Δε centered on the affinity *K*_0_, the occupancy is the Temkin isotherm

16 $$\theta =\frac{k_{B}T}{\Delta \varepsilon }\ln\frac{1+K_{0}e^{\Delta \varepsilon /2k_{B}T}\left[L\right]}{1+K_{0}e^{-\Delta \varepsilon /2k_{B}T}\left[L\right]}$$

With *x* = Δε/2*k*_B_ *T* and *a* = *K*_0_[L], the logarithm is odd in *x*, with first derivative 2*a*/(1 + *a*) at *x* = 0; it therefore equals 2*xa*/(1 + *a*) + *O*(*x* ^3^), and eq 16 reduces to the Langmuir isotherm as Δε → 0. For Δε ≫ *k*_B_ *T* and 1/*K*_max_ ≪ [L] ≪ 1/*K*_min_ it takes the familiar log–linear form θ ≃ 1/2 + (*k*_B_ *T*/Δ*ε*) ln (*K*_0_[L]), valid over a concentration factor *e* ^Δ*ε*/*k*_B_ *T*^ (SI, Section S8.1). Equation 16 satisfies θ ≤ 1 throughout.

In the frequency domain the analogous limit is that of a single electronic relaxation time. Writing the faradaic complex capacitance in the Cole–Cole form C*­(ω) = *C*_∞_ + (*C*_μ_ – *C*_∞_)/[1 + (*j*ωτ)^α^], with τ = *R*_*q*_ *C*_μ_, the exponent α measures the width of the distribution of electronic relaxation times, α = 1 corresponding to a single relaxation time. “Debye” here denotes single-time scale *electronic* relaxation at α = 1, not dielectric relaxation of the film. The two limits correspond only if the dispersion of relaxation times arises from the distribution of site energies, which the present data do not establish (“The Two Domains: Dispersion and Calibration Shape” section).

#### Which Quantum States are Probed

The states probed are those of the *redox-tagged peptide monolayer*—the ferrocene centers covalently tethered to the gold surface. The DNA target is not part of the electronic channel and forms no overlap of quantum states with the electrode. Nor is the measured quantity the double-layer capacitance, which is largely a solvent property and essentially featureless: it is the *faradaic* capacitance recorded at the formal potential of the ferrocene couple, where it exhibits a pronounced peak proportional to the number of electroactive states and absent at a nonredox interface. Transduction is therefore indirect and occupancy-based: hybridization at the receptor site sterically and electrostatically renders a fraction χ of those centers electrochemically inaccessible without removing or altering them. On this picture the formal potential is not expected to shift while the peak height changes, as observed for equivalent architectures,^24^ although this has not been verified across the present titration; the invariance of *R*_*q*_ is consistent with a change in the *number of accessible* states rather than in electronic coupling (“Invariance of *R*_*q*_: An Internal Consistency Criterion” section).

### Invariance of *R*_*q*_: An Internal Consistency Criterion

The quantum-rate description assigns different status to the two elements of the interfacial relaxation τ = *R*_*q*_ *C*_μ_. *R*_*q*_ is the charge-relaxation resistance of a coherent quantum channel and is fixed per channel at *h*/2*e* ^2^; it is not a dielectric loss term and does not carry thermodynamic information. *C*_μ_ is the electrochemical capacitance, proportional to the density of states, and is where the analyte dependence must appear. This is a structural feature of the description rather than a hypothesis about the interface, and it has an immediate experimental consequence, which is written

17 $$\frac{\partial R_{q}}{\partial \ln\left[L\right]}=0\Leftrightarrow R_{q}=\mathrm{const}\;\forall \left[L\right]$$

What eq 17 tests is therefore not whether the interface is quantum—that is the premise—but whether the signal being read is the quantum channel alone. *R*_*q*_ is set by the tunnelling geometry and the number of channels, neither of which is a function of how many receptor sites happen to be occupied; *C*_μ_ tracks the density of states and must evolve with [L]. If *R*_*q*_ is found invariant while *C*_μ_ moves, the observation is consistent with molecular recognition modulating the occupancy of the channel without altering the channel. If *R*_*q*_ moves with concentration, the channel structure itself is changing, and occupancy analysis cannot separate the two contributions. Because *R*_*q*_ and *C*_μ_ are obtained from distinct spectroscopic observables—the equivalent-circuit fit and the low-frequency plateau of *C*′ respectively—they are parametrically independent, and the test is not circular. Experimentally, *R*_*q*_ is obtained either from an equivalent-circuit fit or from the low-frequency expansion of the real admittance

18 Re[G*(ω)]≈RqCμ2ω2=RqCq24ω2

the two estimators and their respective domains of validity being set out in SI Section S8.

This is the feature that resistance-based and optical affinity assays lack. A recent re-examination of a widely cited C-reactive protein aptamer found that a *K*_*d*_ of 3.5 nM, originally determined by surface plasmon resonance, was attributable to nonspecific adsorption to the streptavidin/dextran chip: the protein bound with *K*_*d*_ ≈ 1.2 nM in the complete absence of immobilized aptamer.^4^ A systematic audit of 209 aptasensor publications found that none reported an auditable, construct-matched determination of *K*_*d*_ in homogeneous solution before device development.^5^ Without an internal criterion that distinguishes specific binding from surface-driven artifacts, erroneous affinities propagate. Equation 17 supplies an internal consistency criterion, and it is important to state what it can and cannot show. Invariance of *R*_*q*_ demonstrates that the relaxation resistance, and hence the tunnelling pathway, does not change detectably on binding; in the present data any systematic change across 10^3^–10^7^ aM is below about 5% of *R*_*q*_ (“Transducer Signal Consistency: Invariance of *R*_*q*_” section). It cannot exclude nonspecific adsorption or blocking that removes accessible centers without altering the relaxation of those that remain, for which a noncomplementary control is the relevant test; nor changes in *R*_*q*_ smaller than about 5%; nor small shifts of the formal potential.

A second condition must hold for eq 5 to be read as an occupancy relation: the formal potential *V*_*F*_ of the redox couple must not shift across the titration, ∂*V*_*F*_/∂[L] = 0. Measured at *V* = *V*_*F*_, a shift |Δ*V*_*F*_|≪ *k*_B_ *T*/*e* ≈ 25.7 mV perturbs *C*_μ_ only to second order by the symmetry of the Fermi–Dirac distribution, so the relation survives small shifts; the analysis is given in SI Section S1.4. In the present architecture the condition is expected on structural grounds, and it has been demonstrated directly for protein recognition at antibody-modified ferrocene SAMs of equivalent architecture, where *E*_*F*_/*e* remained constant across the analyte titration while only the density-of-states peak height changed;^24^ it has not, however, been verified by voltammetry across the present titration. This distinguishes the present platform from voltammetric anion sensors, in which the analyte is deliberately coupled to the redox center so that Δ*V*_*F*_ becomes the analytical signal.^25^

## Experimental Section

### Interface Fabrication

The DNA-receptive, redox-capacitive interface was fabricated on gold electrodes using established self-assembled monolayer (SAM) approaches, as described in full in ref 14. Briefly, the redox-tagged peptide SAM was assembled on the gold electrode surface, followed by immobilization of the single-stranded DNA (ssDNA) probe complementary to the HNSCC target sequence, and blocking with ethanolamine to minimize nonspecific binding.

### Electrochemical Measurements

All EIS measurements were performed using an AUTOLAB potentiostat (PGSTAT30) equipped with a frequency-response analyzer (FRA) in a three-electrode cell configuration (gold working electrode, Ag|AgCl (3 M KCl) reference, platinum mesh counter electrode). The electrolytic medium was 20 mM TBAClO_4_ in ACN/H_2_O (1:4 v/v). EIS was recorded at the formal potential *V*_*F*_ of the redox interface with a 10 mV RMS perturbation amplitude, scanning from 1 MHz to 10 mHz. All impedance spectra were acquired at room temperature, 25 ± 1 °C; the hybridization step described below was carried out at elevated temperature, but the electrode was returned to room temperature before measurement. The value *k*_B_ *T* = 25.7 meV used throughout the analysis corresponds to this measurement temperature and not to the hybridization temperature. Causality, linearity and stability of the spectra were verified by Kramers–Kronig analysis in the original study.^14^

The complex capacitance was obtained from the raw impedance data as C*­(ω) = 1/(jωZ*­(ω)), with real (*C*′) and imaginary (*C*″) parts calculated as *C*′ = – *Z*″/(|*Z*|^2^ω) and *C*″ = *Z*′/(|*Z*|^2^ ω). The electrochemical capacitance *C*_μ_ was read as *C*′ at the first local minimum of *C*″ on the low-frequency branch, that is, at the closure of the capacitive semicircle (its diameter), on the measured frequency grid and without interpolation, and the quantum capacitance obtained as *C*_*q*_ = 2*C*_μ_ under the isoscopic condition. The quantum relaxation resistance *R*_*q*_ was then extracted independently as *R*_*q*_ = 4Σ/*C*_*q*_^2^ from the ω^2^ slope Σ of Re­[G*­(ω)], regressed through the origin over the frequency window 0.105–0.956 Hz (unamplified) and 0.219–2.557 Hz (amplified), with *C*_μ_ read as *C* ^′^ at the closure of the capacitive semicircle, following the two-step procedure detailed in Section S3.2 of the Supporting Information.

### DNA Sensing Assays

Two biosensing configurations were employed: (i) a *conventional* (unamplified) setup using the *C*_*q*_ signal directly, and (ii) an *amplified* setup in which 100 μM ferrocenecarboxylic acid (FcA) was added to the electrolyte, with its formal potential aligned to the redox energy level of the interface to amplify the capacitive response.^10,14^ Target dsDNA concentrations ranged from 10^3^–10^8^ aM (= 1 fM–10^5^ fM = 1 fM–100 pM) (unamplified) and 1–10^5^ aM (amplified), dissolved in PBS/MgCl_2_ (pH 7.4). Hybridization was performed by incubating each concentration on the biosensor surface for 1 h at 83 °C, followed by cooling on ice for 5 min.^14^ These conditions were adopted from an earlier optimization carried out on a different genosensor interface,^26^ whose time- and temperature-dependent data were not published, and they were not reoptimized for the redox-peptide platform. The protocol is nonisothermal, and the impedance was measured at room temperature. Specificity was assessed using noncomplementary DNA (nc-DNA) sequences at the same concentrations.

## Results and Discussion

### Quantum Capacitance Response and Nyquist Diagrams

Figure 2 shows the EIS characterization of the unamplified sensing configuration for increasing target dsDNA concentrations from 10^3^ aM to 10^8^ aM (= 1 fM to 10^5^ fM = 1 fM to 100 pM). Figure 3 presents the equivalent analysis for the amplified (FcA-assisted) configuration at concentrations from 1 aM to 10^5^ aM.

**Figure 2:**
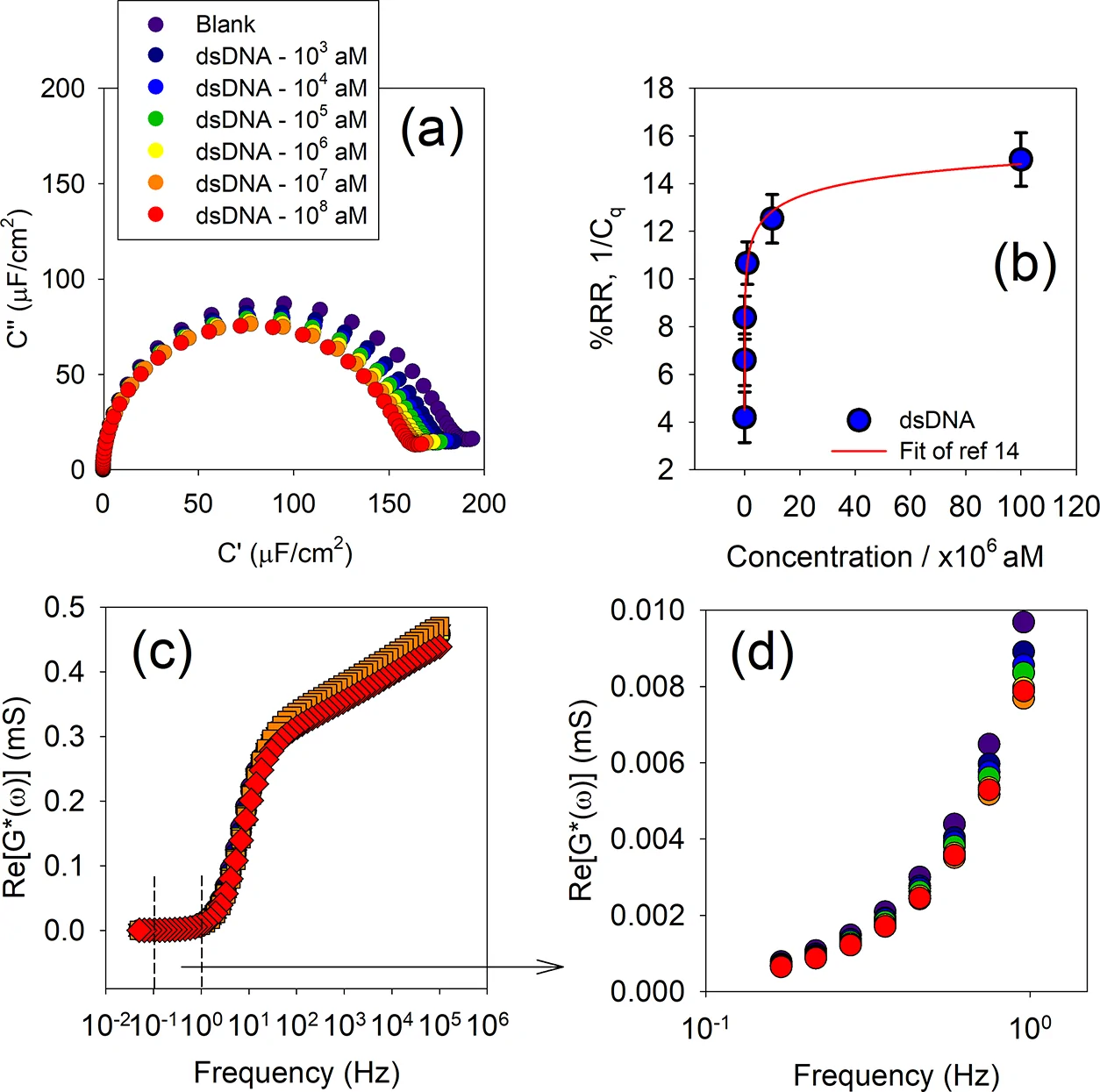
EIS characterization of the unamplified quantum-capacitive DNA biosensing configuration. (a) Nyquist capacitive diagrams (*C* ^″^ vs *C*′, μF cm^–2^) recorded at increasing target dsDNA concentrations from 10^3^ aM to 10^8^ aM (= 1 fM to 10^5^ fM = 1 fM to 100 pM). The systematic decrease in semicircle diameter reflects a decrease in *C*_*q*_ upon hybridization, consistent with a reduction in the number of accessible states (eq 5). (b) Relative response RR(%) against [DNA] on a linear axis, with the fitted curve reported in ref 14 (red line), which is nonzero at zero concentration and is not a Langmuir hyperbola; the Langmuir fits used in this work are those of Table 2. The apparent saturation in these coordinates is a property of the linear abscissa, on which logarithmically spaced concentrations are compressed toward the origin; the same data are log–linear across five decades. (c) Real part of the complex conductance, Re­[G*­(ω)] (mS), as a function of frequency (Hz) over the full spectral range (10^–2^–10^6^ Hz). (d) Low-frequency zoom of Re­[G*­(ω)] (mS) vs frequency (Hz). The curves decrease with [DNA] because their ω^2^ slope scales as *R*_*q*_ *C*_μ_^2^ and *C*_μ_ falls, while *R*_*q*_ = 4Σ/*C*_*q*_^2^ remains invariant (Figure 6). Data from ref 14.

**Figure 3:**
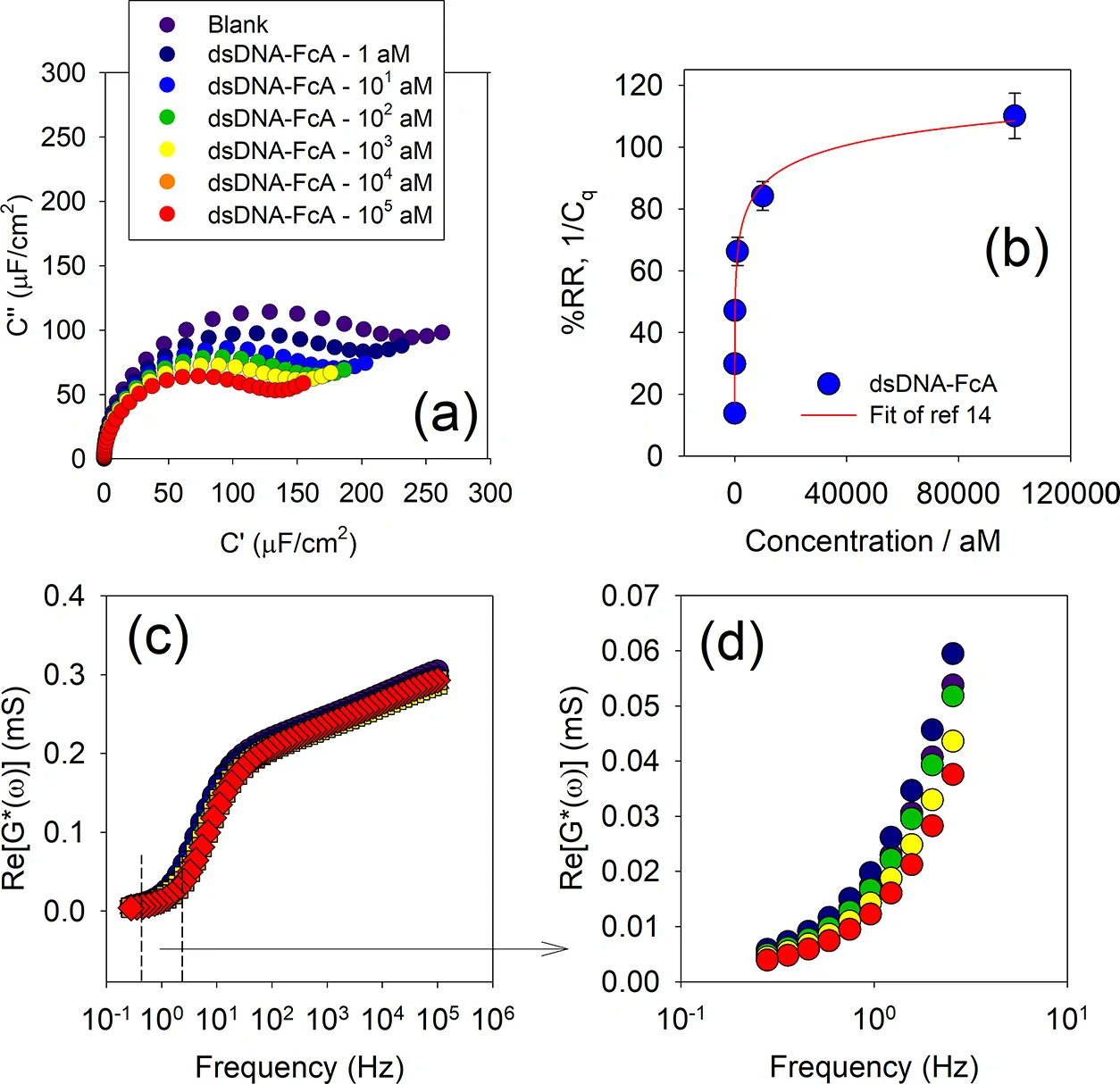
EIS characterization of the amplified (FcA-assisted) quantum-capacitive DNA biosensing configuration. (a) Nyquist capacitive diagrams (*C*″ vs *C*′, μF cm^–2^) recorded at increasing target dsDNA-FcA concentrations from 1 aM to 10^5^ aM. The larger absolute *C*_*q*_ values relative to the unamplified configuration (Figure 2) reflect the enhancement of the capacitive signal by alignment of the FcA redox probe with the interface energy state *E* = *e* ^2^/*C*_*q*_. The arc is more depressed than in Figure 2 and is followed by a slow low-frequency feature; its shape alone does not show whether the *R*_*q*_ consistency criterion is met (Figure 6). (b) Relative response RR(%) against [DNA-FcA] on a linear axis, with the fitted curve reported in ref 14, which is not a Langmuir hyperbola; the apparent constants of Table 2 are obtained instead by nonlinear least-squares. (c) Re­[G*­(ω)] (mS) vs frequency (Hz), full spectral range. (d) Low-frequency zoom of Re­[G*­(ω)] (mS) vs frequency (Hz). The curves decrease overall with concentration: their ω^2^ slope scales as *R*_*q*_ *C*_μ_^2^, and the fall in *C*_μ_ outweighs the rise in *R*_*q*_, which is obtained as 4Σ/*C*_*q*_^2^ and quantified in Figure 6. Data from ref 14.

The unamplified configuration shows a well-defined, near-ideal semicircular relaxation in panel (a); in the amplified one the arc is more depressed (α = 0.83) and is followed by a slow low-frequency feature (SI, Section S8.5). In both the semicircle diameter—that is, *C*_μ_—decreases systematically with target concentration, as hybridization reduces the electrochemical accessibility of the redox states. The Nyquist profile alone does not show whether the *R*_*q*_ consistency criterion is met (“Transducer Signal Consistency: Invariance of *R*_*q*_” section). A full description of the spectra, together with the surface-modification controls and the blank reproducibility, is given in SI Section S12.

Panels (b) of Figures 2 and 3 show RR(%) against concentration on a linear axis, with the fitted curve reported in ref 14. That curve is nonzero at zero concentration and is not a Langmuir hyperbola; the Langmuir fits used in this work are those of Table 2, and, as shown in “The Two Domains: Dispersion and Calibration Shape” section, the Langmuir form does not describe the calibration over the range sampled.

Panels (c) and (d) show Re­[G*­(ω)] over the full spectral range and at low-frequency zoom, providing the experimental basis for the ω^2^ extraction of *R*_*q*_ (eq 18). The curves are not expected to coincide: their ω^2^ slope is Σ = *R*_*q*_ *C*_μ_^2^, which falls with *C*_μ_ even at constant *R*_*q*_, and in both configurations the curves decrease overall with concentration. *R*_*q*_ is obtained for each spectrum as 4Σ/*C*_*q*_^2^, and its concentration dependence is quantified in Figure 6.

### Apparent Interfacial Constants by Nonlinear Fitting

Figure 4 presents the logarithmic calibration curves for both sensing configurations. A Hanes–Woolf linearization of the same calibration is shown as Figure S3 of the Supporting Information.

**Figure 4:**
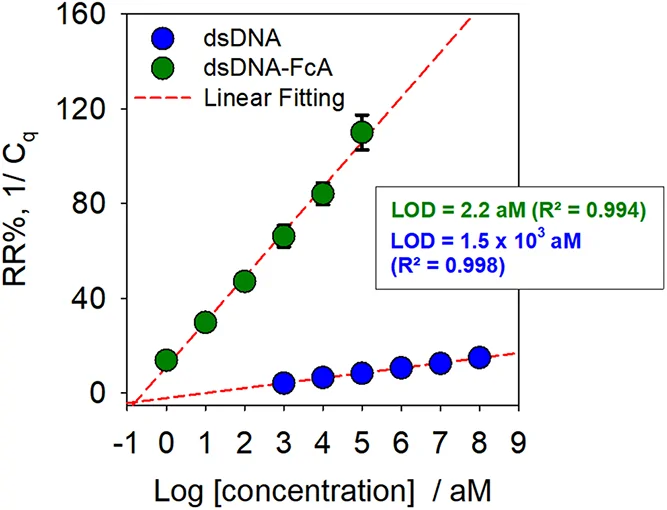
Logarithmic calibration curves (RR(%) vs log­[concentration] in aM) for both sensing configurations: unamplified (blue, previously reported LOD = 1.5 fM, *R* ^2^ = 0.998) and amplified (green, previously reported LOD = 2.2 aM, *R* ^2^ = 0.994). These coefficients are those of the calibration published in ref 14. The response is linear in log­[L] across five decades in both cases and does not reach saturation; as set out in “The Two Domains: Dispersion and Calibration Shape” section, no Langmuir isotherm reproduces this shape at any affinity. A Hanes–Woolf linearization of the same data is given as Figure S3 of the Supporting Information. This figure is related to Figure 5 by *e* ^2^/*C*_*q*_ = *E*_0_(1 + RR(%)/100) but is not the same plot: it replots the calibration of ref 14 (2.11% per decade), whereas Figure 5 is constructed from the capacitances reread in the present work (Table S4, 3.00% per decade). Both readings locate *C*_μ_ at the low-frequency minimum of *C*″; they differ in the order of averaging (per-electrode responses averaged in ref 14, mean capacitances here, with three rather than four electrodes at 10^8^ aM) and in the graphical rather than algorithmic location of the minimum (SI, Section S7). For the same reason, the products *χθ* quoted in “Relating the Transducer Signal to Fractional Occupancy” section are computed from the capacitances of Table S4 and not read from this panel. Data from ref 14.

That linearization—plotting [L]/RR(%) against [L] and reading—*K*_*d*_ from the *x*-intercept—is not used here because of its leverage. The titration concentrations are spaced logarithmically while the plot places them on a linear abscissa, so that the highest concentration alone carries 82.5% of the abscissa variance and the lowest four points 16.6% combined (SI, Section S4.1). The constant, obtained by extrapolation to an intercept lying outside the sampled range, is therefore fixed essentially by one measurement. The constants of Table 2 are obtained instead by direct nonlinear least-squares fitting of eq 11 to the untransformed calibration data, in the equivalent two-parameter form eq 14, which returns the apparent affinity *K*_*a*_^app^ = (1 – χ)*K*_*a*_; the rescaling to *K*_*a*_, which would require χ, is tabulated against χ in SI Section S4.2.

The difficulty is one of concentration spacing and sampled range rather than of the linearization itself: where a titration saturates, the intercept lies close to the sampled interval and the construction is well conditioned.^1^ The present one does not, and the two routes differ accordingly, 2.1 × 10^12^ against 1.5 × 10^14^ M^–1^.

### The Two Domains: Dispersion and Calibration Shape

The frequency and concentration domains of the measurement bear on the adsorption regime in different ways, and neither identifies an equilibrium binding constant.

In the *frequency* domain the Cole–Cole exponent of the faradaic relaxation is α = 0.888 ± 0.042 (unamplified), invariant with analyte concentration. Converted into a width of energies it corresponds to Δε ≈ 20 meV, that is 0.78 *k*_B_ *T* (SI, Section S8.2), but this conversion is only an order-of-magnitude upper bound on the heterogeneity expressed in the electronic relaxation, and it does not establish that the receptor sites are energetically equivalent.

#### Origin of the Dispersion

Relaxation-time dispersion at a modified polycrystalline gold electrode can arise from several sources other than a distribution of binding energies: geometric and microscopic heterogeneity of the electrode, the classical origin of constant-phase-element behavior;^27,28^ a distribution of tunnelling distances or electronic couplings; distributed capacitive behavior of the redox layer itself, that is, a spread of local formal potentials, coverages or lateral interactions;^29^ and overlap with the slower low-frequency process below the semicircle (SI, Section S8.6). The dispersion is already present at the blank, before any receptor site is occupied, and it does not change as sites are occupied (SI, Section S8.3). Neither observation favors an assignment to receptor-site energies, and none is made here.

In the *concentration* domain the calibration is log–linear across five decades, and no Langmuir isotherm reproduces that shape at any affinity. The two failure modes are exclusive: a small constant would make the response rise by some 3 orders of magnitude over the range, while a large one saturates it within the first two decades. Since *d *ln θ/*d* ln­[L] = 1 – θ for a Langmuir isotherm, a response rising by a factor 3.5 uniformly over 11.5 natural-logarithm units would require 1 – θ ≃ 0.11 to hold *constant* across five decades, which no such isotherm does. The fit reflects this: *R* ^2^ = 0.66 (unamplified) and 0.79 (amplified), with residuals showing the systematic U-shaped pattern of a saturating curve forced onto a logarithmic response, and the Akaike criterion preferring the log–linear form by ΔAIC = 31.4 and 20.5 (SI, Table S7).

#### Why an Isotherm Nonetheless Appears to Describe the Data

That panels (b) of Figures 2 and 3 appear to show saturation is a property of the representation and not of the interface. Logarithmically spaced concentrations plotted on a linear abscissa are compressed toward the origin, so a response rising uniformly with ln­[L] appears there as a steep rise followed by a level region—the shape of a saturating hyperbola—although the data contain no plateau. The apparent saturation is produced by the axis, and a high *R* ^2^ in these coordinates is not evidence that occupancy has saturated. Discrimination between the two forms must therefore be made in logarithmic coordinates, or by an information criterion on the untransformed response (Table S7).

#### Equilibrium and the Status of the Constants

Extraction of an equilibrium *K*_*a*_ from an isotherm fit requires the calibration to be an equilibrium occupancy curve, and the present data do not establish this. First, the hybridization protocol is not an equilibrium titration: each concentration is incubated for 1 h at 83 °C and cooled on ice, and the impedance is measured at room temperature, so that even an equilibrium reached during incubation would refer to a different temperature. Second, at the lowest concentrations the number of target molecules available is far too small to account for the observed response as equilibrium occupancy (SI, Section S9). Third, a calibration log–linear over five decades is not an equilibrium Langmuir isotherm. No incubation-time series, reversibility test or titration extended to saturation is available for these electrodes, and these are the experiments that would test the premise. The response is consistent with capture governed by delivered dose rather than by equilibrium occupancy, but the data do not settle this.

Accordingly, the constants obtained from either functional form are reported here as *calibration parameters*—apparent, protocol-dependent interfacial constants—and not as equilibrium binding constants. Fitting the log–linear form to the published calibration returns *K*_LL_ = 1. 0 × 10^17^ (unamplified) and 3.9 × 10^18^ M^–1^ (amplified); fitting the Langmuir form to the same data returns *K*_*a*_^app^ = 1.5 × 10^14^ and 1.4 × 10^16^ M^–1^. The Langmuir constants carry 95% confidence intervals of about one decade on either side (Table 2; SI, Table S8), because the hyperbola describes the data poorly and the data do not saturate within the range studied. A constant extracted under a model the data reject, or under one whose equilibrium premise is untested, should not be read as a thermodynamic quantity, and none of these values is so read in what follows.

- **Unamplified** (*C*_*q*_, dsDNA): 2.1 × 10^12^ M^–1^ by the Hanes–Woolf linearization (SI, Figure S3); 1.5 × 10^14^ M^–1^ by nonlinear fitting of the Langmuir form; 1.0 × 10^17^ M^–1^ by the log–linear form.
- **Amplified** (*C*_*q*_ + FcA, dsDNA-FcA): 1.3 × 10^15^; 1.4 × 10^16^; 3.9 × 10^18^ M^–1^, respectively.

The spread across these three routes, applied to one data set, is the quantitative statement of the point made above: within the concentration range sampled the data determine the *shape* of the response well and the underlying binding constant hardly at all. The increase in the apparent constant upon FcA amplification reflects not a change in the intrinsic thermodynamics of DNA hybridization but the greater sensitivity of the *C*_*q*_ transducer when the external redox probe is aligned with *E* = *e* ^2^/*C*_*q*_ of the monolayer.

Figure 4 overlays the logarithmic calibration curves for both configurations on the same axes—RR(%) vs log­[concentration] (aM)—illustrating the log–linear form of the response over the range studied. Both data sets are excellently linear (*R* ^2^ = 0.998 unamplified; *R* ^2^ = 0.994 amplified), and the nearly 9-fold improvement in slope (sensitivity) upon amplification is visually apparent from the steeper green regression line.

Whichever route returns them, these constants are *apparent* interfacial values, and they differ systematically from bulk solution affinities measured by ITC (in homogeneous solution) or by SPR (at optically sensed chip surfaces). Three physical factors operating at the solid–electrolyte interface enhance apparent affinity relative to bulk: (i) *molecular preorientation*, where the ssDNA probe is covalently anchored in an extended, solution-accessible conformation; (ii) *effective local concentration enhancement* arising from confinement of receptor sites within the two-dimensional monolayer plane; and (iii) *electrostatic preconcentration* induced at *V*_*F*_. These effects are well-established in surface-based affinity assays and are commonly invoked to explain why interfacial *K*_*d*_ values are systematically lower than their bulk counterparts. A cross-method comparison on this point has been reported for the quantum-rate transducer at antibody-modified redox-peptide interfaces, where *K*_*a*_ values for C-reactive protein, α-synuclein, NS1 and ADAM10 were placed alongside those from ELISA, isothermal titration calorimetry, biolayer interferometry and surface plasmon resonance;^1^ the quantum-rate values were the highest of the set for three of the four targets and comparable for the fourth. We cite that comparison as evidence of the direction and magnitude of the interfacial enhancement, and not as validation of the present work: it concerns antibody–protein recognition rather than the surface-tethered DNA hybridization studied here, and no matched side-by-side comparison on the present recognition system has been performed. Where the objective is to *rank* binding on a common platform rather than to obtain absolute values, apparent interfacial constants can serve, provided they are acquired under a common protocol and their model dependence is stated—conditions the present framework makes testable.

### Analytical Figures of Merit

The measurements analyzed here were made in hybridization buffer on target sequences related to head and neck squamous cell carcinoma. They are a proof of concept for the transducer, not a clinical validation: performance in serum or other biological matrices, where nonspecific adsorption and matrix effects operate, was not assessed in the original study and is not claimed here. Figure 4 presents the calibration curves as RR(%) vs log­[concentration] (aM) for both sensing configurations, emphasizing the diagnostic sensitivity performance. The two data sets are shown on the same axes to facilitate direct comparison, with the amplified configuration (green) exhibiting a markedly steeper slope and a dramatically lower LOD relative to the unamplified configuration (blue).

The analytical performance metrics are summarized in Table 1. For the unamplified configuration ref 14 reports an LOD of 1.5 fM (1500 aM) and an LOQ of 4.37 fM, with a calibration range of 10^3^–10^8^ aM. Signal amplification improves the performance, with reported values of 2.2 aM (LOD) and 15.8 aM (LOQ) and a calibration range of 1–10^5^ aM. This attomolar LOD is among the lowest reported for label-free DNA biosensors based on SAM-modified electrodes, achieved without enzymatic amplification or nanomaterial signal enhancement.^14^ These limits are taken as previously reported in ref 14 and have not been redetermined here. The lowest concentrations measured, 10^3^ aM (unamplified) and 1 aM (amplified), lie below the reported limits of detection; the limits were obtained from the logarithmic calibration and fall between its first and second points, and neither was checked by a measurement at the limit itself.

**Table 1:** Analytical Performance Metrics for the Quantum-Capacitive DNA Biosensing Assay (Unamplified and Amplified Configurations)^a^

Configuration	LOD	LOQ	Calibration Range
Unamplified (*C*_*q*_)	1.5 fM (1500 aM)	4.37 fM	10^3^–10^8^ aM (= 1 fM–100 pM)
Amplified (*C*_*q*_ + FcA)	2.2 aM	15.8 aM	1–10^5^ aM

a LOD and LOQ were determined as 3*s*/*m* and 10*s*/*m*, respectively, where *s* is the standard deviation of blank measurements and *m* is the slope of the analytical response against log­[DNA]. Both are quoted as previously reported in ref 14. and have not been re-determined here.

The analytical sensitivity—quantified as the slope of RR(%) against log­[DNA]—increases from 2.11% per decade (unamplified) to 18.94% per decade (amplified), representing a nearly 9-fold improvement in the rate of signal change per unit concentration change.^14^ This amplification factor is consistent with the QR signal amplification mechanism, in which alignment of the added FcA redox probe with the interface energy level *E* = *e* ^2^/*C*_*q*_ multiplicatively enhances the capacitive response per hybridization event.

#### The Logarithmic Representation

Figure 5 shows the same data in dimensionless form, ln­[*C*_μ_(ref)/*C*_μ_] versus ln­[DNA], an ordinate free of the normalization ambiguity that attends any plot of *e* ^2^/*C*_*q*_ in absolute energy units. It returns the relative slopes 1.161 × 10^–2^ (unamplified) and 6.670 × 10^–2^ (amplified) per natural-logarithm unit. By eq 5 its ordinate equals – ln­(1 – χθ) = ln­(1 + RR(%)/100), so the plot carries the same information as the calibration curve of Figure 4 and is drawn from the same spectra; its slope is given by eq 6, is interface-specific, and is not used to determine χ. The plot is therefore presented as a diagnostic of the transduction mechanism, the full treatment—including the statistical significance of each panel and the reasons for the differing regression statistics between the two representations—being given in SI Section S4.4.

**Figure 5:**
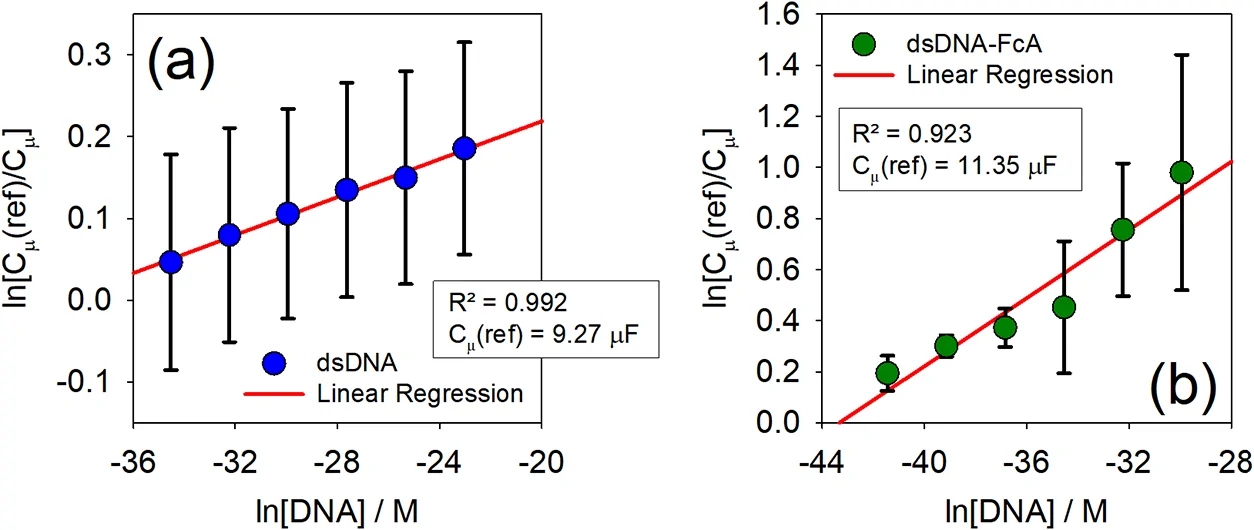
Dimensionless representation of the relative quantum energy of the interface, ln­[*C*_μ_(ref)/*C*_μ_] versus ln­[DNA], for (a) the unamplified dsDNA configuration and (b) the amplified dsDNA-FcA configuration. The ordinate is a ratio of capacitances and is therefore independent of the *C*_μ_/*C*_*q*_ convention: multiplying both terms by *g*_*e*_ leaves every point unchanged. The reference capacitances are those of the blank interface, *C*_μ_(ref) = *C*_μ,0_, i.e., 9.27 and 11.35 μF on the *C*_*q*_ = 2*C*_μ_ convention of Table S4. The fitted slopes are 1.161 × 10^–2^ and 6.670 × 10^–2^ per natural-logarithm unit, with *R* ^2^ = 0.992 and 0.923; the slope is given by eq 6 and is not used to determine the transduction parameter χ. The plot is presented as a diagnostic of the transduction mechanism rather than as a slope test: because ln­[*C*_μ_(ref)/*C*_μ_] = ln­[1 + RR(%)/100], it carries the same information as Figure 4 and is not independent evidence. Original analysis of the impedance data reported in ref 14.

The dependence of the quantum energy on ln­[L] enters through the occupancy θ (eq 5), and the intercept of this representation carries no affinity information, since the adsorption energy cancels from the equilibrium condition by construction. The apparent constants of Table 2 are obtained from the occupancy analysis (“Relating the Transducer Signal to Fractional Occupancy” section).

**Table 2:** Apparent Interfacial Constants Obtained from the Calibration^a^ ^,^ ^b^

Configuration	Constant (M^–1^)	log_10_ constant (95% CI)	*K*_*d*_^app^	*R* ^2^
*Langmuir form*, *K*_*a*_^app^				
Unamplified	1.5 × 10^14^	14.18 ± 1.04	6.6 fM	0.66
Amplified	1.4 × 10^16^	16.14 ± 1.00	73 aM	0.79
Log–linear form, *K*_LL_				
Unamplified	1.0 × 10^17^	17.02 ± 0.28	—	0.998
Amplified	3.9 × 10^18^	18.59 ± 0.40	—	0.993

a All constants are obtained by direct nonlinear least-squares fitting to the calibration published in ref 14. (2.11 and 18.94% per decade), and not to the capacitances re-read in Table S4, from which the dispersion analysis and the products *χθ* are computed. The Langmuir constants are the apparent affinities *K*_*a*_^app^ = (1 – χ)*K*_*a*_ returned by eq 14; *χ* is not identifiable, and on this calibration *K*_*a*_ would exceed *K*_*a*_^app^ by factors of at least 1.15 (unamplified) and 2.10 (amplified) (SI, Section S4.2). Intervals are 95% confidence intervals (Student *t*, *n* – 2 = 4 degrees of freedom); parameter correlations and goodness-of-fit statistics are given in SI, Tables S7 and S8.

b Both sets are reported as *calibration parameters*. The Langmuir form assumes equivalent independent sites at equilibrium; neither premise is established by the present data (“The Two Domains: Dispersion and Calibration Shape” section). The log–linear form describes the data well (ΔAIC = 31.4 and 20.5) but is a shape rather than a mechanism: no dissociation constant is quoted for it, its constant not being a half-saturation point.

### Transducer Signal Consistency: Invariance of *R*_*q*_

Figure 6 plots *R*_*q*_ against log­[DNA] for both configurations. The two behave qualitatively differently, and the difference is the basis on which the thermodynamic reading is restricted to one of them.

**Figure 6:**
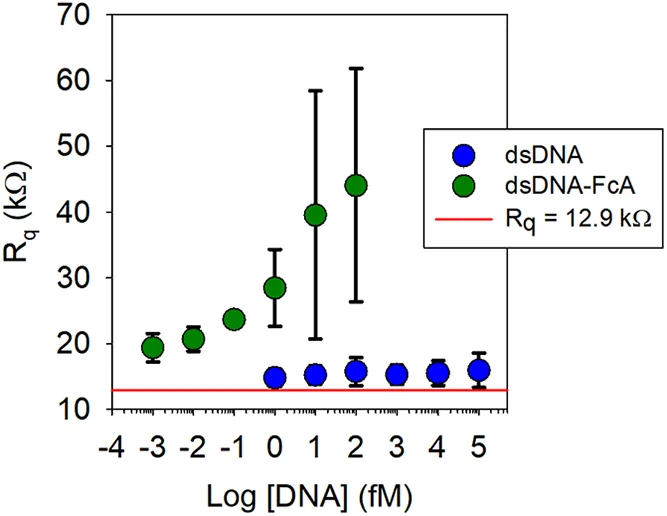
Quantum relaxation resistance *R*_*q*_ (kΩ) plotted as a function of log­[DNA] (fM) for the unamplified (blue circles, dsDNA) and amplified (green circles, dsDNA-FcA) sensing configurations. The values plotted are those of the low-frequency estimator *R*_*q*_ = 4Σ/*C*_*q*_^2^ (eq 18); the equivalent-circuit estimator *R*_*q*_ = 2π­(*R*_*s*_ + *R*_*qt*_), which resolves the faradaic branch alone and gives 12.97 ± 0.72 kΩ for the unamplified configuration, is reported in the text and Table S4. For the amplified configuration the low-frequency estimator lies outside its domain of validity, the extraction window overlapping a second, slower relaxation whose amplitude in that configuration exceeds the capacitance of the faradaic branch itself (SI, Sections S8.5 and S8.6). The green series should therefore be read as a qualitative indication of the trend, the quantitative trend being the +1.10 ± 0.25 kΩ per decade returned by the equivalent-circuit estimator. The criterion applied throughout is the invariance of *R*_*q*_ across the titration, not the absolute value returned by either estimator. The horizontal red line marks the theoretical quantum resistance limit *R*_*q*_ = 1/*G*_0_ ≈ 12.9 kΩ.^21^ The unamplified values are statistically flat and close to the quantum limit, meeting the internal consistency criterion ∂*R*_*q*_/∂ ln­[L] = 0 (eq 17) and consistent with an occupancy-dominated origin for the *C*_*q*_ signal. The amplified values rise with concentration and fail the criterion; the rise is consistent with a modification of the FcA transmittance channels,^10^ although electrostatic exclusion of the mediator by the duplex layer is not excluded. Error bars represent standard deviation across independently fabricated electrodes. Original analysis of the impedance data reported in ref 14; this figure does not reproduce any previously published figure.

#### Unamplified Configuration

*R*_*q*_ is statistically flat across the whole concentration range, from 10^3^ aM to 10^8^ aM. The equivalent-circuit estimator gives 12.97 ± 0.72 kΩ, within 0.5% of *h*/2*e* ^2^ = 12.906 kΩ and flat across the four decades of concentration sampled by that estimator, 10^3^–10^7^ aM (SI, Table S4); the low-frequency estimator, which additionally covers 10^8^ aM, gives 15.34 ± 0.43 kΩ between concentrations, also flat, the two differing in absolute value for the reasons set out in SI Section S8.5. That *R*_*q*_ sits at *h*/2*e* ^2^ is consistent with the relaxation being that of the quantum channel of the redox monolayer. That it does not move while *C*_μ_ falls by 17% shows that hybridization does not detectably alter the relaxation resistance: the trend of the equivalent-circuit estimator across 10^3^–10^7^ aM is −0.05 ± 0.03 kΩ per decade (*p* = 0.22; 95% interval [−0.16, + 0.05] kΩ per decade), so any systematic change is below about 5% of *R*_*q*_. The internal consistency criterion ∂ *R*_*q*_/∂ ln [L] = 0 is therefore met, and the response is consistent with occupancy-dominated transduction. The criterion does not exclude nonspecific blocking of accessible centers, for which the noncomplementary control below is the relevant test, and it does not by itself license a thermodynamic reading of the constants extracted (“The Two Domains: Dispersion and Calibration Shape” section).

#### Amplified Configuration

*R*_*q*_ rises monotonically across the range sampled. The equivalent-circuit estimator, whose domain of validity is not in question here, increases from 18.8 kΩ at the blank to 24.9 kΩ at 10^5^ aM, a trend of +1.10 ± 0.25 kΩ per decade that is statistically significant (*p* = 0.012; SI, Table S6). This fails the consistency criterion. The amplification operates by aligning the FcA/FcA^+^ formal potential with the interfacial Pep-Fc states, which opens additional diffusionless transmittance channels.^10^ One interpretation of the rise in *R*_*q*_ is that DNA hybridization perturbs that alignment, altering the electronic coupling and the number of active channels (SI, Section S3.3). The data do not exclude two alternatives: electrostatic and steric exclusion of the mediator by the growing duplex layer, which would also reduce the number of active channels; and covariance between the faradaic and slow low-frequency branches in the equivalent-circuit fit, whose dispersion at the highest concentration is large. Whatever its origin, the constant obtained from the amplified series is of mixed origin and its interpretation is correspondingly restricted, while the analytical figures of merit—which do not depend on that separation—remain valid.

The operational significance of the test extends beyond the present assay. Where affinities are to be compared across compounds on one platform, a criterion is needed that distinguishes a genuine change in site occupancy from a change in the transport properties of the film. Equation 17 provides a necessary consistency check from the same spectra that give the calibration, and the present results show that it discriminates between configurations whose gain in sensitivity has and has not been accompanied by a change in the relaxation resistance.

### Detection Performance and Interpretability from a Single EIS Data Set

Analytical detection performance and thermodynamic interpretability are distinct properties of an interface, governed by the transduction parameter with opposite sign, and unless that parameter is identified independently they are not maximized by the same design. Both are assessed from a single EIS concentration-titration experiment, without separate protocols—the logarithmic calibration curve (Figure 4) supplying the detection metrics and the occupancy analysis the bound on the transduction parameter and the apparent interfacial constants, from the same *C*_*q*_([L]) data set. The *R*_*q*_ criterion required for the second is met, however, only in the unamplified configuration: *R*_*q*_ is invariant there and concentration-dependent in the amplified case, so the constant returned by the latter is an apparent value of mixed thermodynamic and kinetic origin. Meeting that criterion is necessary and not sufficient: χ is not identifiable in either configuration, and the equilibrium premise is untested in both, so the second property is delivered in full by neither. The two configurations are complementary rather than equivalent.

#### Sensitivity and Interpretability

The femtomolar sensitivity of the unamplified configuration (previously reported LOD = 1.5 fM) is accompanied by *R*_*q*_ invariance, so its response is consistent with occupancy-dominated transduction. The attomolar sensitivity of the amplified configuration (previously reported LOD = 2.2 aM) extends the analytical reach by 3 orders of magnitude, but the concentration-dependent *R*_*q*_ signals that the *C*_*q*_ response there has mixed origin, and its apparent constant should be read as an effective amplified calibration parameter. Note that the constants themselves are model-dependent to a degree that limits what may be read from them: the values returned under the Langmuir form (1.5 × 10^14^ and 1.4 × 10^16^ M^–1^) and under the log–linear one (1.0 × 10^17^ and 3.9 × 10^18^ M^–1^) differ by two to 3 orders of magnitude on the same data, and both are reported as calibration parameters.

#### Comparison with Established Methods

The two configurations serve different ends from the same measurement, and it is useful to place them against the established alternatives. Surface plasmon resonance measures the kinetic constants *k*_on_ and *k*_off_, from which *K*_*d*_ follows indirectly;^2,3^ its surface format carries systematic artifact risks, and elaborate negative controls are needed to exclude nonspecific contributions.^4,5^ Isothermal titration calorimetry is the solution-phase reference method and provides a complete thermodynamic characterization (*K*_*a*_, Δ*H*, Δ*S*, *n*) in homogeneous solution,^17,18,30^ but requires milligram quantities at micromolar-to-nanomolar concentrations^1^ and yields no analytical sensitivity metrics.

The quantum-capacitance measurement operates at attomolar-to-femtomolar concentrations and returns detection metrics and apparent interfacial constants from one titration. Two qualifications should accompany that statement. First, the constants obtained are *interfacial* and protocol-dependent: they describe binding to an immobilized receptor under the conditions of the assay, and are not bulk solution affinities. Whether they agree with solution-phase values measured by ITC on matched recognition systems is an open question that these data cannot address. Second, the *R*_*q*_ invariance test provides an internal consistency check on whether the response is occupancy-dominated—a check that is available because the measurement is impedimetric, and one we are not aware of having an analogue in the optical methods, though we make no claim to have surveyed them exhaustively.

#### Miniaturisation and Sample Economy

The measurements analyzed here were made in a conventional three-electrode cell on modified gold electrodes, with interelectrode dispersion assessed across four independently prepared electrodes.^14^ The capacitive format is nonetheless intrinsically miniaturizable and economical in sample: ITC, by comparison, requires 200–300 μL per injection series at micromolar-to-nanomolar concentrations,^1^ which is a material constraint for scarce targets. Integration with microfluidics would allow automated injection of compound series and extend the measurement to *k*_on_/*k*_off_ determination. These are prospective advantages of the format rather than of the transduction principle, and we note them as such.

The specificity of the assay was confirmed by the much smaller response to noncomplementary DNA sequences (nc-DNA) at the same or higher concentrations, with the ds-DNA signal exceeding the nc-DNA response by a factor of approximately 4 even when the nc-DNA concentration was 10-fold higher.^14^ Concretely, in the unamplified configuration, the RR% recorded for 10^3^ aM ds-DNA (≈ 4.35%) was approximately four times greater than that observed for 10^3^–10^4^ aM nc-DNA; in the amplified configuration, the RR% for 1 aM ds-DNA-FcA (≈ 14.62%) was similarly ≈ 4× higher than the nc-DNA-FcA signal at 1 and 10 aM.^14^ This selectivity factor, maintained across both sensing configurations, indicates that the response is dominated by specific hybridization; it is this control, rather than the invariance of *R*_*q*_, that addresses nonspecific adsorption.

## Conclusions

Quantum capacitance is an interpretable transducer signal for affinity sensing at molecularly engineered electrode surfaces, provided the conditions under which that interpretation holds are stated and tested. The main findings are the following.

1. The Langmuir isotherm is the homogeneous limit of the Temkin isotherm for a uniform distribution of site energies, recovered from its full expression as Δε → 0. In the frequency domain the analogous limit is a single electronic relaxation time (α = 1); the two limits correspond only if the dispersion of relaxation times arises from the site energies, which the present data do not establish.
2. For the present interface the calibration is log–linear across five decades, which no Langmuir isotherm reproduces at any affinity. The Cole–Cole exponent, α = 0.888 ± 0.042, is invariant with concentration but does not establish that the receptor sites are equivalent, and equilibrium was not independently tested: the hybridization protocol is nonisothermal and the material balance at low concentration is unfavorable. The extracted constants are therefore reported as apparent, protocol-dependent calibration parameters. Three routes applied to the same data return constants spanning about 5 orders of magnitude (2.1 × 10^12^, 1.5 × 10^14^ and 1.0 × 10^17^ M^–1^ for the unamplified series), which measures how weakly a binding constant is determined over an unsaturated range.
3. The quantity related to occupancy is the relative response of the reciprocal quantum capacitance, not *C*_*q*_ itself. The charging energy per accessible state is fixed at 2*k*_B_ *T*, so the analyte dependence of the ensemble capacitance resides in the number of accessible states. Binding renders a fraction χ of the accessible redox states electrochemically inaccessible—the centers persist and only their accessible occupancy is lost—giving eq 11, and the apparent affinity is related to the true one by *K*_*a*_^app^ = (1 – χ)*K*_*a*_, so that an equilibrium reading requires χ to be identifiable. The measurement determines only the product *χθ*, which bounds χ from below (eq 15); χ is not identified in either configuration. The adsorption energy cancels from the equilibrium condition by construction, so the logarithmic intercept carries no affinity information, and Δ*G*° cannot be obtained from it.
4. The charge-relaxation resistance is the structural element of the quantum-rate description: fixed per channel at *h*/2*e* ^2^, carrying no thermodynamic information, and its constancy is an internal consistency criterion for reading the analyte through the quantum channel. Measured here it is 12.97 ± 0.72 kΩ against *h*/2*e* ^2^ = 12.906 kΩ, and invariant across the titration in the unamplified configuration; it is not invariant in the FcA-amplified one, which separates two properties commonly conflated: detection performance, which improves with larger χ, and thermodynamic interpretability, which requires an identifiable χ, an invariant *R*_*q*_ and a demonstrated equilibrium. The amplified configuration reaches a previously reported LOD = 2.2 aM and fails the *R*_*q*_ criterion; the unamplified configuration satisfies it at a previously reported LOD = 1.5 fM. Satisfying it is necessary but not sufficient: χ is not identified in either configuration, and the equilibrium premise is untested in both.

The framework applies beyond redox-tagged SAMs to any low-dimensional scale structure in which a confined electronic density of states renders *C*_*q*_ measurable and sensitive to molecular recognition.^1,16,22,23^ What the present analysis adds is a set of conditions under which its output may be read thermodynamically, and a demonstration that these conditions are testable on the same data that provide the analytical calibration—including the case, encountered here, in which they are not met.

## Supplementary Material



## Data Availability

The data that support the findings of this study are available within the article and its Supporting Information. Additional raw data are available from the corresponding author upon reasonable request.

## Notes

Statistical-mechanics derivation of the Langmuir isotherm and its connection to the quantum energy; relation to the Hill isotherm and its extrapolation to surfaces; decomposition of the capacitance into occupancy and formal-potential channels; derivation of the transduction parameter χ and the signal–occupancy relation; DFT connection to chemical hardness; isoscopic framework and extraction of *R*_*q*_; nonlinear least-squares procedure for the apparent interfacial constants, with confidence intervals, and the status of the logarithmic representation; dispersion of the electronic relaxation and the correspondence between the frequency and concentration domains; per-concentration numerical basis and robustness of the extraction; scope and limitations, with the status of the reported quantities; list of symbols; expected spectroscopic signatures and diagnostic guide for nonideal behavior; full description of the impedance spectra of the two configurations, with surface-modification controls and blank reproducibility; cyclic voltammograms, Nyquist diagrams of the surface-modification steps, Hanes–Woolf linearization, and Re­[*G**] against ω^2^ (PDF)

## Abbreviations

QR: quantum rate
QED: quantum electrodynamics
EIS: electrochemical impedance spectroscopy
DOS: density of states
SAM: self-assembled monolayer
SPR: surface plasmon resonance
HNSCC: head and neck squamous cell carcinoma
ssDNA: single-stranded DNA
dsDNA: double-stranded DNA
LOD: limit of detection
LOQ: limit of quantification
DFT: density functional theory
ITC: isothermal titration calorimetry
LDSS: low-dimensional scale structures
