# Supplementary material for: Separating Detection Performance from Thermodynamic Interpretability in Quantum-Capacitance Affinity Assays
Source: Langmuir. 2026 Sep 25;42(40):29736–50. doi: 10.1021/acs.langmuir.6c03092 (PMC13674245; doi:10.1021/acs.langmuir.6c03092)
Supplement: Supplementary file 1 [file la6c03092_si_001.pdf]

Supporting Information

Separating Detection Performance from Thermodynamic Interpretability in Quantum-Capacitance Affinity Assays

Olivia Carr[a] and Paulo Roberto Bueno[a,b,*]

[a]Institute of Chemistry, Department of Physics and Mathematics, São Paulo State University (UNESP), Araraquara, SP 14800-060, Brazil

[b]Centro Interdisciplinar de Química e Bioloxía (CICA) and Departamento de Química, Facultade de Ciencias, Universidade da Coruña, 15071 A Coruña, Galicia, Spain

*E-mail: paulo-roberto.bueno@unesp.br

Contents

S1 From the Langmuir Isotherm to Quantum Capacitance: Full Statistical Mechanics Derivation **S2**

S1.1 Phenomenological (Kinetic) Starting Point . . . S3

S1.2 Statistical Mechanics Derivation of the Langmuir Isotherm . . . S3

S1.3 Connection to the Quantum Energy e^2/C_q . . . S4

S1.4 Decomposition of C_q: Occupancy Channel vs. Formal Potential Channel S6

S1.5 Contributions of Each Channel to the Thermodynamic-Plot Slope . . . S8

S1.6 The Transduction Parameter χ and the Signal–Occupancy Relation . S11

S1.7 Relation to the Hill Isotherm and Its Extrapolation to Surfaces . . . S13

S2 DFT Connection: Chemical Hardness and the Quantum Capacitance S14

S3 Isoscopic Framework and Extraction of R_q **S14**

S3.1 The Quantum RC Circuit Model . . . S14

S3.2 Independent Extraction of R_q . . . S15

S3.3 Physical Meaning of the Isoscopic Invariance . . . S15

S3.4 Correspondence Between Classical and Quantum Electrochemistry, and the Role of the Two Degeneracies . . . S20

S4 Apparent Interfacial Constants by Non-Linear Least Squares S22

S4.1 The Hanes–Woolf linearisation . . . S22

S4.2 Non-linear least-squares procedure . . . S23

S4.3 Status of the logarithmic representation . . . S25

S4.4 What the logarithmic representation does not provide . . . S25

S5 Detection Performance versus Thermodynamic Interpretability S26

S6 Experimental Data Processing Algorithm S27

S6.1 Step 1: Import and Variable Conversion . . . S27

S6.2 Step 2: Determination of C_μ and C_q (Thermodynamic Parameter) . . S28

S6.3 Step 3: Determination of R_q (Kinetic Parameter) . . . S28

S6.4 Step 4: Affinity Matrix Consolidation S29

S7 Numerical Basis: Per-Concentration Data S29

S7.1 Robustness of the extraction S33

S8 Dispersion of the Electronic Relaxation: Langmuir as the Ideal Limit of Temkin S33

S8.1 The two limits S33

S8.2 Quantitative bridge between the domains S34

S8.3 α is invariant with analyte concentration S35

S8.4 R_q identifies the branch whose relaxation is being described S36

S8.5 Independent estimators of R_q S37

S8.6 The low-frequency response below the semicircle S39

S8.7 Isotherm comparison on the same data S40

S9 Scope and Limitations S41

S10 List of Symbols S44

S11 Expected Spectroscopic Signatures and Validation Panels S45

S11.1 Panel Descriptions and Expected Physical Behaviour S45

S11.2 Diagnostic Guide for Non-Ideal Behaviour S46

S12 Impedance Spectra of the Two Sensing Configurations S48

S12.1 Concentration ranges and spectral form S48

S12.2 Surface-modification controls and blank reproducibility S49

S12.3 Real admittance and the basis for R_q extraction S49

S1. From the Langmuir Isotherm to Quantum Capacitance: Full Statistical Mechanics Derivation

This section presents the derivation connecting the Helmholtz free energy of adsorption at a molecularly engineered electrode surface to the measurable quantum energy e^2/C_q, and sets out what that connection does and does not deliver. It gives the statistical-mechanical basis of the quantum-capacitance transducer and the relation between the transducer signal and fractional occupancy, from which the apparent interfacial constants are obtained. It does *not* give access to the standard Gibbs free energy of binding from the logarithmic representation: as shown in Section S1.3, the adsorption energy ε cancels from the adsorption-equilibrium condition by construction, so the intercept of the logarithmic representation carries no affinity information; ΔG° would follow from K_a only if χ were identified and equilibrium demonstrated.

S1.1. Phenomenological (Kinetic) Starting Point

The adsorption of a ligand (L) onto a molecular receptor (R) immobilised at an electrode surface is described by the dynamic equilibrium:

$$\mathrm{L} + R \rightleftharpoons \mathrm{LR} \tag{S1}$$

with association and dissociation rates:

$$v_a = k_{\mathrm{on}}[\mathrm{L}](1-\theta), \qquad v_d = k_{\mathrm{off}}\,\theta \tag{S2}$$

where $\theta = N_{\mathrm{b}}/N_t$ is the fractional site occupancy (N_{b} bound ligands over N_t total receptor sites; the symbol N is reserved throughout for the number of electrochemically accessible redox states, Section S1.6). At equilibrium ($v_a = v_d$):

$$\theta = \frac{K_a[\mathrm{L}]}{1 + K_a[\mathrm{L}]}, \qquad K_a = \frac{k_{\mathrm{on}}}{k_{\mathrm{off}}} \tag{S3}$$

This is the Langmuir isotherm derived from kinetics. The thermodynamic (statistical mechanics) origin of K_a is derived in the following subsection.

S1.2. Statistical Mechanics Derivation of the Langmuir Isotherm

Partition function. Consider a system of N_{b} identical ligands distributed over N_t equivalent and independent adsorption sites, each with microscopic interaction energy ε (defined, following ref. 1, as the energy *required* for the binding reaction between the ligand and the surface site, so that a larger ε corresponds to weaker binding).[1] The canonical partition function $Z(N)$ is:

$$Z(N_{\mathrm{b}}) = \binom{N_t}{N_{\mathrm{b}}} e^{-N_{\mathrm{b}}\varepsilon/k_{\mathrm{B}}T} = \frac{N_t!}{N_{\mathrm{b}}!\,(N_t - N_{\mathrm{b}})!}\, e^{-N_{\mathrm{b}}\varepsilon/k_{\mathrm{B}}T} \tag{S4}$$

Helmholtz free energy. The Helmholtz free energy is $A = -k_{\mathrm{B}}T \ln Z$:

$$A = -k_{\mathrm{B}}T \left[\ln(N_t!) - \ln(N_{\mathrm{b}}!) - \ln((N_t - N_{\mathrm{b}})!) - \frac{N_{\mathrm{b}}\varepsilon}{k_{\mathrm{B}}T}\right] \tag{S5}$$

Stirling approximation. In the thermodynamic limit of large particle numbers, $\ln(x!) \approx x \ln x - x$:

$$A \approx -k_{\mathrm{B}}T \left[N_t \ln N_t - N_{\mathrm{b}} \ln N_{\mathrm{b}} - (N_t - N_{\mathrm{b}}) \ln(N_t - N_{\mathrm{b}}) - \frac{N_{\mathrm{b}}\varepsilon}{k_{\mathrm{B}}T}\right] \tag{S6}$$

[1] ε so defined is a standard *free* energy of adsorption per site rather than a bare interaction energy, since it must absorb the configurational entropy of the adsorbed state in order to yield K_a. The opposite (lattice-gas) convention, in which $\varepsilon > 0$ denotes favourable binding, would instead require the Boltzmann factor $e^{+N_{\mathrm{b}}\varepsilon/k_{\mathrm{B}}T}$ in eq. S4.

Chemical potential of the surface. The surface chemical potential μ_s is the partial derivative of A with respect to N_b at constant T and N_t:

$$\mu_s = \left(\frac{\partial A}{\partial N_\mathrm{b}}\right)_{T,N_t} = -k_\mathrm{B}T\left[\ln\left(\frac{N_t - N_\mathrm{b}}{N_\mathrm{b}}\right) - \frac{\varepsilon}{k_\mathrm{B}T}\right] \tag{S7}$$

Expressing in terms of $\theta = N_\mathrm{b}/N_t$, so that $(N_t - N_\mathrm{b})/N_\mathrm{b} = (1-\theta)/\theta$:

$$\mu_s = -k_\mathrm{B}T\left[\ln\left(\frac{1-\theta}{\theta}\right) - \frac{\varepsilon}{k_\mathrm{B}T}\right] \tag{S8}$$

which can be rewritten as:

$$\mu_s = k_\mathrm{B}T\ln\left(\frac{\theta}{1-\theta}\right) + \varepsilon \tag{S9}$$

Note on the sign. μ_s increases with ε, because ε is defined here as the energy cost of adsorption; eqs. S8 and S9 follow directly from eq. S7 with this convention.

Equilibrium condition. At thermodynamic equilibrium, $\mu_s = \mu_L$, where the bulk ligand chemical potential is $\mu_L = \mu_L^\ominus + k_\mathrm{B}T\ln([\mathrm{L}]/[\mathrm{L}]_0)$:

$$k_\mathrm{B}T\ln\left(\frac{[\mathrm{L}]}{[\mathrm{L}]_0}\right) = -k_\mathrm{B}T\left[\ln\left(\frac{1-\theta}{\theta}\right) - \frac{\varepsilon}{k_\mathrm{B}T}\right] \tag{S10}$$

The sign of the ε term follows eq. S8, from which this equation is obtained by setting $\mu_s = \mu_L$, and is carried consistently through eqs. S11–S13 to $K_a = [\mathrm{L}]_0^{-1}\exp(-\varepsilon/k_\mathrm{B}T)$. Rearranging:

$$\ln\left(\frac{[\mathrm{L}]}{[\mathrm{L}]_0}\right) = \ln\left(\frac{\theta}{1-\theta}\right) + \frac{\varepsilon}{k_\mathrm{B}T} \tag{S11}$$

Exponentiating:

$$\frac{[\mathrm{L}]}{[\mathrm{L}]_0} = \frac{\theta}{1-\theta}\,e^{\varepsilon/k_\mathrm{B}T} \quad\Rightarrow\quad \frac{\theta}{1-\theta} = \frac{[\mathrm{L}]}{[\mathrm{L}]_0}\,e^{-\varepsilon/k_\mathrm{B}T} \tag{S12}$$

Solving for θ:

$$\theta = \frac{K_a[\mathrm{L}]}{1+K_a[\mathrm{L}]}, \qquad \text{where} \quad K_a = \frac{1}{[\mathrm{L}]_0}\,e^{-\varepsilon/k_\mathrm{B}T} \tag{S13}$$

This recovers *from first principles* the Langmuir isotherm (eq. S3), revealing the microscopic thermodynamic meaning of K_a: it encapsulates the adsorption energy ε and the reference concentration $[\mathrm{L}]_0$.

S1.3. Connection to the Quantum Energy e^2/C_q

Eq. S13 is a relation for the receptor sites: it gives their occupancy θ at equilibrium with the ligand in solution, and no capacitance enters it. The connection to the measured capacitance is made in four steps, each stating which capacitance applies — the capacitance per accessible state, $c_q = C_q/N$, or the ensemble capacitance of the electrode, C_q.

(i) Charging energy per accessible state. At the formal potential, where the Fermi–Dirac occupancy of the redox states is one half, $C_\mu = Ne^2/4k_\mathrm{B}T$ and, under the isoscopic condition, $C_q = g_e C_\mu = Ne^2/2k_\mathrm{B}T$ (Section S1.4, eq. S19). Per accessible state,

$$\frac{e^2}{c_q} = \frac{Ne^2}{C_q} = 2k_\mathrm{B}T, \tag{S14}$$

independent of the ligand concentration. The distinction between single-microstate and ensemble capacitance, $C_q = Nc_q$, is set out in ref. 2.

(ii) Ensemble capacitance. The measured quantity is the ensemble capacitance,

$$\frac{e^2}{C_q} = \frac{2k_\mathrm{B}T}{N}, \tag{S15}$$

so that, the energy per state being fixed, the analyte dependence of e^2/C_q can enter only through N.

(iii) Accessible-state count. Binding renders a fraction of the redox centres electrochemically inaccessible, $N = N_0(1 - \chi\theta)$ (Section S1.6, eq. S23).

(iv) Adsorption equilibrium. θ is given by eq. S13, obtained from $\mu_s(\theta) = \mu_L$ (eq. S10).

Combining (ii) and (iii),

$$\frac{e^2}{C_q}([\mathrm{L}]) = \frac{2k_\mathrm{B}T}{N_0}\frac{1}{1-\chi\theta([\mathrm{L}])}, \qquad \ln\frac{C_{q,0}}{C_q} = -\ln(1-\chi\theta) = \ln\left(1 + \frac{\mathrm{RR}(\%)}{100}\right), \tag{S16}$$

the last equality following from eq. S24. The ligand concentration therefore enters the measured capacitance only through θ.

The adsorption energy cancels by construction. K_a is defined through ε (eq. S13), so substituting the solution $\theta/(1-\theta) = K_a[\mathrm{L}]$ back into the equilibrium condition, eq. S10, returns $k_\mathrm{B}T\ln([\mathrm{L}]/[\mathrm{L}]_0)$ on both sides for any value of ε. The cancellation is an identity: it is a consequence of the parametrisation and carries no physical content. Its only practical corollary is that no plot of an energy against $\ln([\mathrm{L}]/[\mathrm{L}]_0)$ can return K_a from its intercept, so that neither K_a nor ΔG° is accessible from that representation; affinity information resides in the occupancy analysis of Section S1.6. Eq. S10 is dimensionally well posed, the argument of each logarithm being a ratio; all logarithms of concentration in this work are written in this form.

What the model predicts. The physical predictions of the framework are: (a) the form of the calibration, eq. S25, not borne out over the range sampled, where the response is log-linear (Section S8.7); (b) invariance of R_q when transduction is by occupancy of a fixed channel, satisfied in the unamplified configuration and violated in the amplified one (Section S3); (c) invariance of the formal potential across the titration, expected for this architecture but not verified directly (Section S1.4); and (d) a charge-relaxation resistance at $h/2e^2$, supported by the equivalent-circuit estimate and by the relaxation frequency predicted from the measured capacitance (Section S8.5).

Slope of the logarithmic representation. Differentiating eq. S16,

$$\frac{\mathrm{d}\ln(e^2/C_q)}{\mathrm{d}\ln[\mathrm{L}]} = \frac{\chi}{1-\chi\theta}\,\frac{\mathrm{d}\theta}{\mathrm{d}\ln[\mathrm{L}]}, \tag{S17}$$

which is dimensionless, depends on the interface through χ and on the isotherm through $\mathrm{d}\theta/\mathrm{d}\ln[\mathrm{L}]$, and is therefore interface-specific rather than universal. It is not used in this work to determine χ.

S1.4. Decomposition of C_q: Occupancy Channel vs. Formal Potential Channel

The electrochemical capacitance of a redox interface measured at fixed electrode potential V is given by the thermal broadening of the Fermi–Dirac occupancy function over the redox density of states:[3, 4]

$$C_\mu = \frac{e^2N}{k_\mathrm{B}T}\, f\!\left(\frac{e(V-V_F)}{k_\mathrm{B}T}\right)\left[1-f\!\left(\frac{e(V-V_F)}{k_\mathrm{B}T}\right)\right], \qquad C_q = g_eC_\mu = \frac{2e^2N}{k_\mathrm{B}T}\, f(1-f) \tag{S18}$$

where N is the total number of accessible redox states, V_F is the formal potential of the redox couple, k_B is the Boltzmann constant, T is the absolute temperature, and $f(x) = (1+e^x)^{-1}$ is the Fermi–Dirac function. This expression makes explicit that the measured C_μ depends on two physically distinct quantities: the number of states N and the formal potential V_F.

Note on the assignment. The Fermi–Dirac expression above is written for C_μ rather than for C_q. This assignment matters: writing it for C_q would give $C_q = Ne^2/4k_\mathrm{B}T$ at half-occupancy, inconsistent by a factor of $g_e = 2$ with the rest of this formalism. The expression properly describes the *directly measured* capacitance C_μ — the quantity conjugate to the relaxation time $\tau = R_qC_\mu$ (Section S3) — so that at $f = 1/2$

$$C_\mu = \frac{Ne^2}{4k_\mathrm{B}T}, \qquad C_q = g_eC_\mu = \frac{Ne^2}{2k_\mathrm{B}T}, \qquad N = \frac{4k_\mathrm{B}TC_\mu}{e^2} = \frac{2k_\mathrm{B}TC_q}{e^2}. \tag{S19}$$

The extraction of R_q is unaffected: $\tau = R_qC_\mu$ and $\Sigma = R_qC_\mu^2$ were already correct as written, the equivalent form in terms of the inferred quantum capacitance being $\Sigma = R_qC_q^2/4$ (Section S3); the factor of four belongs to the C_q form and not to the C_μ form. Note also that N is obtained from the *absolute* measured capacitance in farads; no electroactive-area normalisation enters. Conversion of N to a surface coverage $\Gamma = N/A$ requires both the electroactive area and the assumption of single equivalent states, and is not used in this work.

Upon ligand binding, the total differential of C_q at fixed V is:

$$\mathrm{d}C_q = \underbrace{\frac{\partial C_q}{\partial N}\,\mathrm{d}N}_{\text{occupancy channel}} + \underbrace{\frac{\partial C_q}{\partial V_F}\,\mathrm{d}V_F}_{\text{formal potential channel}} \tag{S20}$$

These two channels correspond to physically distinct molecular mechanisms:

Occupancy channel ($\partial C_q/\partial N$). A change in the number of electrochemically accessible redox states at constant V_F. Molecular recognition at a spatially separated receptor site

perturbs the local chemical potential μ_s of the monolayer, altering the occupation of the redox DOS without shifting its energy position. This was demonstrated for protein recognition (CRP, α-synuclein, PAP) at antibody-modified ferrocene SAMs, where the Fermi energy of the junction was shown to remain constant across the analyte titration while the DOS peak height changed systematically.[4] The occupancy relation of eq. S16 is derived entirely within this channel: $\Delta C_q \propto \Delta N \propto \Delta\theta$.

Formal potential channel ($\partial C_q/\partial V_F$). A shift in the redox formal potential V_F at constant N. When the analyte is electronically coupled to the redox centre through direct or through-bond communication, binding stabilises or destabilises one oxidation state relative to the other, shifting V_F. In the QR framework, such a shift corresponds to displacing $E_\mu = E_F - eV$ away from zero at the measurement potential $V = V_F$, which perturbs the Fermi–Dirac occupancy $f = [1 + \exp(E_\mu/k_BT)]^{-1}$ away from its isoscopic equilibrium value of $f = 1/2$. When $f \neq 1/2$, the occupancy of reduced states (f) and oxidised states ($1 - f$) become unequal, and any K_a extracted from the Langmuir analysis of ΔC_q in this channel reflects this imbalance rather than the true equilibrium thermodynamic constant. This is the primary transduction mechanism in voltammetric anion sensors reviewed by Hein, Beer, and Davis,[5] where the cathodic perturbation $\Delta E_{1/2} = \Delta V_F$ upon anion binding is the analytical signal; the redox-state-dependent apparent affinity extracted in those systems corresponds in the QR language to the asymmetry between f and $(1 - f)$ at the perturbed operating point.

Suppression of the formal potential channel at the DOS maximum. When the measurement is conducted at $V = V_F$ (the DOS peak), the formal potential channel is suppressed to second order. At the peak, $f(1 - f) = 1/4$ is at its maximum, and by symmetry:

$$\left.\frac{\partial C_q}{\partial V_F}\right|_{V=V_F} = \frac{e^2 N}{k_B T}\frac{\partial}{\partial V_F}\left[f\left(\frac{e(V - V_F)}{k_B T}\right)(1 - f)\right]_{V=V_F} = 0 \tag{S21}$$

because $f(1 - f)$ evaluated at $V = V_F$ is at a stationary point with respect to V_F. Consequently, for $|\Delta V_F| \ll k_BT/e \approx 25.7$ mV, the contribution of the formal potential channel to the measured ΔC_q is negligible to first order, and the occupancy relation remains valid. For larger shifts $|\Delta V_F| \gtrsim k_BT/e$ — as routinely observed in voltammetric anion sensing systems where ΔV_F ranges from tens to hundreds of mV — the formal potential channel dominates and the occupancy-based relation no longer applies.

Experimental validation. In the present HNSCC genosensing system, the formal potential stability condition $\partial V_F/\partial[\mathrm{L}] = 0$ is expected on structural grounds rather than established by a separate CV titration experiment. On this basis analyte binding is expected to modulate the occupancy of the DOS (the number of accessible redox states N, i.e., the occupancy channel) without displacing the formal potential V_F. Direct experimental evidence for this behaviour in equivalent ferrocene SAM architectures was provided by Bueno et al.,[4] who demonstrated that E_F/e remains constant across the entire analyte titration for protein recognition (CRP, α-synuclein, PAP) at antibody-modified ferrocene SAMs, while only the DOS peak height changes systematically (see Figure 2b of that reference). The cyclic voltammograms shown in Figure S1 of the present SI confirm the well-defined, symmetric ferrocene redox couple at $E_{F/e} = 0.367 \pm 0.008$ V vs. Ag|AgCl and its positional stability between the unamplified and amplified interface configurations, consistent with this structural decoupling. The joint satisfaction of the R_q invariance condition (eq. S44, kinetic decou-

pling) and the formal potential stability condition (supported structurally and by literature precedent; electronic decoupling) constitutes the set of physical prerequisites under which the measured response is *consistent with* thermodynamic transduction. We emphasise that formal-potential stability is supported here indirectly, by molecular architecture and literature precedent, rather than by direct CV titration across the DNA concentration series, and the interpretation is qualified accordingly (Section S9).

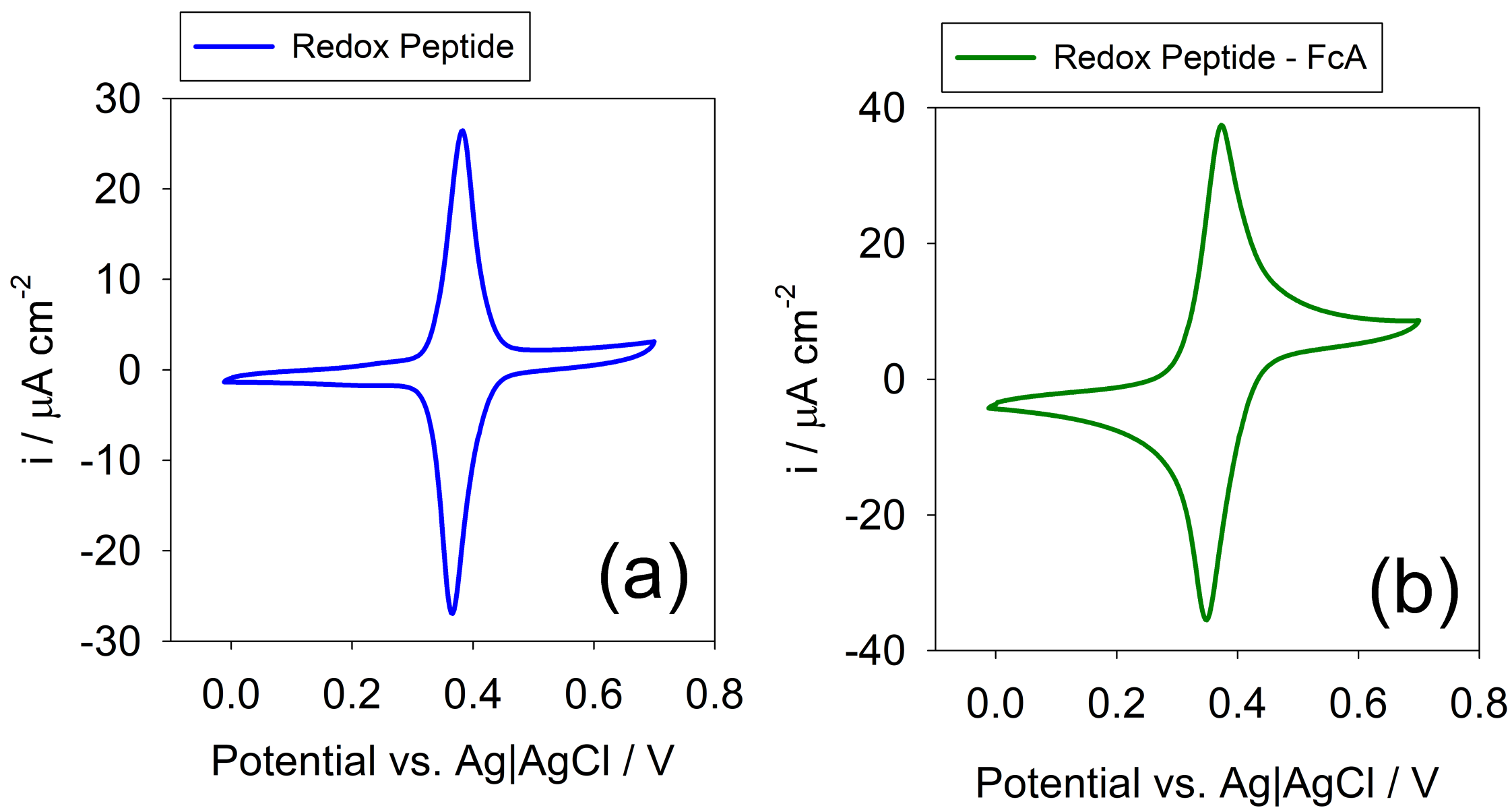

Figure S1: Cyclic voltammograms of the redox-tagged peptide monolayer interfaces confirming successful surface modification and the redox activity of both sensing configurations at the blank (analyte-free) interface. (a) Unamplified configuration (Redox Peptide, blue): current density i (μA cm^{-2}) vs. potential, showing a well-defined, symmetric ferrocene redox couple at $E_{F/e} = 0.367 \pm 0.008$ V vs. Ag|AgCl, confirming successful monolayer assembly and the characteristic formal potential of the Pep-Fc interface. (b) Amplified configuration (Redox Peptide + FcA, green): the somewhat larger peak current densities, also in μA cm^{-2}, reflect the additional redox contribution of 100 μM FcA in solution, whose formal potential is aligned with the interfacial Pep-Fc states to achieve quantum-rate amplification. These voltammograms characterise the blank interface configurations and confirm the well-defined, symmetric redox response in each case. The invariance of $E_{F/e}$ upon analyte binding in equivalent ferrocene SAM architectures has been demonstrated directly by Bueno et al. (see Figure 2b of ref. 4), supporting the structural argument that the peptide backbone suppresses through-bond electronic coupling between the DNA hybridisation event and the ferrocene redox orbital. Scan rate: 100 mV s^{-1}; potential window 0.0–0.7 V, as reported in ref. 6. Data from ref. 6.

S1.5. Contributions of Each Channel to the Thermodynamic-Plot Slope

The occupancy and formal-potential channels contribute differently to the slope of the thermodynamic plot, and it is useful to see why neither yields a universal value.

Occupancy channel. In the pure occupancy channel ($\Delta V_F = 0$, $\Delta N \neq 0$), $e^2/C_q =$

$2k_BT/N$ at $V = V_F$. Per accessible state this gives $e^2/c_q = Ne^2/C_q = 2k_BT$ exactly, independent of K_a, N_0, receptor type or surface chemistry (eq. S14). The analyte dependence of the ensemble quantity enters through N alone, and the slope of the logarithmic representation is given by eq. S17, which depends on χ and on the isotherm and is therefore interface-specific rather than universal.

Formal-potential channel. In the pure formal-potential channel ($\Delta N = 0$, $\Delta V_F \neq 0$), with measurement fixed at the original $V_{F,0}$:

$$\frac{\partial(e^2/C_q)}{\partial \ln[\mathrm{L}]} = \frac{\partial(e^2/C_q)}{\partial V_F} \cdot \frac{\partial V_F}{\partial \ln[\mathrm{L}]} \tag{S22}$$

The first factor depends on how far V_F has shifted relative to k_BT/e through the nonlinear Fermi–Dirac derivative. The second is proportional to the shift in equilibrium occupancy: a change in V_F at fixed measurement potential moves $E_\mu = E_F - eV$ away from zero, perturbing f away from 1/2; in the fast-exchange voltammetric limit the magnitude scales as $\Delta f \propto (k_BT/e)\Delta V_F/4$ for small shifts,[5] which varies widely between systems. The resulting slope is receptor- and analyte-specific.

Consequence. Neither channel delivers a slope that can serve as a system-independent standard. The per-state identity $e^2/c_q = 2k_BT$ does hold universally, but it is not what the thermodynamic plot displays. Discrimination between the two channels is therefore based on V_F stability (Section S1.4) and on R_q invariance (Section S3), not on the slope.

Implications for the validation hierarchy. Two experimental tests check the consistency of the occupancy interpretation; they address distinct failure modes and are non-redundant. A third observable, the dispersion exponent α, is reported alongside them but does not identify the adsorption regime (Section S8.3). The slope of the thermodynamic plot is *not* part of this hierarchy, for the reason given above (Table S1).

Table S1: Internal consistency checks for the quantum-capacitance framework. The first two are consistency criteria and must both pass for the response to be read as occupancy-dominated; neither excludes non-specific blocking of accessible centres, for which a non-complementary control is the relevant test. The dispersion exponent does not validate the response and does not by itself identify the adsorption regime. The slope of the thermodynamic plot is not a criterion.

**Test**	**What it supports**	**What it misses**	**Method**
R_q invariance ($\partial R_q/\partial \ln[\mathrm{L}] = 0$)	No detectable change of the relaxation resistance, hence of the tunnelling pathway (below about 5% here)	Formal potential shifts; adsorption regime; non-specific blocking of accessible centres; changes below about 5%	EIS (ω^2 slope)
V_F stability ($\partial V_F/\partial[\mathrm{L}] = 0$)	Electronic decoupling: redox orbital energy unaffected by binding	Kinetic artefacts; adsorption regime	CV across titration (general method); not verified directly in the present system (see ref. 4 for experimental precedent)
Dispersion exponent α (reported, not a criterion)	Width of the distribution of electronic relaxation times; invariance with concentration indicates a static distribution	Origin of the dispersion (electrode heterogeneity, tunnelling distances, distributed capacitive behaviour, or site energies); does not identify the adsorption regime	Cole–Cole fit of the faradaic $C^*(\omega)$

The practical recommendation is therefore: (1) confirm R_q invariance from the low-frequency $\mathrm{Re}[G^*(\omega)]$ data, which establishes that binding does not perturb the tunnelling pathway; (2) confirm V_F stability, either by CV across the titration when feasible, or by verifying that the molecular architecture suppresses through-bond electronic coupling between analyte and redox centre — as demonstrated for antibody-modified ferrocene SAMs by Bueno *et al.*[4]; and (3) compare functional forms on the calibration data (Section S8.7) and test the equilibrium premise directly, since the constant returned by a fit is an equilibrium binding constant only at equilibrium and only if χ is identifiable. In the present data neither condition is established, and the constants are reported as calibration parameters in consequence.

When (1) and (2) pass, the response is consistent with occupancy-dominated transduction; (3) determines whether the fitted constant can be read as a binding constant at all. A failed R_q test points to kinetic or structural contributions; a failed V_F test to formal-potential contamination. The slope of the thermodynamic plot is not part of this sequence: as shown in Section S1.5, the universal identity is the per-state relation $e^2/c_q = 2k_\mathrm{B}T$, while the ensemble slope is interface-specific.

S1.6. The Transduction Parameter χ and the Signal–Occupancy Relation

What is, and is not, proportional to occupancy. The transducer signal is $S = 1/C_q$ because it is proportional to the electrochemical energy $e^2/C_q = E_\mu/g_e$, E_μ being the change in electrochemical potential on charging the accessible states within the quantum-rate framework. The physical quantity responding to binding is the electronic occupancy N, which is linear in θ by eq. S23; since $C_q = 2k_\mathrm{B}TN/e^2$, the fractional change in capacitance is therefore strictly proportional to occupancy, $(C_{q,0} - C_q)/C_{q,0} = \chi\theta$. What is *not* proportional to θ is either C_q or $1/C_q$ taken alone, since both carry the blank offset: binding renders centres inaccessible, so C_q decreases with occupancy; and $1/C_q \propto 1 + K_a[\mathrm{L}] = (1-\theta)^{-1}$, which is not proportional to θ either. The correct relation is derived below.

The physical starting point. Since $C_q \propto e^2\,\mathrm{DOS}$ and ligand binding renders a fraction of the redox centres *electrochemically inaccessible*, the number of accessible states decreases with occupancy. The ferrocene centres are covalently tethered and are neither destroyed nor chemically altered by hybridisation; their number is fixed by the monolayer. What hybridisation removes is their *accessible occupancy*: a fraction of the centres can no longer exchange charge with the electrode within the measurement, and N counts only those that still can. A fall in N is therefore a loss of addressable occupancy of a fixed population of states, distinct from the Fermi–Dirac occupancy f, which stays at 1/2 throughout because the measurement is made at V_F and the formal potential does not shift (Section S1.4). Writing this to first order,

$$N([\mathrm{L}]) = N_0\,(1 - \chi\theta)\,, \tag{S23}$$

where N_0 is the number of accessible states at the blank interface and $\chi \in (0, 1]$ is the *transduction parameter* — the constant of proportionality relating occupancy to the fraction of redox centres rendered electrochemically inaccessible. The symbol χ is used because β denotes $1/k_\mathrm{B}T$ throughout the quantum-rate formalism. Operationally, $\chi \to 0$ describes an interface whose accessible-state count is barely perturbed by binding, and $\chi \to 1$ one in which essentially all accessible states are affected. We use χ as a phenomenological parameter of the signal–occupancy relation and make no claim as to its microscopic origin; distinguishing a geometric from a kinetic contribution would require systems in which the two are varied independently, which the present data do not provide.

The transducer signal. With $C_q \propto N$ and the transducer signal defined, as in the main text, as $S = 1/C_q$, the relative response is

$$\frac{\mathrm{RR}(\%)}{100} = \frac{S_i - S_0}{S_0} = \frac{C_{q,0}}{C_{q,i}} - 1 = \frac{N_0}{N_i} - 1 = \frac{\chi\theta}{1 - \chi\theta}. \tag{S24}$$

Substituting the Langmuir isotherm $\theta = K_a[\mathrm{L}]/(1 + K_a[\mathrm{L}])$ gives the central working relation of this work:

$$\boxed{\frac{\mathrm{RR}(\%)}{100} = \frac{\chi\,K_a[\mathrm{L}]}{1 + (1-\chi)\,K_a[\mathrm{L}]}} \tag{S25}$$

Consequences. Three points follow, of which the second bears directly on the interpretation of the fitted constants.

1. Eq. S25 is a Langmuir hyperbola in [L]. It is the form expected for an equilibrium occupancy response, and it justifies *a posteriori* the use of RR(%), and not C_q or $1/C_q$, as the Langmuir observable. The transduction variable is now the same quantity in the theory, the figures and the tables.

2. The apparent affinity returned by any isotherm analysis of RR(%) is

$$K_a^{\mathrm{app}} = (1-\chi)\, K_a, \tag{S26}$$

so that K_a can be recovered from K_a^{app} only if χ is identifiable, $\chi \ll 1$ being the special case in which no correction is needed. This condition is stated explicitly among the requirements for thermodynamic interpretation (Section S9).

3. If the titration reaches saturation, then $\theta \to 1$ and the plateau of the calibration curve gives the transduction parameter directly, as $\mathrm{RR}(\%)_{\max} = 100\chi/(1-\chi)$. The present titration does not saturate, so only the product $\chi\theta$ is accessible from the response; since $\theta \leq 1$ this bounds χ from below, and it is that bound which is used in this work (see below).

Why the thermodynamic plot does not have slope $k_{\mathrm{B}}T$. The per-state identity, eq. S14, is independent of the ligand concentration: the charging energy of each accessible state is fixed at $2k_{\mathrm{B}}T$. The logarithmic representation is constructed from the ensemble capacitance, whose analyte dependence enters only through N (eq. S16), and its slope is given by eq. S17. That slope depends on χ and on $\mathrm{d}\theta/\mathrm{d}\ln[\mathrm{L}]$, and so is interface-specific; there is no reason for it to equal $k_{\mathrm{B}}T$ or any other universal value.

Numerical basis. Regressing $\ln(e^2/C_q)$ against $\ln[\mathrm{L}]$ over the six concentrations 10^3–10^8 aM of Table S4 gives a relative slope of 1.161×10^{-2} per natural-logarithm unit ($R^2 = 0.992$). The row at 10^8 aM is retained: after exclusion of the single non-convergent spectrum (electrode 247, Section S7) it carries a 9% relative standard deviation and lies in sequence with the remaining rows. The slope is reported as a description of the data and is not used to determine χ.

The determination of $\chi\theta$, and the bound on χ. From eq. S24, $\chi\theta = \mathrm{RR}(\%)/(100+\mathrm{RR}(\%))$, which as a number is the measured fractional change of capacitance $1 - C_\mu/C_{\mu,0}$; its reading as a product of transduction parameter and occupancy requires eq. S23. Its maximum, computed from the capacitances of Table S4, is 0.169 (unamplified) and 0.625 (amplified). Since $\theta \leq 1$ — for the Langmuir isotherm and equally for the full Temkin expression, eq. S55 —

$$\chi \;\geq\; (\chi\theta)_{\max}, \tag{S27}$$

giving $\chi \geq 0.169$ and $\chi \geq 0.625$, so that $K_a = K_a^{\mathrm{app}}/(1-\chi)$ exceeds the apparent constant by factors of at least 1.20 and 2.67. On the published calibration of ref. 6, to which the constants of Table 2 of the main text are fitted, the bounds are $\chi \geq 0.131$ and ≥ 0.524, giving factors of at least 1.15 and 2.10. χ itself is not identifiable from these data: the fit

does not determine it, because the plateau is not sampled (Section S4.2), and no independent measurement supplies it.

A note on what is not *an independent check.* The relative slope may be computed from the capacitances of Table S4 or read from the plotted ordinate of Figure 5 of the main text, but the two are not independent estimates and their agreement is not corroboration: Figure 5 is constructed *from* Table S4, so the two routes are the same route.

Why no Langmuir isotherm reproduces the observed shape. The argument here does not turn on a particular affinity constant, and would be circular if it did. For a Langmuir isotherm $\mathrm{d}\ln\theta/\mathrm{d}\ln[\mathrm{L}] = 1-\theta$, so a response rising by a factor 3.5 uniformly over the 11.5 natural-logarithm units sampled requires $1-\theta \simeq 0.11$ to remain constant across five decades. No Langmuir isotherm has this property at any K_a: a small constant gives a rise of some three orders of magnitude over the range, and a large one saturates within the first two decades. This is a statement about curvature and is independent of the fitted parameters. The formal expression, for reference, is $\mathrm{d}(e^2/C_q)/\mathrm{d}\ln[\mathrm{L}] = E_0\chi\theta(1-\theta)/(1-\chi\theta)^2$, which is small at both extremes of occupancy. Evaluating it requires an affinity constant to be supplied, and the outcome follows from that choice; the comparison is therefore not offered as evidence for a regime assignment, and the curvature argument above — which requires no fitted parameter — is what carries the conclusion.

S1.7. Relation to the Hill Isotherm and Its Extrapolation to Surfaces

The Langmuir isotherm (eq. S13) is mathematically equivalent to the Hill equation with Hill coefficient $n = 1$, in the sense that both reduce to the same hyperbolic binding curve. The two are nevertheless physically distinct in origin. Langmuir was derived for *independent, equivalent adsorption sites* at a surface with no cooperativity;[7] Hill was formulated to describe cooperative ligand binding to multiple interdependent sites in *homogeneous bulk solution.*[8]

The distinction has a practical consequence for the platforms considered here. Because the Hill isotherm was conceived for solution-phase equilibria, its extrapolation to surface-immobilised receptor platforms — such as those used in high-throughput screening — introduces ambiguities that do not arise within the Langmuir framework. Isothermal titration calorimetry, which operates at micromolar-to-nanomolar bulk concentrations, is inherently susceptible to the cooperative multi-site effects the Hill model describes, particularly for targets with multiple binding domains or allosteric responses. The quantum-capacitance measurement, by contrast, is made at attomolar-to-femtomolar concentrations at a surface, where multi-occupancy of an individual receptor site is not expected.

This comparison concerns the *form* of the isotherm and not the status of the constant it returns. As Section S4.2 sets out, the constants obtained from the present data are reported as calibration parameters, and the question of which isotherm best describes the response is separate from the question — unresolved here — of whether the response is an equilibrium occupancy curve at all.

Reference concentration. Every logarithm of concentration in this document is written as $\ln([\mathrm{L}]/[\mathrm{L}]_0)$ with $[\mathrm{L}]_0 = 1$ M, the standard-state reference; the same convention is used in the main text.

S2. DFT Connection: Chemical Hardness and the Quantum Capacitance

In density functional theory (DFT), the electronic chemical potential of a system is defined as:

$$\mu = \left(\frac{\partial E_{\text{tot}}}{\partial N}\right)_{v(\mathbf{r})} \tag{S28}$$

where N is the total number of electrons and $v(\mathbf{r})$ is the external potential. The *chemical hardness* η is the second derivative:

$$\eta = \frac{1}{2}\left(\frac{\partial^2 E_{\text{tot}}}{\partial N^2}\right)_{v(\mathbf{r})} = \frac{1}{2}\frac{\partial \mu}{\partial N} \tag{S29}$$

Connecting to the quantum capacitance (main text, $C_q = e^2\, dn/dE$):

$$\frac{1}{C_q} = \frac{1}{e^2}\frac{\partial \mu}{\partial N} = \frac{2\eta}{e^2} \tag{S30}$$

The measurement of $1/C_q$ as a function of analyte concentration is therefore a spectroscopic measurement of the *variation in chemical hardness* of the interface upon molecular recognition. A harder interface (higher η, higher $1/C_q$) following ligand binding indicates stabilisation of the ligand–receptor complex LR, consistent with the principle of maximum hardness.

This connection is particularly significant for the interpretation of the DNA hybridisation results: formation of the dsDNA duplex at the interface increases the chemical hardness of the redox monolayer (reduces the electrochemical softness), leading to the observed decrease in C_q and increase in e^2/C_q.

S3. Isoscopic Framework and Extraction of R_q

S3.1. The Quantum RC Circuit Model

The electrode–monolayer–electrolyte interface within the quantum-rate framework is described by a quantum RC circuit in which the complex admittance is:

$$G(\omega) = \frac{j\omega C_\mu}{1 + j\omega\tau}, \qquad \tau = R_q C_\mu, \qquad C_\mu = \frac{C_q}{g_e} = \frac{C_q}{2} \tag{S31}$$

where C_μ is the electrochemical capacitance directly measured in EIS (the Nyquist semicircle diameter), $\tau = R_q C_\mu$ is the quantum relaxation timescale, and $C_q = g_e C_\mu = 2C_\mu$ is the quantum capacitance inferred from C_μ under the isoscopic condition $C_e = C_q$.[2]

The complex capacitance follows:

$$C^*(\omega) = \frac{G(\omega)}{j\omega} = \frac{C_\mu}{1 + j\omega\tau} \approx C_\mu(1 - j\omega\tau) + \mathcal{O}(\omega^2), \qquad \tau = R_q C_\mu \tag{S32}$$

In the low-frequency limit ($\omega\tau \ll 1$):

$$C^*(\omega \to 0) \approx C_\mu \tag{S33}$$

so that C_μ is obtained directly from the plateau of $C'(\omega)$ at low frequencies, corresponding to the diameter of the Nyquist capacitive semicircle. The quantum capacitance is then $C_q = g_e C_\mu = 2C_\mu$.

S3.2. Independent Extraction of R_q

The real part of the admittance, in terms of the directly measured C_μ:

$$\mathrm{Re}[G(\omega)] = \frac{\omega^2 R_q C_\mu^2}{1+(\omega\tau)^2} = \frac{\omega^2 R_q C_q^2/4}{1+(\omega\tau)^2} \approx R_q C_\mu^2 \omega^2 \quad (\omega\tau \ll 1) \tag{S34}$$

A plot of $\mathrm{Re}[G^*(\omega)]$ vs. ω^2 at low frequencies yields a straight line through the origin with slope $\Sigma = R_q C_\mu^2$ (equivalently $\Sigma = R_q C_q^2/4$ when expressed in terms of $C_q = 2C_\mu$). Since C_μ has been independently determined from $C^*(\omega)$ as the Nyquist semicircle diameter, R_q is extracted unambiguously:

$$R_q = \frac{\Sigma}{C_\mu^2} = \frac{4\Sigma}{C_q^2} \tag{S35}$$

The extraction formula is $R_q = \Sigma/C_\mu^2 = 4\Sigma/C_q^2$, where Σ is the slope of $\mathrm{Re}[G^*(\omega)]$ versus ω^2. *The bookkeeping of the factor $g_e^2 = 4$ requires care.* Since $\Sigma = C_\mu^2 R_q$ and $C_\mu = C_q/g_e$, the three forms $R_q = \Sigma/C_\mu^2 = g_e^2\Sigma/C_q^2 = 4\Sigma/C_q^2$ are identical. The factor 4 is purely the conversion between C_μ, which controls the slope, and C_q, which is inferred from it. Mis-substitution errs in opposite directions: Σ/C_q^2 underestimates R_q by g_e^2, while $4\Sigma/C_\mu^2$ overestimates it by the same factor.

Which quantity is measured. Throughout this work the $\omega \to 0$ plateau of the complex-capacitance plane — the diameter of the capacitive semicircle — is identified with the *measured* electrochemical capacitance C_μ (eq. S33), and the quantum capacitance is *inferred* from it as $C_q = g_e C_\mu = 2C_\mu$, with $N = 2k_\mathrm{B}TC_q/e^2 = 4k_\mathrm{B}TC_\mu/e^2$. This is the convention of the present work throughout; note that ref. 6 denotes the measured plateau itself C_q, so its tabulated capacitances correspond to C_μ here. The capacitances listed in Table S4 are the measured plateau itself, that is C_μ; the quantum capacitance is twice them. The factor $g_e^2 = 4$ enters the R_q extraction because the Büttiker expansion is written for C_q while the slope is governed by the experimentally accessible C_μ. This two-step extraction (first C_μ from C^* as the semicircle diameter, then R_q from $\mathrm{Re}[G^*]$ using C_μ) ensures parametric independence: the two parameters are not obtained from a simultaneous fit but from distinct spectroscopic observables.

S3.3. Physical Meaning of the Isoscopic Invariance

Before discussing the isoscopic invariance condition, it is important to clarify the relationship between the measured electrochemical capacitance C_μ, the quantum capacitance C_q, and the electrochemical energy E_μ.

The isoscopic condition and $C_q = 2C_\mu$. The electrochemical capacitance of the interface is the series combination of the classical ionic capacitance C_e and the quantum capacitance C_q:

$$\frac{1}{C_\mu} = \frac{1}{C_e} + \frac{1}{C_q} \tag{S36}$$

The isoscopic condition is the equality $C_e = C_q$. It is not an assumption imposed on the system but an attractor: minimisation of the Grand Canonical Potential ($\Delta\Omega = 0$) drives the electrolyte to reorganise until the ionic and quantum charging energies coincide, as demonstrated from first principles by Vignotto *et al.*[2, 3, 9] Its experimental signature is the convergence of the charge-relaxation resistance to $h/2e^2$, and its practical consequence is that the measured capacitance becomes proportional to the density of states alone. Under this condition eq. S36 simplifies to:

$$C_\mu = \frac{C_q}{g_e} = \frac{C_q}{2} \tag{S37}$$

where $g_e = 2$ is the isoscopic degeneracy arising because the quantum and ionic charging modes are energetically indistinguishable at the isoscopic attractor. The total electrochemical energy is:

$$E_\mu = \frac{e^2}{C_\mu} = g_e \frac{e^2}{C_q} = \frac{2e^2}{C_q} \tag{S38}$$

The role of E_μ and the Fermi–Dirac occupancy. The electrochemical energy E_μ is defined relative to the formal potential: $E_\mu = E_F - eV$, where $E_F = eV_F$ is the Fermi energy corresponding to the redox formal potential. At the measurement condition $V = V_F$, we have $E_\mu = 0$. The Fermi–Dirac occupancy of the redox state is:

$$f = \frac{1}{1 + \exp(E_\mu / k_\mathrm{B} T)} \tag{S39}$$

from which:

$$E_\mu = k_\mathrm{B} T \ln\left(\frac{1-f}{f}\right) \tag{S40}$$

At $V = V_F$, $E_\mu = 0$ and hence $f = 1/2$: the redox state is equally populated between reduced ($f = 1/2$) and oxidised ($1-f = 1/2$) forms. This is the DOS maximum (eq. S18 is maximised at $f(1-f) = 1/4$ when $f = 1/2$), and it is the condition at which the formal potential channel is suppressed (Section S1.4), the isoscopic condition $C_e \approx C_q$ is self-consistently maintained, and R_q is locked at $h/2e^2$. Any deviation of V from V_F — or equivalently any binding-induced shift ΔV_F that displaces the equilibrium point — perturbs E_μ away from zero and f away from 1/2, activating the formal potential channel. The $K_\mathrm{Ox}/K_\mathrm{Red}$ language used in the voltammetric anion sensing literature corresponds, in QR terms, to the asymmetry between f and $(1-f)$ at the perturbed operating point where $E_\mu \neq 0$.

Isoscopic invariance and the extraction factor. Under the isoscopic condition ($C_\mu = C_q/2$), the low-frequency expansion of the real admittance (eq. S34) in terms of the directly

measured C_μ is:

$$\mathrm{Re}[G^*(\omega)] \approx R_q C_\mu^2 \omega^2 = \frac{C_q^2}{4} R_q \omega^2 \tag{S41}$$

so that the extraction formula used throughout is $R_q = 4\Sigma/C_q^2$, where Σ is the slope of $\mathrm{Re}[G^*(\omega)]$ vs. ω^2. The factor of 4 in the C_q-based expression (from $g_e^2 = 4$) is the experimental hallmark of the isoscopic condition; without correctly accounting for the C_μ/C_q relationship, the extracted R_q would be displaced by a factor of $g_e^2 = 4$ from the quantum limit.[2]

The quantum relaxation resistance is physically a series combination:

$$R_q = 2\pi\,(R_{qt} + R_s) \tag{S42}$$

where R_s is the series resistance, encompassing the electrolyte and contact contributions, and R_{qt} the relaxation resistance of the faradaic branch. Neither term alone is the charge-transfer resistance: it is their sum, R_q, that corresponds to what is classically termed R_{ct}.[9] The factor 2π converts the circuit time constant $\tau = R_q C_\mu$ into the relaxation frequency f_r at which $\omega_r \tau = 1$, with $\omega_r = 2\pi f_r$; it follows from reading the relaxation in the frequency rather than the angular-frequency domain and is not an additional physical factor. *This relaxation frequency, which sits at the apex of the capacitive semicircle, should not be confused with the low-frequency equilibrium regime $\omega \to 0$ used to extract Σ in Section S3.2: the two are different parts of the same spectrum, and only the latter satisfies $\omega\tau \ll 1$.* Within the Landauer–Büttiker formalism,[2, 3, 9] R_{qt} is what sets the electronic coupling:

$$R_{qt} = \frac{h}{g_s e^2 \kappa N} \tag{S43}$$

where $g_s = 2$ is the spin degeneracy, N is the total number of accessible quantum channels, and $\kappa = \exp(-\gamma L)$ is the tunnelling transmission coefficient with decay constant γ and tunnelling length L. It is essential to emphasise that in the electrochemical context the tunnelling is *coherent but sub-unity*: $\kappa < 1$ in general, and the observation of $R_q \approx h/2e^2$ at isoscopic equilibrium does *not* require $\kappa = 1$. Rather, $R_q = 2\pi(R_{qt} + R_s)$ reaches the quantum resistance limit as an emergent property of Grand Canonical Potential minimisation ($\Delta\Omega = 0$),[2] through which the electrolyte self-organises to establish $C_e = C_q$ and thereby saturates the Heisenberg bound, $\Delta E \cdot \Delta t = e^2 R_q = h/2$ for the coherent channel. The identification of R_{qt} with the electronic coupling is what makes the invariance test meaningful. In the quantum-rate framework R_{qt} is not a phenomenological kinetic parameter but the inverse of a conductance, $G = 1/R_{qt} = \kappa G_0 N$, so that a change in R_{qt} reports directly on a change in the electronic coupling κ or in the number of channels N; the tunnelling decay constant γ of eq. S43 can be recovered from it directly.[9] Binding that altered the tunnelling pathway — by reorganising the film, by bringing the target into electronic contact with the redox centre, or by shifting the energy alignment — would change κ and therefore R_q. Binding that merely occupies receptor sites would not. The structural parameters γ, L, and N — and hence κ and R_{qt} — are governed by the film geometry and molecular orbital overlap, which are properties of the monolayer independent of the thermodynamic site occupancy θ. The isoscopic prediction is therefore:

$$\frac{\partial R_q}{\partial \ln[\mathrm{L}]} = 0 \tag{S44}$$

Violation of eq. S44 would indicate:

- Non-specific adsorption changing the monolayer thickness;
- Structural reorganisation of the SAM upon analyte binding;
- Artefacts from diffusive Faradaic current contributions not properly separated.
- Modification of the effective transmission probability $T_n(\mu)$ by a signal amplification mechanism — for example, the FcA-mediated electron transmittance pathway described in ref. [10], in which alignment of free-redox DOS states with the interfacial C_q states opens additional quantum channels whose coupling to the interface evolves with analyte concentration.

Invariance of R_q, conversely, indicates that the tunnelling pathway is unperturbed across the titration, which is the condition required for the C_q signal to be read as occupancy. It is an internal consistency criterion and not a proof: it cannot exclude non-specific adsorption or blocking that removes accessible centres without altering the relaxation of those that remain (for which a non-complementary control is the relevant test), changes in R_q below the resolution of the measurement (about 5% here; Section S8.5), or small shifts of the formal potential.

The amplified configuration and the isoscopic condition. In the FcA-amplified sensing configuration, the addition of 100 μM FcA to the electrolyte introduces free-redox states whose formal potential is aligned with the interfacial Pep-Fc states. This energy-level alignment enables electron transmittance from the bulk FcA/FcA$^+$ couple to the electrode through the interfacial quantum capacitive states, increasing the effective number of quantum channels N and thus $C_{q,0}$ at the blank interface. One interpretation of the concentration dependence of R_q in this configuration is that the progressive occupancy of the ssDNA receptor sites perturbs the Pep-Fc DOS and shifts its energy alignment with the FcA states, modifying both the effective electronic coupling κ in eq. S43 and the number of active channels N as a function of [L], giving a concentration-dependent $R_q = 2\pi(R_s + R_{qt})$ (eq. S42) that violates eq. S44. This interpretation is indirect, and the data do not exclude alternatives: electrostatic and steric exclusion of the mediator by the growing duplex layer, which would also reduce the number of active channels; and covariance between the faradaic branch and the slow low-frequency branch in the equivalent-circuit fit (Section S8.6). Experimentally, the concentration dependence is observed as a monotonic increase of R_q across the concentration range studied: $+1.10 \pm 0.25$ kΩ per decade ($p = 0.012$) by the equivalent-circuit route (Section S8.5). This violation does not invalidate the analytical calibration curve or the LOD/LOQ metrics of the amplified configuration, which remain valid as sensitivity characterisation. However, it means that the constant extracted from the amplified data is an *apparent amplified calibration parameter* of mixed origin, and its interpretation is correspondingly restricted.

The operational conclusion is that the R_q invariance test must be performed before any thermodynamic interpretation is attempted. It is recommended practice to: (i) verify R_q invariance from the same low-frequency EIS data used for C_q extraction; (ii) restrict thermodynamic (K_a) claims to configurations where invariance is confirmed, ΔG° being inaccessible

from the logarithmic intercept (Section S1.3); and (iii) label constants from non-invariant configurations explicitly as apparent amplified calibration parameters. The diagnostic guide of Section S11 sets this out in tabular form.

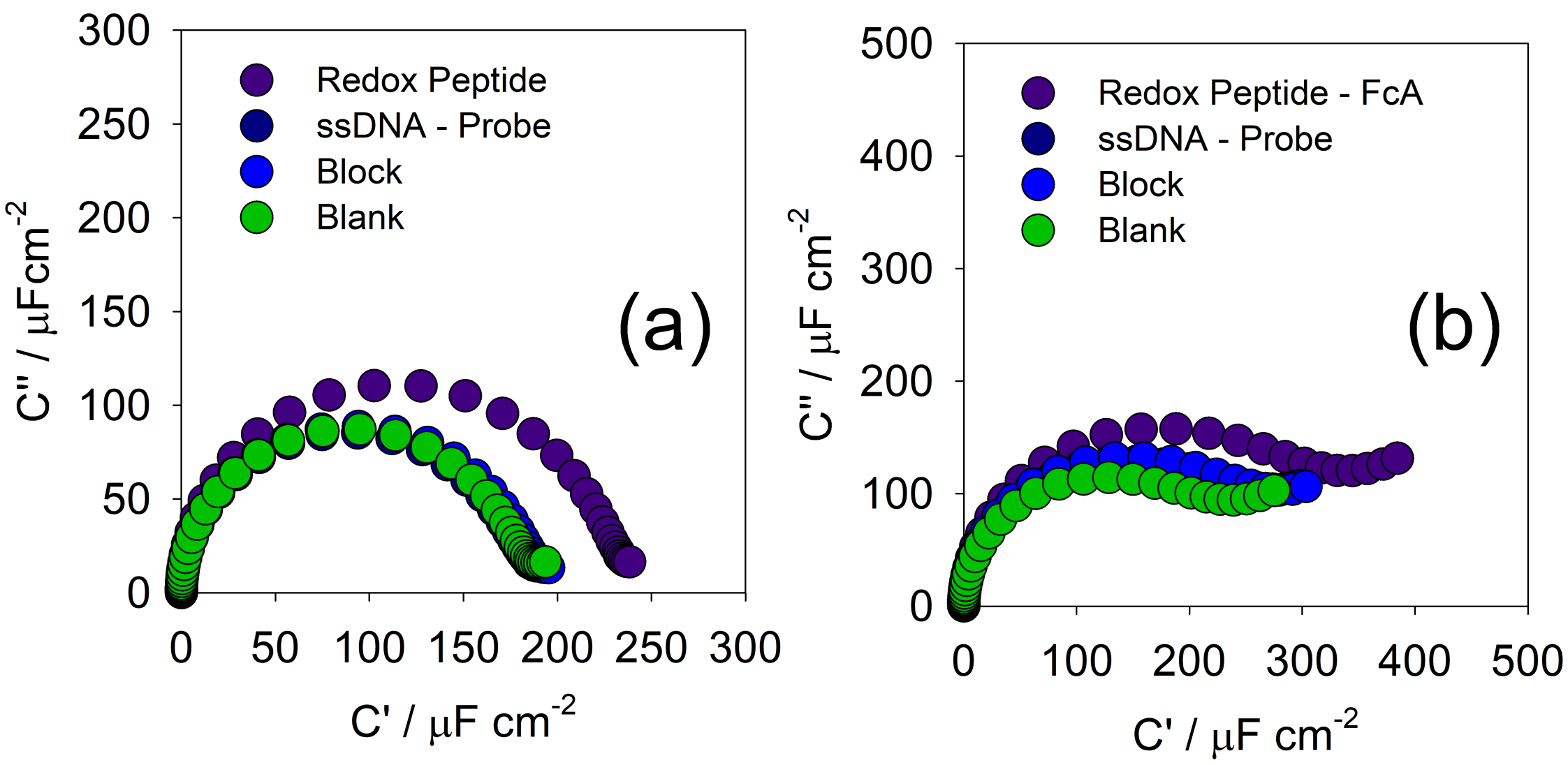

Figure S2: Nyquist capacitive diagrams (C'' vs. C', $\mu\mathrm{F\,cm^{-2}}$) recorded at each surface modification step for (a) the unamplified and (b) the amplified configurations, confirming reproducible and stepwise construction of the DNA-receptive interface. Each step produces a systematic decrease in the semicircle diameter (i.e., in C_μ, the directly measured electrochemical capacitance; $C_q = 2C_\mu$): Redox Peptide (purple) → ssDNA Probe (dark blue) → Block (blue) → Blank (green). The step on probe immobilisation is approximately 17% in (a) and approximately 15% in (b), where the ssDNA-Probe series is partly overlaid by the adjacent traces. Control parameters for interface quality are summarised in Table S2. Data from ref. 6.

Table S2: Control parameters obtained from voltammetry and impedance-derived capacitance spectra of redox-tagged peptide monolayers. Values correspond to the mean ± standard deviation for four independent electrodes. Following the convention used throughout this work, the directly measured quantity is the electrochemical capacitance C_μ — the diameter of the capacitive semicircle — and the quantum capacitance is inferred as $C_q = g_e C_\mu$ with $g_e = 2$. Ref. 6 denotes the semicircle diameter itself C_q and writes the relaxation $C^*(\omega) = C_q/(1 + j\omega\tau)$ with $\tau = R_q C_q$, no degeneracy factor being introduced; on the convention adopted here that quantity is C_μ, and both are given explicitly. Note that because RR(%) is a ratio, the factor g_e cancels from it and none of the affinity constants are affected by the change.

Parameter	Mean ± SD
E_F/e (V)	0.367 ± 0.008
C_μ (measured, μF cm^{-2})	243.3 ± 32.0
$C_q = 2C_\mu$ (inferred, μF cm^{-2})	486.6 ± 64.0
i_a (μA cm^{-2})	23.93 ± 4.22
i_c (μA cm^{-2})	-22.81 ± 3.98

These control values refer to the redox-tagged peptide monolayer *before* immobilisation of the ssDNA probe, and are areal. The per-concentration values of Table S4 are absolute, and the electroactive areas of the two electrode sets are 0.0515 ± 0.0070 cm^2 (unamplified) and 0.0460 ± 0.0098 cm^2 (amplified). Referred to those areas the blank sensing interface has $C_q = 360$ μF cm^{-2} on the convention $C_q = 2C_\mu$, against $C_q \approx 404$ μF cm^{-2} obtained by applying the 17% reduction on probe immobilisation to the control value of this table. The two agree to within 12%, the two being placed on a common basis by the C_μ/C_q distinction above; comparing the measured plateau directly with a quantity referred to C_q would introduce a spurious factor of two.

The control values of Table S2 are areal and serve as a measure of interface quality and reproducibility, for which purpose only relative changes are used; the per-concentration values of Table S4 are absolute. No quantity reported in this work requires the two to be placed on a common basis: R_q, χ, $\Delta\varepsilon$, α and the apparent interfacial constants are computed from absolute capacitance or from ratios, and none of them involves the electrode area.

S3.4. Correspondence Between Classical and Quantum Electrochemistry, and the Role of the Two Degeneracies

Two degeneracy factors appear in this work, g_e and g_s, and because both equal 2 it is easy to suspect double counting. They enter different observables for different reasons, and this section sets out the bookkeeping explicitly. It also establishes the correspondence between the voltammetric and impedimetric determinations of the same interfacial quantity.

The measured primitive. What an impedance experiment returns is the low-frequency plateau of the real complex capacitance, $P \equiv C'(\omega \to 0)$, in farads. For a single relaxation

channel the circuit relations are

$$\tau = R_q P, \qquad \Sigma = R_q P^2, \qquad \omega_r = 1/(R_q P), \tag{S45}$$

so that $R_q = \Sigma/P^2$ regardless of what P is subsequently called.

Büttiker and quantum-rate return the same R_q. The measured capacitance is the series combination $1/C_\mu = 1/C_e + 1/C_q$ (eq. S36), and the two frameworks differ in the limit assumed for the environmental term. In the mesoscopic (Büttiker) treatment $C_e \gg C_q$, so that $C_\mu \approx C_q$, the plateau is identified with the quantum capacitance, $C_q = P$, and the expansion is written $R_q = \Sigma/C_q^2$. Under the isoscopic condition $C_e = C_q$ one has $C_\mu = C_q/g_e$, the quantum capacitance is twice the plateau, $C_q = g_e P$, and the expansion is written $R_q = g_e^2\Sigma/C_q^2 = 4\Sigma/C_q^2$. The factor $g_e^2 = 4$ is exactly compensated by C_q being twice as large, and both reduce to $R_q = \Sigma/P^2$. The agreement with $h/2e^2$ reported here is therefore not an artefact of the convention adopted. This has been verified directly against the raw admittance data: regressing Re[G^*] against ω^2 through the origin over the stated window and computing Σ/C_μ^2 reproduces the tabulated R_q of Table S4 to within 10%, whereas Σ/C_q^2 would give a quarter of it. What does differ between the frameworks is the inferred C_q, by the factor g_e, and hence any quantity computed from C_q rather than from P.

The voltammetric correspondence. For a surface-confined, reversible one-electron couple the current at the formal potential is

$$i(V_F) = \frac{e^2\Gamma}{4k_\mathrm{B}T}\, s = \frac{F^2\Gamma}{4RT}\, s, \tag{S46}$$

where s is the scan rate. Comparison with $C_\mu = e^2N/4k_\mathrm{B}T$ gives

$$\frac{i(V_F)}{s} = C_\mu, \qquad \text{equivalently} \qquad g_e\, i(V_F) = C_q\, s, \tag{S47}$$

which is the correspondence between classical and quantum electroanalysis: the faradaic current at the formal potential *is* the electrochemical capacitance, the exchange current of the two channels taken together being the quantum capacitance. Two points should be stressed. Eq. S47 refers to the current at V_F and not to a peak current defined independently; and the factor of four in the denominator is $f(1-f) = 1/4$ at half-occupancy, that is, the same operating condition at which the entire impedance analysis is performed. The correspondence holds *at* V_F, and not through the shape of the voltammetric wave, whose width carries the dynamics of the potential sweep in addition to the equilibrium density of states; resolving the width of the density of states requires $C'(V)$ measured point-by-point at low frequency, which was not available for these monolayers.

Normalising the measured voltammogram of Figure S1(a) by the scan rate and reading it at $V_F = 0.367$ V gives $C_\mu(V_F) = 229$ μF cm^{-2} averaged over the two branches, against the tabulated 243.3 μF cm^{-2} of Table S2 — an agreement of 6%, which verifies eq. S47 on the raw data.

The identification $N = \Gamma$. Comparing $C_\mu = e^2N/4k_\mathrm{B}T$ with eq. S46 identifies the number of electrochemically accessible states with the coverage of redox centres, $N = \Gamma$ per unit area. This is a physical assumption rather than an algebraic identity: it states that the density of accessible states mirrors the redox population stoichiometrically, one accessible state per centre.

Why the spin degeneracy does not enter the capacitance. The two degeneracies attach to different observables. $g_e = 2$ is the isoscopic degeneracy, arising because at $C_e = C_q$ the ionic and quantum charging modes are energetically indistinguishable, and it enters the *capacitance*, $C_q = g_eC_\mu = e^2N/2k_\mathrm{B}T$. $g_s = 2$ is the spin degeneracy of the conduction channel, arising from the Landauer–Büttiker counting of transmission channels, and it enters the *resistance*, $R_{qt} = h/(g_se^2\kappa N)$. Because one is a property of charge storage and the other of charge transport, applying both is not double counting; conversely, neither may be invoked to account for a discrepancy in the other's observable, and in particular a factor of two in a measured capacitance cannot be attributed to spin.

Summary of what is convention-dependent. The relaxation resistance $R_q = \Sigma/P^2$, the number of accessible states $N = 4k_\mathrm{B}TP/e^2$ and the coverage $\Gamma = N/A$ are computed from the measured plateau and are independent of the framework adopted. The quantum capacitance C_q is not: it is P in the Büttiker limit and g_eP under the isoscopic condition. Wherever C_q appears in this work it is on the isoscopic convention, $C_q = 2P$.

S4. Apparent Interfacial Constants by Non-Linear Least Squares

S4.1. The Hanes–Woolf linearisation

The Hanes–Woolf linearisation — plotting [L]/RR(%) against [L] and reading $-K_d$ from the x-intercept — is not used in this work. Applied to RR(%) it estimates the same quantity, K_a^app, as the non-linear fit (main text), so the objection is not bias but leverage, which is why the two values differ (Figure S3 and Table S7).

Severe leverage from logarithmically spaced concentrations. The titration concentrations are spaced logarithmically, but the Hanes–Woolf plot places them on a *linear* abscissa. For six concentrations spanning five decades the leverage $h_{ii} = (x_i - \bar{x})^2/\sum_j(x_j - \bar{x})^2$ is:

[L] (relative)	10^0	10^1	10^2	10^3	10^4	10^5
h_{ii}	0.043	0.043	0.042	0.038	0.009	**0.825**

The highest concentration alone carries 82.5% of the abscissa variance and the lowest four points carry 16.6% combined. The apparent constant, obtained by extrapolation to an x-intercept lying well outside the sampled range, is therefore determined almost entirely by a single measurement. This is a property of the experimental design combined with the linearisation, not of the data quality.

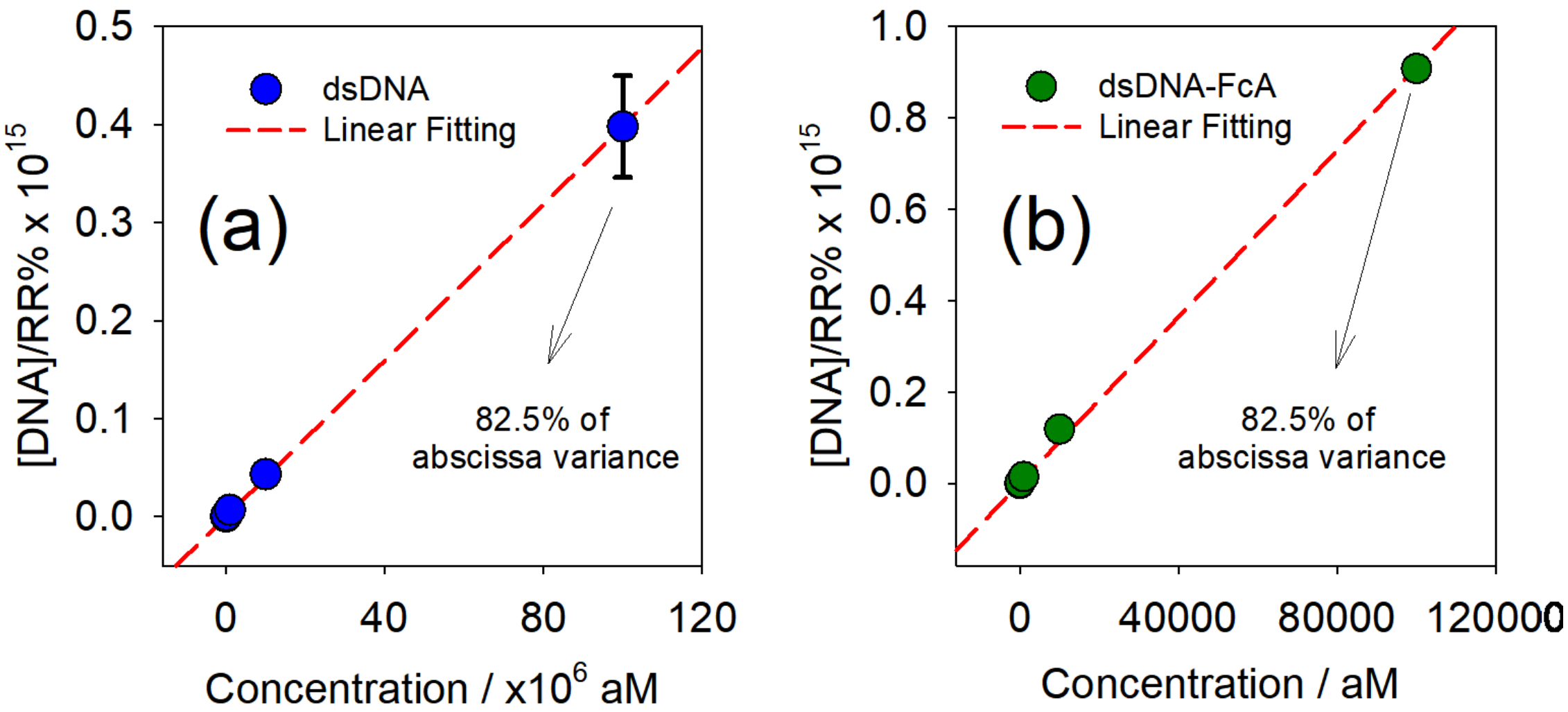

Figure S3: Hanes–Woolf linearisation of the calibration data ([L]/RR(%) $\times 10^{15}$ versus [L]) for (a) the unamplified dsDNA configuration and (b) the amplified dsDNA-FcA configuration, constructed from the calibration published in ref. 6. The constants read from the x-intercepts, 2.1×10^{12} and 1.3×10^{15} M^{-1}, are not used in the present work: the abscissa is linear while the concentrations are spaced logarithmically, so the highest point alone carries 82.5% of the abscissa variance and the intercept is fixed almost entirely by that one measurement. The apparent interfacial constants adopted instead are those of Table 2 of the main text, obtained by non-linear least squares on the untransformed response. Original analysis of the calibration data reported in ref. 6.

S4.2. Non-linear least-squares procedure

The apparent constant is obtained by fitting eq. S25 directly to the untransformed calibration data,

$$\mathrm{RR}(\%) = \frac{100\,\chi\,K_a[\mathrm{L}]}{1 + (1-\chi)\,K_a[\mathrm{L}]}, \tag{S48}$$

by unweighted non-linear least squares. Confidence intervals are obtained from the covariance matrix of the fit using the Student t multiplier for $n - 2$ degrees of freedom; because an affinity-type constant fitted to unsaturated data is constrained only on a logarithmic scale, the regression is carried out in $\log_{10} K$ and the intervals are given in decades (Table S8).

The fit is performed in an exactly equivalent two-parameter form. Eq. S48 is not fitted in the variables (χ, K_a), which are strongly correlated, but in the reparametrised form obtained by collecting the concentration-dependent terms,

$$\mathrm{RR}(\%) = S_\mathrm{m}\,\frac{K_a^\mathrm{app}[\mathrm{L}]}{1 + K_a^\mathrm{app}[\mathrm{L}]}, \qquad K_a^\mathrm{app} = (1-\chi)K_a, \qquad S_\mathrm{m} = \frac{100\chi}{1-\chi}, \tag{S49}$$

which is algebraically identical to eq. S48 at every point and is a plain hyperbola in [L]. Two consequences follow, and they settle what the fit does and does not deliver.

First, *no value of χ need be assumed in order to fit the data.* The constant returned by the regression is K_a^{app} directly — that is, the product $(1-\chi)K_a$ — and it is this quantity, not K_a, that the constants of Table 2 of the main text report. The saturation amplitude S_{m} is the second fitted parameter.

Second, χ is recoverable from the amplitude alone, through $\chi = S_{\mathrm{m}}/(100+S_{\mathrm{m}})$, and this is where the present titration fails: the amplitude of a hyperbola is determined by its plateau, and the data have none: the response is still rising between the two highest concentrations of both series (Section S8.7), while the best-fit hyperbola saturates inside the range, below the highest measured responses ($S_{\mathrm{m}} = 11.9\%$ against 15.0%, and 89% against 110%). S_{m} therefore does not estimate a plateau of the data, and carries a wide confidence interval ($11.9 \pm 3.8\%$ and $89 \pm 31\%$; Table S8). The χ inferred from it is correspondingly unreliable, and we do not use it. No other measurement in the present data supplies χ; what the data support is the lower bound of eq. S27.

Sensitivity of the affinity to the assumed transduction parameter. Because the regression determines K_a^{app} and not K_a, the dependence of the affinity on χ is not a property of the fit but an exact algebraic rescaling, $K_a = K_a^{\mathrm{app}}/(1-\chi)$. The table below tabulates it over the plausible range, taking K_a^{app} from Table 2 of the main text.

χ	$(1-\chi)^{-1}$	K_a, unamplified (M^{-1})	K_a, amplified (M^{-1})
0.131	1.15	1.7×10^{14}	1.6×10^{16}
0.169	1.20	1.8×10^{14}	1.6×10^{16}
0.25	1.33	2.0×10^{14}	1.8×10^{16}
0.40	1.67	2.5×10^{14}	2.3×10^{16}
0.50	2.00	3.0×10^{14}	2.7×10^{16}
0.524	2.10	3.2×10^{14}	2.9×10^{16}
0.625	2.67	4.0×10^{14}	3.6×10^{16}
0.75	4.00	6.0×10^{14}	5.5×10^{16}
0.90	10.0	1.5×10^{15}	1.4×10^{17}

Sensitivity of the affinity constant to the assumed transduction parameter, for $K_a^{\mathrm{app}} = 1.5 \times 10^{14}$ (unamplified) and 1.4×10^{16} M^{-1} (amplified), computed from the unrounded constants. The lower bound $\chi \geq (\chi\theta)_{\max}$ is 0.131 (unamplified) and 0.524 (amplified) on the published calibration to which these constants are fitted, and 0.169 and 0.625 on the capacitances of Table S4; entries below the bound for a configuration are excluded for it and shown only to display the trend.

At the bounds of the published calibration the correction is a factor of at least 1.15 for the unamplified configuration and 2.10 for the amplified one (1.20 and 2.67 on the capacitances of Table S4), and it would take $\chi > 0.9$ for the affinity to move by an order of magnitude. Because χ is not identifiable, only these lower bounds on the correction are available. The variation is in any case far smaller than that produced by the choice of functional form, which moves the constant by two to three orders of magnitude on the same data (Section S8.7). The rescaling does not convert a calibration parameter into an equilibrium binding constant: it corrects for partial transduction only, and leaves untouched the equilibrium premise that Section S9 identifies as the untested condition.

The procedure (a) uses the same observable as the theory, (b) applies no variance-distorting transformation, and (c) makes explicit, through χ, the difference between the fitted constant and the true affinity rather than absorbing it silently.

Because the linearisation returns K_a^{app}, the refitted constants differ from those obtained under the ideal-limit assumption by a factor $(1-\chi)^{-1}$, in addition to any change arising from the removal of the leverage artefact. The log-linear form, which describes the calibration over the range sampled (Section S8.7), returns 1.0×10^{17} M^{-1} (unamplified) and 3.9×10^{18} M^{-1} (amplified); the Langmuir form applied to the same data returns 1.5×10^{14} and 1.4×10^{16} M^{-1}. All are reported as calibration parameters, with the confidence intervals of Table S8, which for the Langmuir constants span about one decade on either side because the hyperbola describes the data poorly.

S4.3. Status of the logarithmic representation

The logarithmic plot of e^2/C_q against $\ln([\mathrm{L}]/[\mathrm{L}]_0)$ is retained in this work as a *diagnostic* of the transduction mechanism rather than as a route to K_a or ΔG°, for the reason given in Section S1.3: ε cancels from the adsorption-equilibrium condition by construction, so the intercept carries no affinity information. Its slope is given by eq. S17 and is interface-specific rather than universal.

A relation between the RR(%) calibration slope and $k_\mathrm{B}T/C_{q,0}$ alone would require the ratio of calibration slopes between two interfaces to equal the ratio of their blank capacitances. The measured values are inconsistent with this by a factor of $\sim$7 ($C_{q,0}^{\text{amp}} \approx 1.22\, C_{q,0}^{\text{unamp}}$ from Table S4, against a calibration slope ratio of ≈ 9), whereas eq. S25 accounts for the observed ratio through χ alone.

S4.4. What the logarithmic representation does not provide

Two logarithmic representations of the same data must be distinguished. The *thermodynamic plot* — e^2/C_q in absolute energy units versus ln[L] — carries the energy scale of the interface. The *analytical calibration curve* — RR(%) versus log[L], Figure 4 of the main text — has a slope in per cent per decade that is interface-specific and amplification-dependent, scaling with the blank quantum capacitance $C_{q,0}$. The two are related by an affine transformation and carry the same information. Neither slope is a universal constant, and no $k_\mathrm{B}T$ criterion follows from either.

Why the slope is not universal. The per-state charging energy is fixed at $2k_\mathrm{B}T$ (eq. S14), and the analyte dependence of the ensemble capacitance enters only through N (eq. S16). The slope of the logarithmic representation, eq. S17, therefore depends on χ and on the isotherm, and is interface-specific. It is not used to determine χ, which is bounded below by eq. S27 rather than measured.

The slopes of the dimensionless representation. These are 1.161×10^{-2} (unamplified) and 6.670×10^{-2} (amplified) per natural-logarithm unit. They should not be confused with the regression *intercepts* — the ordinate of the fitted line extrapolated to $\ln([\mathrm{L}]/[\mathrm{L}]_0) = 0$

— which carry no affinity information, since ε cancels from the equilibrium condition by construction.

Statistical significance of each panel. The trend in the unamplified panel of Figure 5 of the main text is not significant relative to its own error bars: the total rise is comparable to the individual uncertainties, corresponding to roughly 1.2σ. The amplified panel is highly significant ($\sim 7.6\sigma$). The representation is therefore a diagnostic of the transduction mechanism, not a validation of the occupancy relation.

Why the regression statistics differ between the two representations. Figure 5 of the main text gives $R^2 = 0.992$ and 0.923, whereas Figure 4 gives 0.998 and 0.994. The calibration curve reproduces the regression reported in ref. 6, while Figure 5 is regressed from the capacitance values re-read in the present work; the slopes differ correspondingly, 2.11% per decade against 3.00% per decade over the six concentrations of Table S4. Because RR(%) is a ratio, from which the degeneracy factor cancels, the difference does not arise from the $C_q = 2C_\mu$ convention. Nor does it arise from the criterion used to locate the closure of the capacitive semicircle, which the two analyses share: ref. 6 reads C' at the low-frequency minimum of C'', as does the present work.

Two effects account for it. The first is the *order in which electrodes are averaged.* RR(%) is a ratio, so forming it from the mean capacitances of Table S4 — the route used here — is not the same as forming it for each electrode against its own blank and then averaging, which is the route implied by the error bars of ref. 6. The two diverge whenever an electrode contributes to one row and not to another, as occurs at 10^8 aM, where the mean is over three electrodes and the blank over four. The second is the *reading of C' at the closure*, obtained graphically in ref. 6 and located algorithmically here on the measured frequency grid, without interpolation.

Neither effect bears on any conclusion of this work, as Section S7.1 shows: the constants of Table 2 of the main text are reported as calibration parameters with the confidence intervals of Table S8, and the bound on χ, although it differs between the two readings, leaves χ unidentified in both. It is, however, the reason the analytical figures of merit — the calibration slope per decade, the LOD and the LOQ — are quoted as reported in ref. 6 and have not been recomputed here.

S5. Detection Performance versus Thermodynamic Interpretability

There is a single route to the apparent interfacial constants. The logarithmic representation cannot deliver K_a, since the adsorption energy cancels from the equilibrium condition by construction (Section S1.3); the apparent constants are obtained by non-linear fitting of the occupancy expression (Section S4.2).

What remains genuinely dual, and is the organising idea of this work, is the distinction between *analytical detection performance* and *thermodynamic interpretability.* These are separate properties of an interface and they are not maximised by the same design, as set out in Table S3.

Table S3: Analytical detection performance and thermodynamic interpretability as independent axes of interface design.

	**Detection performance**	**Thermodynamic interpretability**
Question asked	What is the lowest concentration resolvable?	Is the signal a pure equilibrium occupancy report?
Figure of merit	Calibration slope, LOD, LOQ, dynamic range	R_q invariance; formal-potential stability; χ identifiable; equilibrium demonstrated
Governing parameter	Transduction parameter χ (larger is better)	Transduction parameter χ (must be known; the correction grows with χ)
Unamplified interface	$\chi \geq 0.169$; LOD in the fM range (previously reported)	R_q statistically invariant; consistent with occupancy transduction, but χ not identifiable
FcA-amplified interface	$\chi \geq 0.625$; LOD in the aM range (previously reported)	R_q rises with [L]; consistency criterion not met; χ not identifiable
Preferred application	Diagnostic screening	Affinity ranking and mechanistic study

The central point is that χ enters the two columns with opposite sign. A larger χ couples the binding event more deeply into the redox density of states, raising the calibration slope and lowering the detection limit; but it also increases the discrepancy $K_a^{\mathrm{app}} = (1-\chi)K_a$ (eq. S26), which can be corrected only if χ is identified — a factor $(1-\chi)^{-1}$ of at least 1.20 and 2.67 for the two configurations on the bounds of eq. S27 — and, in the FcA-amplified case, is accompanied by a concentration-dependent R_q that fails the internal consistency criterion. The amplified configuration therefore buys sensitivity at the cost of interpretability. This is a design trade-off rather than a defect, provided it is stated.

S6. Experimental Data Processing Algorithm

The following outlines the data processing workflow for extracting C_q, R_q, and the affinity parameters from raw EIS data files.

S6.1. Step 1: Import and Variable Conversion

(1) Import the EIS data matrix: frequency f (Hz), Z' (real impedance), Z'' (imaginary impedance).

(2) Calculate angular frequency: $\omega = 2\pi f$.

(3) Calculate the complex admittance: $G^*(\omega) = 1/(Z' + jZ'')$.

(4) Extract the real part of the admittance: $\mathrm{Re}[G^*(\omega)] = Z'/|Z|^2$.

(5) Calculate the complex capacitance: $C^*(\omega) = G(\omega)/(j\omega)$.

(6) Extract real and imaginary capacitance components:

$$C' = \frac{-Z''}{\omega|Z|^2}, \qquad C'' = \frac{Z'}{\omega|Z|^2} \tag{S50}$$

S6.2. Step 2: Determination of C_μ and C_q (Thermodynamic Parameter)

(1) Locate the closure of the capacitive semicircle as the first local minimum of C'' encountered on the low-frequency branch, on the measured frequency grid and without interpolation. If no such minimum exists within the measured window, the spectrum is excluded.

(2) Read the electrochemical capacitance C_μ as the value of C' at that point — this is the directly measured quantity (the Nyquist semicircle diameter):

$$C_\mu = C'(\omega_{\min}), \qquad \omega_{\min}: \text{ first local minimum of } C'' \tag{S51}$$

(3) Obtain the quantum capacitance from the isoscopic relation $C_q = g_e C_\mu = 2C_\mu$.

(4) No analyst choice enters this step; the response below $\omega_{\min}$ is excluded from all fits (Section S8.6).

S6.3. Step 3: Determination of R_q (Kinetic Parameter)

(1) Use the fixed low-frequency window stated in Table S4 (10 and 11 points), set in advance for each series below the relaxation frequency (upper point $f/f_r = 0.11$ for the unamplified series) and applied identically to every spectrum of that series.

(2) Perform a linear regression of $\mathrm{Re}[G^*(\omega)]$ vs. ω^2:

$$\mathrm{Re}[G^*(\omega)] = \Sigma\,\omega^2, \qquad \Sigma = R_q C_\mu^2 = \frac{R_q C_q^2}{4} \tag{S52}$$

(3) Extract: $R_q = 4\Sigma/C_q^2$, using C_μ from Step 2. Regress through the origin; state the frequency window (Table S4).

Figure S4 illustrates the $\mathrm{Re}[G^*(\omega)]$ vs. ω^2 plots for both sensing configurations. In both, the slopes $\Sigma = R_q C_\mu^2$ decrease with concentration because C_μ falls; R_q is the ratio Σ/C_μ^2, which is invariant in (a) and rises in (b).

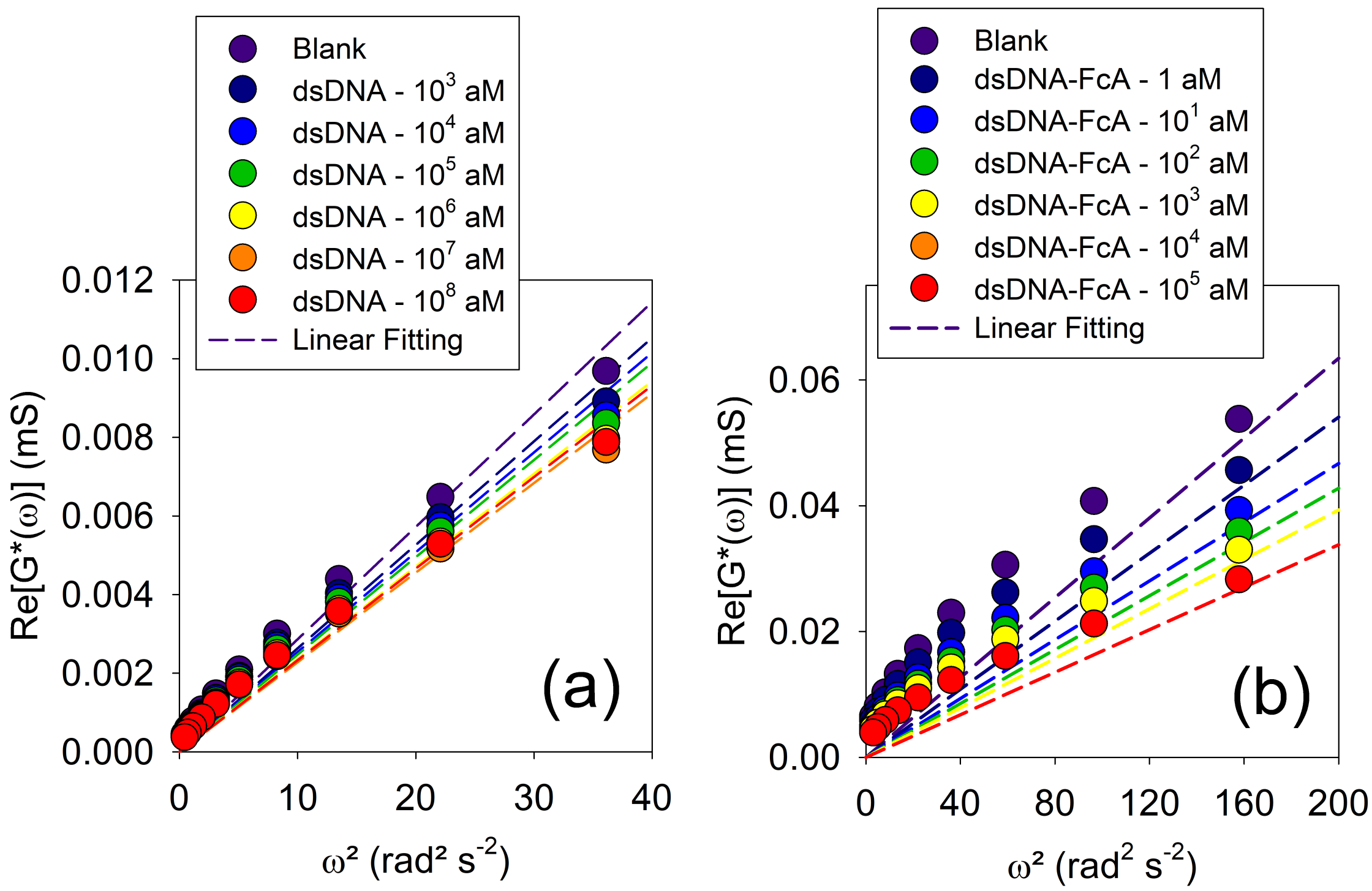

Figure S4: Low-frequency plots of the real part of the complex admittance $\mathrm{Re}[G^*(\omega)]$ (mS) vs. ω^2 ($\mathrm{rad}^2\,\mathrm{s}^{-2}$) for (a) the unamplified (dsDNA, 1 fM to 100,000 fM) and (b) the amplified (dsDNA-FcA, 1 aM to 100,000 aM) sensing configurations. Dashed lines are linear regressions through the origin; the slope of each fit equals $\Sigma = R_q C_\mu^2 = R_q C_q^2/4$ (eq. S52), where C_μ is the directly measured electrochemical capacitance (Nyquist semicircle diameter). Dividing each slope by C_μ^2, with C_μ determined independently as described above, yields R_q unambiguously for each titration point. In (a), the slopes decrease with concentration approximately in proportion to C_μ^2, so that R_q is invariant. In (b), the slopes decrease less than C_μ^2 does, which is the rise in R_q with [L]. These panels provide the complete spectroscopic basis for the R_q values plotted in Figure 6 of the main text. Data from ref. 6.

S6.4. Step 4: Affinity Matrix Consolidation

At each titration concentration $[\mathrm{L}]_i$, store the following derived quantities:

$$\left\{[\mathrm{L}]_i,\ C_{q,i},\ R_{q,i},\ \ln[\mathrm{L}]_i,\ \frac{1}{[\mathrm{L}]_i},\ \frac{1}{C_{q,i}},\ \frac{[\mathrm{L}]_i}{C_{q,i}},\ \frac{e^2}{C_{q,i}}\right\} \tag{S53}$$

Export the consolidated matrix and spectral vectors to a `.csv` file for plotting in LabPlot2 or equivalent software.

S7. Numerical Basis: Per-Concentration Data

Table S4 provides the numerical basis for the dispersion analysis and the R_q invariance test, so that these can be independently audited. *Scope of this table.* The quantities tabulated — the absolute electrochemical capacitance, the Cole–Cole exponent α and the two estimators of

R_q — are those not reported in ref. 6. The analytical figures of merit — the calibration slope in per cent per decade, the LOD and the LOQ — are taken from the calibration published in ref. 6 and have not been recomputed. The relative slope of Figure 5 of the main text, the products $\chi\theta$ and the bound on χ are computed from the capacitances of this table. *The isotherm fits are not*: the constants and goodness-of-fit statistics of Table 2 of the main text and of Table S7 are obtained from the published calibration of ref. 6, the same source as the figures of merit, and the two readings of the spectra are distinguished wherever both appear (Section S4.4).

Extraction procedure, stated algorithmically. C_μ is the real component of the complex capacitance at the closure of the capacitive semicircle, located as the first local minimum of C'' encountered on the low-frequency branch, on the measured frequency grid and without interpolation. The response below that point does not belong to the R_qC_μ relaxation and is excluded from all fits (Section S8.6); the Cole–Cole exponent α is accordingly fitted to the faradaic arc alone, over the interval running from the closure to 10^4 Hz. A spectrum is excluded when no such minimum exists within the measured window, so that the closure cannot be located; exactly one of the 49 spectra meets this condition. All capacitances are *absolute* values in farads; no electroactive-area normalisation is applied, because every derived quantity (N, χ, K_a, R_q) is computed from absolute capacitance.

Table S4: Per-concentration parameters for both sensing configurations. α is the Cole–Cole exponent of the electronic relaxation, obtained by fitting the faradaic arc alone (Section S7); R_{qt} is the relaxation resistance of the faradaic branch returned by the equivalent-circuit fit, reported separately in Section S8.4; R_q^{B} is obtained by the low-frequency (Büttiker) route, R_q^{C} by equivalent-circuit analysis. Errors are standard deviations across n independently prepared electrodes. The capacitance tabulated is the directly measured one, C_μ, read from the closure of the capacitive semicircle; the quantum capacitance follows as $C_q = g_e C_\mu = 2C_\mu$ under the isoscopic condition, and it is C_q that enters $N = 2k_{\mathrm{B}}TC_q/e^2$. Note that $\Sigma/C_\mu^2 = 4\Sigma/C_q^2$, so the extraction formula is unchanged. The mean at 10^8 aM is formed over three electrodes and the remaining rows over four; because RR(%) is a ratio of these means, this contributes to the difference between the calibration slope derived here and the one published in ref. 6 (Section S4.4), which is why the analytical figures of merit are quoted from that reference rather than recomputed.

[DNA] (aM)	C_μ (μF)	α	R_{qt} (Ω)	$R_q^{\mathrm{B}} = \Sigma/C_\mu^2$ (kΩ)	$R_q^{\mathrm{C}} = 2\pi(R_s+R_{qt})$ (kΩ)	n
Unamplified (dsDNA); window 0.105–0.956 Hz, 10 points						
Blank	9.27 ± 0.86	0.888 ± 0.042	65 ± 18	14.85 ± 1.55	13.01 ± 0.95	4
10^3	8.85 ± 0.83	0.884 ± 0.038	66 ± 19	14.80 ± 1.35	13.09 ± 0.85	4
10^4	8.56 ± 0.79	0.884 ± 0.040	66 ± 19	15.22 ± 1.42	12.91 ± 0.71	4
10^5	8.34 ± 0.74	0.881 ± 0.041	62 ± 28	15.76 ± 2.15	13.11 ± 0.64	4
10^6	8.10 ± 0.75	0.886 ± 0.040	63 ± 18	15.27 ± 1.46	12.88 ± 0.67	4
10^7	7.98 ± 0.73	0.878 ± 0.047	64 ± 17	15.53 ± 1.90	12.84 ± 0.47	4
10^8	7.70 ± 0.70†	0.922 ± 0.063	68 ± 18	15.93 ± 2.62	—	3
Amplified (dsDNA-FcA); window 0.219–2.557 Hz, 11 points						
Blank	11.35 ± 0.26	0.816 ± 0.038	126 ± 4	15.70 ± 1.52	18.75 ± 2.68	3
1	9.35 ± 0.61	0.838 ± 0.014	142 ± 5	19.39 ± 2.16	18.83 ± 3.88	3
10^1	8.40 ± 0.30	0.829 ± 0.023	153 ± 11	20.70 ± 1.85	19.32 ± 3.98	3
10^2	7.82 ± 0.56	0.840 ± 0.005	170 ± 15	23.67 ± 0.91	19.98 ± 3.83	3
10^3	7.22 ± 1.86	0.831 ± 0.015	186 ± 8	28.49 ± 5.87	19.98 ± 3.68	3
10^4	5.33 ± 1.38	0.825 ± 0.009	254 ± 35	39.57 ± 18.85	22.04 ± 2.27	3
10^5	4.26 ± 1.96	0.835 ± 0.008	748 ± 792	44.10 ± 17.70	24.88 ± 0.75	3

Excluded spectrum. †The exclusion rule applied throughout is stated here so that the table can be reproduced from the raw files: a spectrum is excluded when the low-frequency branch of C'' presents no local minimum within the measured window, so that the closure of the capacitive semicircle — and hence C_μ — cannot be located. Exactly one of the 49 spectra meets this condition, at 10^8 aM (electrode 247). Its failure is not marginal: re-reading the raw file under the algorithm above returns $C_\mu = 2.3 \times 10^{-5}$ μF, four orders of magnitude below the 7.04, 8.45 and 7.68 μF obtained from the other three electrodes of that row, whose mean, 7.72 μF, is the value tabulated. It is the same spectrum identified as anomalous in the original processing, where it returned $C_\mu = 5.41$ μF and $R_q = 35.7$ kΩ against 13.4, 15.7 and 18.7 kΩ for the others. Retaining it would raise the mean R_q of that row from 15.93 to 20.86 kΩ and its dispersion from 2.62 to 10.09 kΩ. The consequence of that row being averaged over three electrodes while the others use four is discussed in Section S4.4. This is

the only exclusion made; all other spectra are retained, including the two highest amplified concentrations, whose large inter-electrode dispersion is itself part of the result reported in Section S8.

The tabulated dispersions, and one absent entry. Every "±" in Table S4 is the standard deviation across the n independently prepared electrodes of that row, computed with $n - 1$ degrees of freedom; no other convention is used in this table. The dispersion *between* concentrations, which is the relevant quantity for a statement about invariance, is reported separately in Table S6. The entry for R_q^{C} at 10^8 aM is absent because the three-branch equivalent-circuit fit did not converge to a physically admissible solution for that row; the three retained spectra are otherwise unremarkable, giving $C_\mu = 7.04$, 8.45 and 7.68 μF and relaxation frequencies of 10.8, 8.7 and 8.7 Hz, so the omission reflects the fit and not the data. The low-frequency estimator, which requires no circuit model, is available for that row and is tabulated.

Statistical conventions. All regressions are *unweighted*; error bars were not used as fitting weights. Confidence intervals use the Student t multiplier for $n-2$ degrees of freedom rather than a single standard error. Uncertainty in C_μ propagates as $\delta(e^2/C_q) = (e^2/C_q)(\delta C_\mu/C_\mu)$ and $\delta R_q/R_q = \delta\Sigma/\Sigma + 2\,\delta C_\mu/C_\mu$. The equivalent-circuit fit uses multi-start optimisation. The non-faradaic branch is not obtained by free fitting: as established for this class of redox monolayer, the dielectric relaxation of the film is measured directly in a separate experiment, from the complex-capacitance spectrum acquired at a potential V_{out} outside the redox window, where the faradaic contribution is absent.[9, 11] The semicircle diameter of that spectrum gives C_t directly, without recourse to fitting. Under free optimisation the three-branch fit is degenerate — C_t can absorb part of the faradaic response, inflating it by an order of magnitude while leaving the residuals almost unchanged — so the independently measured value is imposed.

Magnitude of the dielectric contribution. The dielectric relaxation of the monolayer, $C_t \approx 5$ μF cm^{-2}, is measured independently at a potential outside the redox window as described above.[9, 11] It is compared here against the faradaic capacitance of the *present* interfaces, taken from the control measurements of Table S2, so that the argument is self-contained. Referred to the electroactive area 0.0515 ± 0.0070 cm^2, the blank sensing interface has $C_\mu = 180$ μF cm^{-2} and hence $C_q = 360$ μF cm^{-2} on the convention used throughout. The dielectric term is therefore approximately 1.4% of the faradaic capacitance, with the high-frequency limiting term $C_m \approx 2.6$ μF cm^{-2} at 0.7%. C_m is related to the double-layer capacitance but is not it: it is the limiting capacitance of the interface as ω becomes large, in which the ionic double layer appears in series with the low-permittivity monolayer and the latter dominates. Referring to it as the double-layer capacitance would misstate its origin, and would concede the solvent-determined reading of the signal that Section S12 and the invariance of R_q argue against. The faradaic capacitance dominates by roughly two orders of magnitude, which is the condition under which the approximations of Section S3 hold. Both quantities in this comparison are areal, so the ratio does not depend on the electroactive area.

More importantly, the argument does not rest on this ratio. C_t is measured outside the redox window, is independent of analyte concentration, and enters the response as a constant offset that cancels identically from RR(%). What argues against a dielectric transduction channel is the invariance of R_q and of α across the titration (Sections S8.3 and S8.4), not the relative magnitude of C_t. No area normalisation is applied anywhere in the analysis: every derived quantity (N, χ, the apparent constants and R_q) is computed from the absolute capacitances of Table S4.

S7.1. Robustness of the extraction

The calibration published in ref. 6 and the capacitances re-read here (Table S4) use the same criterion for C_μ, the value of C' at the low-frequency minimum of C''. They differ in the order of averaging — per-electrode responses averaged in ref. 6, mean capacitances here, with three rather than four electrodes at 10^8 aM — and in the graphical rather than algorithmic location of the minimum. The conclusions that depend on the calibration are the same under both readings:

	Published calibration	Re-read capacitances
Slope, unamplified (% per decade)	2.11	3.00
$(\chi\theta)_{\max}$, unamplified / amplified	0.131 / 0.524	0.169 / 0.625
ΔAIC for the log-linear form, unamplified / amplified	31.4 / 20.5	21.5 / 3.5

Under either reading χ is not identified and the log-linear form is preferred. R_q and α are computed spectrum by spectrum and do not depend on the reading. The inter-electrode dispersion of C_μ is 9% at every concentration of the unamplified series.

S8. Dispersion of the Electronic Relaxation: Langmuir as the Ideal Limit of Temkin

The treatment above assumes a single electronic relaxation time. This section removes that assumption. It shows that the Langmuir isotherm is the $\Delta\varepsilon \to 0$ limit of the Temkin isotherm, and examines how far the dispersion of the electronic relaxation R_qC_μ can be related to a distribution of site energies. That relation is one possible origin of the dispersion among several (Section S8.3), and it is not used to identify the adsorption regime.

S8.1. The two limits

The complex capacitance of the faradaic branch is written in the Cole–Cole form

$$C^*(\omega) = C_\infty + \frac{C_\mu - C_\infty}{1 + (j\omega\tau)^\alpha}, \qquad 0 < \alpha \le 1, \tag{S54}$$

where $\tau = R_qC_\mu$ and α measures the width of the distribution of relaxation times. Two remarks are essential. First, “Debye” here denotes the single-timescale *electronic* relaxation of eq. S54 at $\alpha = 1$, not dielectric relaxation of the film. Second, within the framework R_q

is a per-channel quantity fixed at $h/2e^2$ by the isoscopic condition and does not disperse, so that a dispersion confined to C_μ leaves it unchanged. A distribution of tunnelling distances or couplings, one of the alternative origins listed in Section S8.3, would not be confined to C_μ.

The corresponding limits in the concentration domain follow from the distribution of site energies. For a single site energy the isotherm is Langmuir; for a uniform distribution of site energies of width $\Delta\varepsilon$, centred on the energy corresponding to K_0, it is the Temkin isotherm,

$$\theta = \frac{k_\mathrm{B}T}{\Delta\varepsilon}\,\ln\frac{1 + K_0\,e^{\Delta\varepsilon/2k_\mathrm{B}T}[\mathrm{L}]}{1 + K_0\,e^{-\Delta\varepsilon/2k_\mathrm{B}T}[\mathrm{L}]}. \tag{S55}$$

With $x = \Delta\varepsilon/2k_\mathrm{B}T$ and $a = K_0[\mathrm{L}]$, the logarithm is odd in x, with first derivative $2a/(1+a)$ at $x = 0$, so that it equals $2xa/(1+a)+O(x^3)$. Hence $\theta \to K_0[\mathrm{L}]/(1+K_0[\mathrm{L}])$ as $\Delta\varepsilon \to 0$: the Langmuir isotherm is the ideal limit of eq. S55. Within the window $1/K_\mathrm{max} \ll [\mathrm{L}] \ll 1/K_\mathrm{min}$, where $K_\mathrm{max,min} = K_0 e^{\pm\Delta\varepsilon/2k_\mathrm{B}T}$, eq. S55 reduces to

$$\theta \simeq \frac{1}{2} + \frac{k_\mathrm{B}T}{\Delta\varepsilon}\,\ln(K_0[\mathrm{L}])\,, \tag{S56}$$

which is linear in $\ln[\mathrm{L}]$ and is meaningful only for $\Delta\varepsilon \gg k_\mathrm{B}T$; the window spans a concentration factor $e^{\Delta\varepsilon/k_\mathrm{B}T}$. The Langmuir limit must be taken in eq. S55, not in its asymptote. The correspondence is summarised in Table S5.

Table S5: Correspondence between the frequency and concentration domains within the theoretical framework: Langmuir is the $\Delta\varepsilon \to 0$ limit of Temkin (eq. S55). The correspondence between the two domains presumes that the dispersion of the electronic relaxation originates in a distribution of site energies, which is not established here (Section S8.3). The table sets out the structure of the two limits; it does not assign the present interface to either.

	**Ideal limit**	**General case**
Electronic relaxation	Debye, single τ	Cole–Cole, distributed τ
Cole–Cole exponent	$\alpha \to 1$	$\alpha < 1$
Presumed origin of dispersion	—	spread of site energies, expressed in C_μ
Adsorption isotherm	Langmuir	Temkin
Site energies	single value	distribution of width $\Delta\varepsilon$
Response vs ln[L]	sigmoidal	linear over a factor $e^{\Delta\varepsilon/k_\mathrm{B}T}$
Affinity constant	K_a, equivalent site	K_0, mean-energy site
R_q	$h/2e^2$	$h/2e^2$ (invariant)

S8.2. Quantitative bridge between the domains

The Cole–Cole distribution of relaxation times,

$$G(s) = \frac{1}{2\pi}\,\frac{\sin(\alpha\pi)}{\cosh(\alpha s) + \cos(\alpha\pi)}, \qquad s = \ln(\tau/\tau_0), \tag{S57}$$

has a full width at half maximum that grows as α decreases. If the dispersion originated solely in a spread of site energies, since $C_\mu \propto Nf(1-f)$ and a spread of site energies of width $\Delta\varepsilon$ produces a spread of $\ln C_\mu$ — and hence of $\ln\tau$ — of comparable width, the two domains would be related by

$$\Delta\varepsilon \;\approx\; k_\mathrm{B}T \cdot \mathrm{FWHM}[G(\ln\tau)]\,. \tag{S58}$$

Eq. S58 is an order-of-magnitude correspondence rather than an identity: it assumes that dispersion in $\ln\tau$ tracks dispersion in energy with unit coefficient. It further assumes that the whole of the dispersion arises from site energies. Because other origins can contribute to $\alpha < 1$ (Section S8.3), the width obtained from eq. S58 is an order-of-magnitude upper bound on the site-energy heterogeneity expressed in the electronic relaxation, and it is not used to identify the adsorption regime.

Applying eq. S58 to the measured exponents gives $\Delta\varepsilon \lesssim 20.1$ meV for the unamplified configuration ($\alpha = 0.888 \pm 0.042$) and $\lesssim 32.1$ meV for the amplified one ($\alpha = 0.816$–0.840). These correspond to $\Delta\varepsilon/k_\mathrm{B}T \lesssim 0.78$ and 1.25 respectively.

If a distribution of site energies were expressed in the electronic relaxation, the Temkin region of linearity in ln[L] would extend over a concentration factor $e^{\Delta\varepsilon/k_\mathrm{B}T}$, which for the values above is at most 2.2 and 3.5 — that is, 0.34 and 0.54 of a decade. The observed response is log-linear over five decades, which would require $\Delta\varepsilon \approx 296$ meV and hence $\alpha \approx 0.28$, against the measured 0.888. A site-energy distribution of the width compatible with α therefore does not account for the shape of the calibration.

We therefore do not assign these data to a Temkin regime. Nor do we infer from α that the receptor sites are equivalent: the dispersion is already present at the blank, before any site is occupied, and it admits origins other than site energies (Section S8.3). Section S9 states the consequence for the extracted constants.

S8.3. α is invariant with analyte concentration

If the dispersion reflects a static distribution of interfacial states, α should not change as the receptor sites are progressively occupied: binding changes the *occupancy* of the distribution, not its width. This is observed. Regressing α against log[DNA] for each unamplified electrode gives slopes statistically indistinguishable from zero in all four cases ($p = 0.19$, 0.12, 0.18, 0.54), with $|\mathrm{d}\alpha/\mathrm{d}\log[\mathrm{DNA}]| \leq 0.006$ per decade. The invariance of α is the frequency-domain counterpart of the invariance of R_q, and is consistent with occupancy-based transduction; it does not identify the origin of the dispersion.

Origins of the dispersion. A Cole–Cole exponent below unity is not specific to a distribution of receptor-site energies. Other contributions that the present data do not exclude are: geometric and microscopic heterogeneity of the polycrystalline electrode, the classical origin of constant-phase-element behaviour;[12, 13] a distribution of tunnelling distances or electronic couplings; distributed capacitive behaviour of the redox layer itself, that is, a spread of local formal potentials, coverages or lateral interactions;[14] and overlap with the slower low-frequency process (Section S8.6). The dispersion is already present at the blank ($\alpha = 0.888 \pm 0.042$, Table S4), before any receptor site is occupied, and it does not change as

sites are occupied. Neither observation favours an assignment to receptor-site energies, and none is made.

S8.4. R_q identifies the branch whose relaxation is being described

R_q is not inferred from these data. Within quantum-rate theory it is the charge-relaxation resistance of the coherent channel, fixed per channel at $h/2e^2$, and it is the quantity that defines the quantum signal of a redox monolayer; the framework is built on it rather than concluding it.[2, 3, 9] Its use here is therefore structural. The interface carries more than one dissipative process, and a depressed semicircle alone does not say which of them is being described — a distribution of molecular quantum states and the non-faradaic relaxation of the film give the same lineshape. Resolving the equivalent circuit and reading R_q on each branch settles which is which, because only the coherent channel is constrained to $h/2e^2$. For the unamplified configuration this returns $R_q = 12.97 \pm 0.72$ kΩ on the faradaic branch, against 15.34 kΩ from the low-frequency route applied to the whole semicircle; the faradaic branch is thereby identified at the quantum limit, and it is the dispersion of *that* branch which α describes.

The faradaic term separately. Because $R_q = 2\pi(R_s + R_{qt})$ combines the series resistance with the relaxation resistance of the faradaic branch, and because R_s would be expected to be constant across a titration performed in one cell, it is worth reporting R_{qt} on its own. Table S4 does so. In the unamplified configuration R_{qt} is 65 ± 18 Ω at the blank and 68 ± 18 Ω at 10^8 aM, with no trend across the five decades; in the amplified configuration it rises from 126 to 748 Ω. Since $R_{qt} = h/(g_s e^2 \kappa N)$, this is the term through which a change in the electronic coupling κ or in the number of channels N would appear, and the amplified trend is therefore located in the quantity on which the mechanism of Section S3.3 would act. It is not confined there: R_s also rises, from 2.86 to 3.21 kΩ, and carries about half of the R_q^{C} trend (0.50 of 1.10 kΩ per decade), which is consistent with the branch-covariance alternative listed in that section.

One qualification should accompany the unamplified result. Binding reduces the number of accessible states, so at constant κ the loss of N implied by the fall in C_μ would raise R_{qt} by some 20% across the titration. The inter-electrode dispersion of R_{qt} is itself close to 30%, so the measurement does not resolve an effect of that size. What the invariance of R_{qt} establishes is therefore that no *large* change in the tunnelling pathway accompanies hybridisation — it is consistent with an unperturbed pathway, and excludes the structural reorganisation that would produce a change of the magnitude seen in the amplified series — and not that κ is constant to the precision of the ratio N_0/N. The amplified trend, by contrast, exceeds the dispersion by a wide margin.

The identification of the branch is all that R_q provides here. It does not establish the physical origin of the dispersion of that branch (Section S8.3), and it does not choose the isotherm.

S8.5. Independent estimators of R_q

The routes to R_q used in this work are distinct measurements, not repeated calculations of the same quantity, and they are not expected to agree in absolute value.

Different regions of the same spectrum. The estimators do not read the same part of the spectrum, and the distinction matters for what each measures. The equivalent-circuit route is anchored at the *relaxation frequency* f_r, identified with the quantum rate through $\nu_\mu \tau = 1$: this is the apex of the capacitive semicircle, the point at which the electronic relaxation $\tau = R_q C_\mu$ is resonant with the perturbation, and it is from this condition that $R_q = 2\pi(R_s + R_{qt})$ follows. The Büttiker route operates instead in the *low-frequency equilibrium regime* $\omega\tau \ll 1$, well below f_r, where the expansion $\mathrm{Re}[G^*] \simeq \Sigma\,\omega^2$ is valid and the interface responds quasi-statically; the capacitance entering that expansion is C_q rather than C_μ, since the Büttiker treatment is written for the solid-state limit $C_q \ll C_e$, which is the origin of the factor $g_e^2 = 4$ in eq. S35. Both return R_q, but one reads it from the resonance condition and the other from the curvature of the dissipation as equilibrium is approached.

A third estimator, and a parameter-free test of the isoscopic condition. The quantum rate follows from the Planck–Einstein relation applied to the interfacial energy, $\nu_\mu = E_\mu/h$, and under the isoscopic condition takes the form

$$\nu_\mu = \frac{1}{R_q C_\mu} = \frac{g_e G_0}{C_q}, \qquad G_0 = \frac{1}{R_q} = \frac{2e^2}{h}, \tag{S59}$$

in which every quantity on the right is either a fundamental constant or the measured capacitance. Eq. S59 therefore *predicts* the relaxation frequency of the interface with no adjustable parameter: for the blank unamplified electrode, $C_q = 2C_\mu = 18.54$ μF gives $\nu_\mu = 8.36$ Hz. Measured directly as the apex of $C''(\omega)$, the relaxation frequency is $f_r = 8.65 \pm 0.11$ Hz — a deviation of 3.5%, and consistent with the value reported for redox peptide monolayers of this type.[9] We regard this as the most direct evidence available here that the interface satisfies the isoscopic condition, since it involves neither an equivalent-circuit decomposition nor a choice of extraction window.

Inverting eq. S59 supplies a third estimator of the charge-relaxation resistance, independent of both routes described below,

$$R_q = \frac{1}{\nu_\mu C_\mu} = \frac{1}{f_r C_\mu} = 12.5 \pm 1.3\ \mathrm{k}\Omega, \tag{S60}$$

which lies within 3.4% of $h/2e^2 = 12.906$ kΩ. The interval follows from $\delta R_q/R_q = \delta f_r/f_r + \delta C_\mu/C_\mu$ and is dominated by the inter-electrode dispersion of C_μ. Eq. S60 uses only two quantities read directly from the spectrum, the apex frequency and the semicircle diameter, and is offered for readers who prefer not to rely on a branch decomposition. The same relation predicts 6.83 Hz for the amplified blank; we have not evaluated it there, and note that agreement would not in any case be expected in a configuration which fails the R_q invariance test.

The extraction window used for the low-frequency route, 0.105–0.956 Hz, was fixed a priori to lie about one decade below this relaxation frequency (upper point $f/f_r = 0.11$), so that

the condition $\omega\tau \ll 1$ is met throughout, and it was applied identically to every spectrum of the series.

The low-frequency route is approximate here, and by how much. The quadratic expansion presumes that the electronic relaxation is the only dissipative contribution within the extraction window. The receptor-modified interface does not satisfy this exactly: a second, slower relaxation is present below ~0.5 Hz, and it contributes to $\mathrm{Re}[G^*]$ in the same window. Its magnitude can be read from the excess of C' below the minimum of C'', which amounts to some 5% of C_μ in the unamplified configuration and rises to over 100% in the amplified one, where 100 μM FcA is present in the electrolyte. Its effect on the fitted slope is measurable: regressing $\ln \mathrm{Re}[G^*]$ against $\ln \omega$ over the extraction window returns an exponent of 1.12 ± 0.14 for the unamplified series, against the value $1+\alpha = 1.89$ expected for a single Cole–Cole relaxation with $\alpha = 0.888$, and the departure from that value correlates with the amplitude of the slow feature across the data set ($r = -0.92$). The quantity Σ obtained by forcing an ω^2 dependence is therefore an approximation whose value depends on the window chosen, and R_q^{B} is correspondingly displaced from R_q, in the direction given by eq. S61 below. It is worth stating how large that window dependence is, since it is not a small effect and a reader may reasonably ask why one window rather than another. The local ratio $\mathrm{Re}[G^*]/\omega^2$, which would be constant for a pure quadratic response, rises by a median factor of five across the declared window as $\omega \to 0$, converging towards the true value only at the upper end, where the slow contribution has died away while $\omega\tau$ remains small. The window was not chosen to optimise the result: it was fixed a priori about one decade below f_r, as stated above, and applied identically to all spectra, and the resulting bias is consequently near-constant across the titration — which is what makes the estimator usable for a statement about invariance even though its absolute value is displaced. This is why the equivalent-circuit route, which resolves the non-faradaic branch explicitly rather than absorbing it, is used here as the primary estimator of R_q; the low-frequency route is retained as an approximate, independent check.

What both routes establish. The bias just described is essentially constant across the unamplified titration, because the slow contribution stays near 5% of C_μ at every concentration, and a constant bias does not affect a statement about invariance. Accordingly, both routes return an R_q that is statistically flat with analyte concentration in the unamplified configuration, and both therefore support the same conclusion: the C_q modulation observed on hybridisation carries no detectable kinetic contribution. In the amplified configuration the slow contribution is not constant — it grows with analyte concentration — so the low-frequency route exaggerates the observed rise in R_q; the trend reported in Section S8.5 and in the main text is the one returned by the equivalent-circuit route, which is not subject to this effect.

The *low-frequency* (Büttiker) route expands eq. S54 at $\alpha = 1$, $C^*(\omega) \approx C_\mu(1 - j\omega\tau)$, so that $\mathrm{Re}[G^*(\omega)] \simeq \Sigma\,\omega^2$ and $R_q = 4\Sigma/C_q^2$. It presumes a single relaxation channel and operates on a narrow frequency window, so the dissipation it reports is that of the *entire* semicircle, including the dielectric relaxation of the monolayer. The *equivalent-circuit* route fits the full spectrum with the non-faradaic branch (R_t, C_t) resolved separately from the faradaic branch (R_{qt}, C_μ), and returns $R_q = 2\pi(R_s + R_{qt})$ for the faradaic branch alone.

If a second branch is present it contributes to $\mathrm{Re}[G^*]$ at the same order in ω^2, so that

$$R_q^{\mathrm{B}} = R_q \left(1 + \frac{R_t C_t^2}{R_q C_\mu^2}\right) \;\geq\; R_q, \tag{S61}$$

with equality only when $R_t C_t^2 \ll R_q C_\mu^2$ — that is, in the single-branch (Debye) limit. Eq. S61 is a structural inequality establishing the direction and the condition of equality; it is not offered as a numerical correction, since the underlying expansion fails once the two branches have comparable time constants.

The diagnostic content lies in the *trend*, not in the absolute values (Table S6). For the unamplified interface both estimators are flat across the range each samples — 10^3–10^8 aM for R_q^{B} and 10^3–10^7 aM for R_q^{C} — and their ratio is constant at 1.13–1.21: a fixed multiplicative bias, as eq. S61 predicts when $R_t C_t^2 / R_q C_\mu^2$ does not vary. For the amplified interface, compared on the same basis (blank to 10^5 aM), the low-frequency estimator rises by 181% while the circuit estimator rises by 33%, and their ratio sweeps from 0.84 to 1.77, passing through a maximum of 1.80 at 10^4 aM. The corresponding regression trends, $+5.29 \pm 0.76$ against $+1.10 \pm 0.25$ kΩ per decade (Table S6), give the same factor of about five. The divergence between the two estimators is thus itself a measure of the dispersion, in the same sense that α measures it in the frequency domain.

Table S6: Behaviour of the two R_q estimators across the titration. The diagnostic is the trend and the ratio, not the absolute agreement. All entries are computed from the row means of Table S4 under a single convention: the mean is over concentrations, the two dispersions are the standard deviation between concentrations and the mean of the inter-electrode standard deviations respectively, and the trend is an unweighted regression on log[DNA] with "±" denoting one standard error and p from a Student t test on $n-2$ degrees of freedom. The unamplified R_q^{B} trend is marginal ($p = 0.058$) rather than null; the primary estimator R_q^{C} is flat ($p = 0.22$), and the residual drift in R_q^{B} is consistent with the window dependence quantified in Section S8.5.

	Unamplified		Amplified	
	R_q^{B}	R_q^{C}	R_q^{B}	R_q^{C}
Mean (kΩ)	15.34	12.97	27.37	20.54
SD between concentrations	0.43	0.11	10.71	2.21
SD between electrodes (mean)	1.78	0.72	6.98	3.01
Trend (kΩ/decade)	$+0.17 \pm 0.07$	-0.05 ± 0.03	$+5.29 \pm 0.76$	$+1.10 \pm 0.25$
p (trend $\neq 0$)	0.058	0.22	0.002	0.012
Ratio $R_q^{\mathrm{B}}/R_q^{\mathrm{C}}$	1.13–1.21 (constant)		$0.84 \to 1.77$, max 1.80 (diverging)	
α (between electrodes)	0.888 ± 0.042		0.831 ± 0.016	
α (between concentrations)	0.889 ± 0.015		0.831 ± 0.008	

S8.6. The low-frequency response below the semicircle

Below the closure of the capacitive semicircle both C' and C'' rise again. This response does not correspond to the $R_q C_\mu$ relaxation under analysis and is excluded from all fits. It is

not redox diffusion: across the 31 spectra examined, C' and C'' scale as $\omega^{-0.05}$ to $\omega^{-0.33}$, far from the $\omega^{-3/2}$ expected for a Warburg response, and the ratio C''/C' is 0.17–0.47 rather than unity. Its physical origin is not interpreted here. The frequency window used for each configuration is stated in Table S4.

S8.7. Isotherm comparison on the same data

Fitting both forms to the calibration data published in ref. 6 — not to the capacitances re-read in Table S4 — gives a decisive preference for the log-linear one in both configurations, by ΔAIC = 31.4 and 20.5. Both are computed as AIC $= n\ln(\mathrm{RSS}/n) + 2k$ with $n = 6$ calibration points and $k = 2$ parameters; since the two models have the same k, the small-sample correction is identical for both and cancels from ΔAIC. The Langmuir residuals show a systematic U-shaped pattern, the signature of forcing a saturating curve onto a response that is linear in ln[L]; the log-linear residuals show no such structure.

The comparison establishes the *shape* of the response: over the range sampled the data are log-linear and are not a Langmuir hyperbola. It does not establish a mechanism, and in particular it does not establish a Temkin regime: a site-energy distribution of the width compatible with the measured dispersion would not produce the log-linear shape (Section S8.2), and the origin of that dispersion is not established (Section S8.3). A functional form that fits is not thereby a physical model, and the constant returned by the log-linear fit is accordingly reported as a calibration parameter. The best-fit Langmuir isotherm saturates within the range sampled, below the highest measured responses, whereas the data do not.

Table S7: Langmuir and log-linear fits to the same calibration data, the published calibration of ref. 6. The constants are apparent interfacial constants, K_a^{app} (Langmuir) and K_{LL} (log-linear); their confidence intervals and parameter correlations are given in Table S8.

	Unamplified		Amplified	
	Langmuir	Log-linear	Langmuir	Log-linear
R^2	0.664	0.998	0.794	0.993
RSS	26.5	0.141	1303	42.7
AIC	12.90	−18.51	36.28	15.78
Constant (M^{-1})	1.5×10^{14}	1.0×10^{17}	1.4×10^{16}	3.9×10^{18}

On the apparent constants. The constant returned by the log-linear fit, K_{LL}, is not interchangeable with a Langmuir K_a for equivalent sites at equilibrium: it is the constant of a functional form that describes the data, not of a mechanism the data establish. Neither it nor K_a^{app} is called an affinity constant in the main text; both are reported as apparent interfacial constants, that is, as calibration parameters. The confidence intervals of the Langmuir constants span about one decade on either side of the central value (Table S8) because the hyperbola describes the data poorly: its best fit saturates within the range, whereas the relative response is still rising between the two highest concentrations in both series, by 2.5 (unamplified) and 26 (amplified) percentage points in the calibration of ref. 6. Extending the titration to higher concentrations would show whether the response saturates;

it would not by itself settle whether the response is an equilibrium isotherm, which is the separate question addressed in Section S9.

Table S8: Parameter estimates for the fits of Table S7, with 95% confidence intervals (Student t, $n-2=4$ degrees of freedom) and the correlation coefficient r between the two parameters of each fit. Langmuir form: $\mathrm{RR}(\%) = S_{\mathrm{m}}K[\mathrm{L}]/(1+K[\mathrm{L}])$; log-linear form: $\mathrm{RR}(\%) = m\log_{10}(K_{\mathrm{LL}}[\mathrm{L}])$, with m in % per decade. The constants are given on a logarithmic scale because unsaturated data constrain an affinity-type constant only in order of magnitude.

Form	Configuration	$\log_{10} K$ (M^{-1})	Second parameter	r	R^2
Langmuir	Unamplified	14.18 ± 1.04	$S_{\mathrm{m}} = 11.9 \pm 3.8\%$	−0.39	0.664
Langmuir	Amplified	16.14 ± 1.00	$S_{\mathrm{m}} = 89 \pm 31\%$	−0.45	0.794
Log-linear	Unamplified	17.02 ± 0.28	$m = 2.12 \pm 0.12$	−0.94	0.998
Log-linear	Amplified	18.59 ± 0.40	$m = 18.94 \pm 2.17$	−0.88	0.993

S9. Scope and Limitations

The equilibrium assumption, and the number of target molecules available. Every constant reported in this work presupposes that the calibration curve is an equilibrium occupancy curve. That premise is not demonstrated by these data, and two independent observations bear against it.

The first is the shape of the response (Section S8.7): a calibration log-linear over five decades is not the Langmuir isotherm of equivalent independent sites at equilibrium, and a distribution of site energies no wider than the bound obtained from the measured dispersion would not produce it (Section S8.2).

The second is the material balance. The number of accessible redox states at the blank unamplified interface is $N = 2k_{\mathrm{B}}TC_q/e^2 = 5.94 \times 10^{12}$, and the response at the lowest concentration of the amplified series corresponds to $\chi\theta = 0.176$, that is to some 1.0×10^{12} states rendered inaccessible. The incubation volume is 200 μL, so at 1 aM the entire sample contains 1.2×10^{2} target molecules. Even if every one of them reached the electrode, each would have to render some 8×10^{9} redox states inaccessible. Transport makes the discrepancy larger still: for an absorbing disc of the electroactive radius (0.128 cm) and a diffusion coefficient of 10^{-6} cm^2 s^{-1}, the Smoluchowski capture rate gives approximately *one* molecule arriving at the surface over the 1 h incubation, so the figure per captured molecule is of order 10^{11}. For comparison, the measured state density corresponds to 0.87 nm^2 per state, so a surface-tethered duplex can plausibly perturb of order twenty states. The same calculation at the lowest concentration of the unamplified series (1 fM) gives 1.2×10^{5} molecules available, $\sim 10^{3}$ captured, and $\sim 2\times10^{8}$ states per captured molecule; only at the highest concentration studied (100 pM) does the figure fall to $\sim 10^{4}$, which is still nearly three orders of magnitude above what a steric or electrostatic mechanism can supply.

These estimates are deliberately generous to the occupancy reading: the capture rate assumes a perfectly absorbing surface and neglects the finite association rate, and the elevated incubation temperature raises D by less than a factor of two. Convection during handling could raise the delivered number, but not by the eight to ten orders of magnitude required

at the lower end of either series. The relation $N = N_0(1 - \chi\theta)$ therefore cannot describe the response at low analyte concentration, whatever it describes at the upper end.

We do not resolve this here. Both observations are consistent with a response governed by delivered dose under transport-limited and effectively irreversible capture — each concentration being applied in a separate incubation, with hybridised duplex not dissociating on the timescale of the measurement — rather than by equilibrium occupancy. Testing it requires experiments this data set does not contain: demonstration of reversibility, variation of incubation time and volume at fixed concentration, and a titration extended until saturation is reached. Until then the constants reported in Table 2 of the main text should be treated as calibration parameters, and the analytical figures of merit — which do not depend on the equilibrium premise — as the results that stand independently of it.

The thermodynamic interpretation of the quantum-capacitance signal developed here is valid only under the conditions listed below. We state for each whether it is directly demonstrated in the present system, supported indirectly, or assumed. Table S9 classifies the quantities reported in this work by the assumptions each requires.

1. **Interfacial rather than bulk binding.** The constants reported are apparent interfacial constants, not bulk solution affinities. *Inherent to the method.*

2. **A specified adsorption regime.** The response is *not* Langmuir over the range sampled. The best-fit Langmuir isotherm saturates within the range sampled while the data do not; model comparison prefers the log-linear form by ΔAIC = 31.4 and 20.5 (Section S8.7), but a functional form is not a regime, and the dispersion exponent α does not identify one, since its origin is not established (Section S8.3). *Not identified.*

3. **The dispersion measured belongs to the faradaic branch.** A depressed semicircle is produced equally by a distribution of molecular quantum states and by the dielectric relaxation of the monolayer. The branch is identified by R_q, which for a coherent quantum channel is fixed at $h/2e^2$ and for a non-faradaic process is not. *Demonstrated* for the unamplified configuration (Section S8.4); the physical origin of the dispersion of that branch is not established (Section S8.3).

4. **A reliable free-concentration axis.** At attomolar and femtomolar nominal concentrations the number of target molecules in the incubation volume is small, and serial-dilution protocols, low-binding consumables, adsorption/recovery checks and independent verification of working concentrations were not part of the original experimental design. *Not verified*; the reported constants are therefore protocol-dependent.

5. **Equilibrium rather than transport-limited capture.** Langmuir affinity extraction requires an equilibrium interpretation of the concentration–response curve. The hybridisation protocol (1 h at 83 °C followed by cooling on ice for 5 min, with the impedance measured at room temperature)[6] is non-isothermal and is not an equilibrium titration: even an equilibrium reached during incubation would refer to a different temperature from that of the measurement. No incubation-time series or reversibility test is available, and cumulative or transport-limited capture cannot be excluded at the lowest concentrations. *Assumed, not demonstrated*; the constants are therefore apparent, protocol-dependent calibration parameters.

6. R_q **invariance (internal consistency criterion).** *Met* for the unamplified configuration, where any systematic change across 10^3–10^7 aM is below about 5% of R_q; *not met* for the amplified configuration, whose constants are therefore reported as apparent only. The criterion shows that the relaxation resistance does not change detectably on hybridisation. It does not exclude non-specific adsorption or blocking that removes accessible centres without altering the relaxation of those that remain, changes in R_q below about 5%, or small shifts of the formal potential.

7. **Formal-potential stability (electronic decoupling).** Expected from molecular-architecture reasoning and from literature precedent from related ferrocene-SAM systems, *not* verified by direct CV titration data across the DNA concentration series in this system. *Not verified directly.*

8. **An identifiable transduction parameter** χ**.** Required for K_a to be recovered from $K_a^{\text{app}} = (1-\chi)K_a$ (eq. S26); $\chi \ll 1$ is the special case in which no correction is needed. *Not satisfied*: χ is neither determined by the fit, since the plateau is not sampled, nor measured independently. It is only bounded below by eq. S27: $\chi \geq 0.169$ (unamplified) and $\chi \geq 0.625$ (amplified) from the capacitances of Table S4, corresponding to factors $(1-\chi)^{-1} \geq 1.20$ and 2.67; and $\chi \geq 0.131$ and ≥ 0.524 from the published calibration to which the constants of Table 2 of the main text are fitted, corresponding to factors of at least 1.15 and 2.10.

Table S9: Status of the quantities reported in this work: what each requires beyond the measured spectra.

Requirement	Quantities
Read directly from the spectra	C_μ; relaxation frequency f_r; RR(%); fractional change $1 - C_\mu/C_{\mu,0}$
A circuit or lineshape model	R_q, R_{qt}, α
The isoscopic condition	$C_q = 2C_\mu$; N
The occupancy relation $N = N_0(1-\chi\theta)$	Reading of $1 - C_\mu/C_{\mu,0}$ as $\chi\theta$; lower bound on χ (eq. S27)
An isotherm and equilibrium	K_a^{app}, K_d^{app}, K_{LL}
χ known	K_a — not available from these data

Long-term interface stability was not assessed in the original experimental study and is not claimed here. Detection of HNSCC-related sequences was performed in buffer as a proof of concept and does not constitute clinical diagnostic validation.

S10. List of Symbols

Symbol	Definition
C_μ	Electrochemical capacitance; *directly measured* (Nyquist semicircle diameter)
C_q	Quantum capacitance; *inferred* as $C_q = g_e C_\mu$ under the isoscopic condition
C_e	Environmental (ionic) capacitance of the electrolyte
g_e	Isoscopic degeneracy, $g_e = 2$
g_s	Spin degeneracy of the electron, $g_s = 2$
R_q	Charge-relaxation (quantum) resistance; quantum limit $h/2e^2 \approx 12.9$ kΩ
G_0	Conductance quantum, $G_0 = 1/R_q = 2e^2/h \approx 7.75 \times 10^{-5}$ S
ν_μ	Quantum rate, $\nu_\mu = E_\mu/h = 1/(R_q C_\mu) = g_e G_0/C_q$; a rate in s^{-1}, identified experimentally with the relaxation frequency f_r at the apex of $C''(\omega)$
Σ	Slope of $\mathrm{Re}[G^*(\omega)]$ versus ω^2; $R_q = \Sigma/C_\mu^2 = 4\Sigma/C_q^2$
τ	Macroscopic relaxation time, $\tau = R_q C_\mu$
τ_q	Quantum relaxation time, $\tau_q = R_q C_q = 2\tau$
c_q	Quantum capacitance per accessible state, $c_q = C_q/N$
N	Number of electrochemically accessible redox states, $N = 2k_\mathrm{B}TC_q/e^2$; binding removes no centres, so a fall in N is a loss of accessible occupancy of a fixed population
N_0	Number of accessible states at the blank interface, $N = N_0(1 - \chi\theta)$
N_t	Total number of receptor sites
θ	Fractional occupancy of receptor sites, $\theta = N_\mathrm{b}/N_t$
N_b	Number of bound ligands (Section S1.2); distinct from N
χ	Transduction parameter in $N = N_0(1-\chi\theta)$; the proportionality between occupancy and the fraction of redox centres rendered electrochemically inaccessible
ε	Standard free energy *required* for binding, per site
K_a, K_d	Equilibrium affinity and dissociation constants of the theory, $K_d = 1/K_a$; not obtained from these data
K_a^app	Apparent interfacial constant of the Langmuir form, $(1 - \chi)K_a$
K_d^app	Apparent dissociation constant of the Langmuir form, $1/K_a^\mathrm{app}$
K_LL	Constant of the log-linear form, $\mathrm{RR}(\%) = m \log_{10}(K_\mathrm{LL}[\mathrm{L}])$; a calibration parameter, not an affinity
S_m	Saturation amplitude of the calibration, $S_\mathrm{m} = 100\chi/(1 - \chi)$ (eq. S49)
[L], $[\mathrm{L}]_0$	Ligand concentration and reference concentration
S, S_0, S_i	Transducer signal $S = 1/C_q$; blank and at concentration i
RR(%)	Relative response, $100(S_i - S_0)/S_0$
V_F	Formal potential of the redox couple
E_F/e	Fermi level of the junction expressed as a potential
E_μ	Electrochemical energy, $E_\mu = e^2/C_\mu$; the change in electrochemical potential on charging the accessible states
E_0	Quantum energy at the blank interface, $E_0 = e^2/C_{q,0}$
η	Chemical hardness, $\eta \propto 1/C_q$ (Section S2)
A, Γ	Electroactive area and surface coverage, $\Gamma = N/A$; used only in the control comparisons of Section S3.4

continued on the next page

Symbol	Definition
P	Measured low-frequency plateau $C'(\omega \to 0)$; $P = C_\mu$ on the convention of this work (Section S3.4)
f	Fermi–Dirac occupancy function
κ	Electronic transmission coefficient, $\kappa = \exp(-\gamma L)$
γ, L	Tunnelling decay constant and tunnelling length (eq. S43)
β	$1/k_\mathrm{B}T$ (used for no other quantity in this work)
α	Cole–Cole exponent of the electronic relaxation; $\alpha = 1$ for a single relaxation time, $\alpha < 1$ for a distributed one
$\Delta\varepsilon$	Width of the uniform site-energy distribution (Temkin, eq. S55)
K_0	Temkin affinity of the site of mean energy
R_t, C_t	Resistance and capacitance of the non-faradaic branch (dielectric relaxation of the monolayer)
C_m	High-frequency limiting capacitance of the equivalent circuit; related to, but not identical with, the double-layer capacitance — the ionic double layer enters it in series with the monolayer, which dominates it
R_{qt}	Relaxation resistance of the faradaic branch, $h/(g_s e^2 \kappa N)$
R_s	Series resistance (electrolyte and contact contributions)
R_q^B	R_q from the low-frequency (Büttiker) route, $\Sigma/C_\mu^2 = 4\Sigma/C_q^2$
R_q^C	R_q from equivalent-circuit analysis, $2\pi(R_s + R_{qt})$, faradaic branch alone
C_∞	High-frequency limit of the Cole–Cole complex capacitance (eq. S54)
$G(s)$	Cole–Cole distribution of relaxation times, $s = \ln(\tau/\tau_0)$ (eq. S57)
g_amp	Blank-capacitance amplification factor, $C_{q,0}^\mathrm{amp}/C_{q,0}^\mathrm{unamp}$
2π	Conversion from circuit time constant to relaxation frequency, $\omega_r = 2\pi f_r$; appears in $R_q = 2\pi(R_{qt} + R_s)$
f_r, ω_r	Relaxation frequency, $\omega_r\tau = 1$; apex of the capacitive semicircle
$\omega \to 0$	Low-frequency equilibrium regime, $\omega\tau \ll 1$, where $\mathrm{Re}[G^*] \propto \omega^2$

S11. Expected Spectroscopic Signatures and Validation Panels

S11.1. Panel Descriptions and Expected Physical Behaviour

Panel (a) — Nyquist capacitive (C'' vs. C'). Near-ideal single-timescale electronic relaxation semicircles (Cole–Cole broadening of the electronic relaxation expected experimentally). The diameter C_μ — the directly measured electrochemical capacitance — decreases systematically with increasing [L], reflecting the decrease in accessible DOS upon ligand occupancy of the receptor sites; the quantum capacitance is $C_q = 2C_\mu$. Semicircles should originate near $C' = 0$.

Panel (b) — Bode conductance ($\mathrm{Re}[G^*(\omega)]$ vs. f). If C_q decreases with [L] and R_q remains constant (isoscopic regime), the relaxation timescale $\tau = R_q C_\mu = R_q C_q/2$ decreases, shifting the conductance peak to higher frequencies with increasing [L].

Panel (c) — Calibration isotherm (RR(%) vs. [L]). Under the occupancy model, hyperbolic saturation described by eq. S25, fitted by non-linear least squares; linearised forms

are not used, for the reasons given in Section S4.1. Note that χ and K_a are not separately identifiable from a free two-parameter fit unless the titration reaches saturation, and that the present data do not show the expected saturation at all — see Section S8.7.

Panel (d) — Quantum energy (e^2/C_q vs. $\ln([\mathrm{L}]/[\mathrm{L}]_0)$). Monotonic increase, reflecting the reduction of the accessible density of states upon binding and hence increasing chemical hardness $\eta \propto 1/C_q$. The slope is given by eq. S17 and is interface-specific; it is *not* expected to equal $k_\mathrm{B}T$. This panel is descriptive: its slope is interface-specific rather than universal (Section S1.6), and its intercept depends only on the reference concentration, since ε cancels from the adsorption-equilibrium condition by construction — so neither K_a nor ΔG° can be read from it.

Panel (e) — Isoscopic invariance (R_q vs. $\ln[\mathrm{L}]$). A horizontal line (statistically constant R_q within experimental error) across all titration points. This is the internal consistency criterion for occupancy-dominated transduction; it constrains the origin of the C_q modulation and does not by itself establish a thermodynamic reading of the extracted constants.

S11.2. Diagnostic Guide for Non-Ideal Behaviour

Table S10 summarises the deviations from ideal isoscopic behaviour that may be encountered and their probable causes.

Table S10: Diagnostic guide: observed deviations from ideal isoscopic behaviour and their physical interpretation.

**Observation**	**Probable cause / action**
Response not log-linear, or curvature inconsistent with either limiting isotherm	Three distinguishable failure modes (see SI Table S1): (i) *Site heterogeneity* — indicated when both R_q invariance and V_F stability tests pass *and* the dispersion exponent is well below unity; a distribution of site energies is only one of several possible origins of $\alpha < 1$ (Section S8.3), so α alone does not establish it; the width from eq. S58 is an upper bound (Section S8.2). (ii) *Formal potential channel contamination* ($\|\Delta V_F\| \gtrsim k_\mathrm{B}T/e \approx 25.7$ mV) — indicated when the CV titration shows a shift in E_F/e with [L]; the extracted K_a is a redox-state-dependent apparent affinity, not a thermodynamic constant. (iii) *Kinetic/structural artefacts* — indicated when the R_q invariance test also fails; check for non-specific adsorption or film degradation. Note: the slope of the thermodynamic plot is not a diagnostic in this regime, being interface-specific rather than universal (Section S1.5); the CV V_F check is the definitive test.
V_F shifts with [L] (CV titration)	Formal potential channel active: analyte is electronically coupled to the redox centre through direct or through-bond interaction, shifting the ferrocene oxidation energy. This is the intended transduction mechanism in voltammetric anion sensing[5] but a disqualifying artefact in the quantum-capacitance thermodynamic framework. The occupancy relation alone does not describe the signal and K_a extraction is not supported. Redesign the receptor architecture to increase spatial separation between the recognition and redox centres.
R_q increases with [L]	Structural thickening of the monolayer (e.g., conformational change upon binding) or non-specific blocking of the interface. Also observed in FcA-amplified configurations, where it is consistent with, but not shown to arise from, concentration-dependent modification of the FcA transmittance channels; exclusion of the mediator by the duplex layer and branch covariance in the circuit fit are alternatives (Section S3.3). In this case the analytical calibration is valid, but thermodynamic (K_a) extraction is not supported.

continued on the next page

Table S10 (continued)

**Observation**	**Probable cause / action**
R_q decreases with [L]	Partial monolayer dissolution or desorption of redox tags at high analyte concentrations. Verify by post-assay CV.
Response log-linear over more than two decades	Inconsistent with any single Langmuir isotherm, for which $\mathrm{d}\ln\theta/\mathrm{d}\ln[\mathrm{L}] = 1 - \theta$ cannot stay constant over a wide range. A distributed-energy isotherm may be considered, but α alone does not establish one (Section S8.3), and the equilibrium assumption itself should be tested: verify that the response is reversible and that the number of target molecules delivered is sufficient to occupy the receptor sites at the concentrations used.
Nyquist semicircle does not close at low frequency	Insufficient low-frequency range in EIS acquisition. Extend to lower f_{min}.

S12. Impedance Spectra of the Two Sensing Configurations

This section gives the full description of the impedance spectra summarised in the Results and Discussion of the main text (Figures 2 and 3).

S12.1. Concentration ranges and spectral form

Figure 2 of the main text presents the unamplified configuration for target dsDNA concentrations from 10^3 aM to 10^8 aM (equivalently 1 fM to 10^5 fM, or 1 fM to 100 pM); Figure 3 presents the amplified (FcA-assisted) configuration from 1 aM to 10^5 aM.

The near-ideal semicircular relaxation in panel (a) of Figure 2 indicates that the unamplified interface behaves as a coherent quantum transducer whose relaxation dynamics are governed by a single R_qC_μ timescale (eq. S54), consistent with the isoscopic condition. Panel (a) of Figure 3 shows a more depressed arc ($\alpha = 0.83$), followed by a slow low-frequency feature (Section S8.5). Its shape does not establish equivalent behaviour: as the R_q analysis shows, the amplified configuration fails the R_q consistency criterion. The distinction between the two configurations lies in the R_q behaviour and not in the Nyquist profile.

In both configurations the semicircle diameter — that is, C_μ — decreases systematically with increasing target concentration. This is attributed to hybridisation of the target dsDNA at the interface, which reduces the electrochemical accessibility and occupancy of the redox states of the peptide–ferrocene monolayer. The decrease is consistent with eq. S16: as θ increases, the number of accessible states $N = N_0(1 - \chi\theta)$ falls, and the quantum energy $e^2/C_q \propto 1/\mathrm{DOS}$ rises, which is the analytical signal. The amplification afforded by FcA addition appears as a larger absolute C_μ together with a proportionally enhanced relative response per unit concentration change, consistent with alignment of the external redox probe with the interface energy state $E = e^2/C_q$.

S12.2. Surface-modification controls and blank reproducibility

Prior to DNA sensing, the quality and reproducibility of each interface were verified by tracking the change in C_μ across successive surface-modification steps (Figure S2). Immobilisation of the ssDNA probe over the redox-tagged peptide monolayer produced a decrease of approximately 17% for the unamplified configuration and approximately 15% for the amplified one, confirming reproducible chemical modification of the electrode surface in both cases.[6]

Blank reproducibility was confirmed by repeated measurements in hybridisation buffer without target DNA, subjected to the same thermal cycle as the samples (1 h at 83 °C followed by 5 min on ice), yielding RR% variations of only 0.2–0.9% across consecutive measurements. This is well below the smallest measured response of the titration series — 4.35% at 10^3 aM (unamplified) and 14.62% at 1 aM (amplified)[6] — and establishes the signal-to-noise baseline against which the calibration is read.

S12.3. Real admittance and the basis for R_q extraction

Panels (c) and (d) of Figures 2 and 3 display $\mathrm{Re}[G^*(\omega)]$ over the full spectral range and at low-frequency zoom respectively. These provide the experimental basis for extracting R_q from the low-frequency ω^2 slope (Section S8.5).

In both configurations the low-frequency curves decrease overall with concentration, because their ω^2 slope is $\Sigma = R_q C_\mu^2$ and C_μ falls. R_q is the ratio Σ / C_μ^2: it is invariant in the unamplified configuration and rises in the amplified one, for which perturbation of the FcA transmittance channels is one interpretation among those discussed in Section S3.3. The quantitative test is given in the main text.

References

[1] Bueno, P. R. Quantum electroanalysis in drug discovery. *Chem. Commun.* **2025**, *61*, 8632–8642.

[2] Vignotto, R. F. T.; Pinzón, E. F.; Bueno, P. R. Grand Canonical Equilibrium Unifies Quantum Electrodynamics and Statistical Mechanics in Wet Electrolytic Environments at Room Temperature. *Phys. Chem. Chem. Phys.* **2026**, *28*, 21345–21358.

[3] Bueno, P. R. A unified quantum rate theory of electron transfer: conceptual advances in quantum electrochemistry. *Chem. Soc. Rev.* **2026**, *55*, 1803–1812.

[4] Bueno, P. R.; Bedatty Fernandes, F. C.; Davis, J. J. Quantum capacitance as a reagentless molecular sensing element. *Nanoscale* **2017**, *9*, 15362–15370.

[5] Hein, R.; Beer, P. D.; Davis, J. J. Electrochemical Anion Sensing: Supramolecular Approaches. *Chem. Rev.* **2020**, *120*, 1888–1935.

[6] Carr, O.; Pinzón, E. F.; Santos, A.; Faria, R. C.; Bueno, P. R. Attomolar sensitivity of a redox capacitive and DNA-receptive interface attained by quantum-rate signal amplification concept. *Biosens. Bioelectron.* **2025**, *270*, 116910.

[7] Langmuir, I. The adsorption of gases on plane surfaces of glass, mica and platinum. *J. Am. Chem. Soc.* **1918**, *40*, 1361–1403.

[8] Hill, A. V. The possible effects of the aggregation of the molecules of haemoglobin on its dissociation curves. *J. Physiol.* **1910**, *40*, iv–vii.

[9] Pérez Sánchez, Y.; Santos, A.; Bueno, P. R. Quantum Mechanical Meaning of the Charge Transfer Resistance. *J. Phys. Chem. C* **2022**, *126*, 3151–3162.

[10] Garrote, B. L.; Sánchez, Y. P.; Lopes, L. C.; Santos, A.; Bueno, P. R. Electron transmittance by means of quantum capacitive states as a signal amplification mechanism for biosensing applications. *Sens. Actuators B* **2024**, *399*, 134786.

[11] Góes, M. S.; Rahman, H.; Ryall, J.; Davis, J. J.; Bueno, P. R. A Dielectric Model of Self-Assembled Monolayer Interfaces by Capacitive Spectroscopy. *Langmuir* **2012**, *28*, 9689–9699.

[12] Brug, G. J.; van den Eeden, A. L. G.; Sluyters-Rehbach, M.; Sluyters, J. H. The Analysis of Electrode Impedances Complicated by the Presence of a Constant Phase Element. *J. Electroanal. Chem.* **1984**, *176*, 275–295.

[13] Jorcin, J.-B.; Orazem, M. E.; Pébère, N.; Tribollet, B. CPE Analysis by Local Electrochemical Impedance Spectroscopy. *Electrochim. Acta* **2006**, *51*, 1473–1479.

[14] Laviron, E. The Use of Linear Potential Sweep Voltammetry and of A.C. Voltammetry for the Study of the Surface Electrochemical Reaction of Strongly Adsorbed Systems and of Redox Modified Electrodes. *J. Electroanal. Chem.* **1979**, *100*, 263–270.
